# Chlorine dioxide gas sterilization of cold-chain or temperature-sensitive devices

**DOI:** 10.1371/journal.pone.0321345

**Published:** 2026-08-27

**Authors:** Emily Lorcheim, Paul Lorcheim, Kirk Livingston

**Affiliations:** 1 Vice President of Sterilization Technologies, ClorDiSys Solutions, LLC, Branchburg, New Jersey, United States of America; 2 Founder, ClorDiSys Solutions, LLC, Branchburg, New Jersey, United States of America; 3 President of Livingston Communication, Inc, St. Paul, Minnesota, United States of America; Hamadan University of Medical Sciences, IRAN, ISLAMIC REPUBLIC OF

## Abstract

Cold-chain terminal sterilization is used for products that must be manufactured, stored, and maintained with a carefully controlled temperature range, such as temperature-sensitive drugs and vaccines in prefilled syringes or autoinjectors containing temperature-sensitive drugs. Chlorine dioxide (ClO_2_) is an ideal sterilant for cold-chain terminal sterilization because it forms a true gas at room temperatures (15°C/59°F – 25°C/77°F), with a reliable and consistent process, with excellent penetration, and does not require elevated temperatures. In the current study, we validated the efficacy of ClO_2_ on cold-stored prefilled syringes as well as tested ClO_2_-sterilized samples and non-sterilized controls for residual sterilant on the prefilled syringe samples and the ingress of sterilant into the prefilled syringe samples. Residual sterilant remaining on a load after sterilization could cause chemical exposure to patients and negatively affect healthcare worker safety, among other things. Sterilant ingress into a medication could result in chemical contamination, drug degradation, and changes to drug formulation. Few studies have been published on ClO_2_ residue or ingress during terminal sterilization. Through this descriptive analysis of residual and ingress testing results, we demonstrate that chlorine dioxide sterilization leaves chlorite and chloride residuals at acceptably low levels. The residual chlorite average was 0.74 mg/L (control) and 0.82 mg/L (sterilized), which are below the maximum contaminant acceptable level of 1.0 mg/L. We also demonstrated that there was no evidence of any ingress,and that the amounts remained at acceptably low levels: average ingress chlorite was 0.78 mg/L (control) and 0.72 mg/L (sterilized), below the maximum contaminant acceptable level of 1.0 mg/L. Chlorate has no known maximum contaminant level. ClO_2_ sterilization is an excellent alternative to ethylene oxide sterilization for cold-chain sterilization because it is sustainable, non-carcinogenic, and does not leave harmful residuals, nor does it ingress into the drug product. This study helps demonstrate that ClO_2_ is a viable primary or alternative sterilization modality for medical device manufacturers.

## Introduction

Effective sterilization renders an object “free from viable microorganisms” [1,2]. Terminal sterilization of medical devices ensures microbial inactivation to a sterility assurance level (SAL) of 10^−6^ for regulatory purposes [3]. Except for terminal sterilization by irradiation, typical sterilization modalities, like ethylene oxide (EtO), require higher temperatures to be effective. High temperatures can compromise temperature-sensitive medical devices, their components, and other medical products that must be stored and transported while maintaining a specific temperature range. The objective of this study was to demonstrate the utility of chlorine dioxide (ClO_2_) when following cold-chain protocols. These studies demonstrate that ClO_2_ sterilization is able to sterilize such devices at ambient temperatures, which does not degrade the drug product. Residuals and ingress issues are also critical with all sterilization methods, so these studies also explored ClO_2_ residuals and ingress in cold-chain sterilization since few studies have been undertaken up to this point.

“Cold chain” is a set of rules and procedures that help ensure products such as vaccines and medical devices are manufactured, stored, or transported within a controlled temperature range. Cold chain sterilization is a set of parameters requiring that low temperatures be maintained over a controlled range throughout the process.

ClO_2_ is an ideal sterilization modality for cold chain sterilization because it has high efficacy in penetration and sterilization without elevating temperatures. ClO_2_ sterilization cycles are processed at ambient temperature and typically last 4–6 hours. These cycles do not generate excessive temperatures and keep the product from experiencing damaging temperatures by limiting the product’s time-out-of-refrigeration (TOR). Maintaining temperature parameters is of concern for a significant number of industries [4]. Also, unlike EtO, the devices do not require aeration at elevated temperatures to remove sterilant residues.

Acceptable residual levels for EtO are documented in ISO 11135 and well-established within the sterilization industry. Since ClO_2_ is a newer technology, acceptable residual levels have not yet been documented. In the current study, we tested ClO_2_-sterilized samples and non-sterilized controls for residual sterilant and ingress of sterilant into the prefilled syringe samples. To date, limited studies have quantified residuals or ingress in cold-chain sterilization.

Sterilant ingress into a medication could result in chemical contamination, drug degradation, and changes to drug formulation. Residual sterilant remaining on a load after sterilization could cause chemical exposure to patients and negatively affect healthcare worker safety, among other things. This study demonstrates that chlorine dioxide sterilization leaves residuals and ingress amounts at acceptably low levels (under 1.0 mg/L). We also demonstrate that ClO_2_ is an excellent alternative terminal sterilization choice by virtue of its efficacy at ambient temperature parameters and low levels of residue or ingress. Terminal sterilization at ambient temperatures, or cold-chain sterilization, is a critical need for manufacturers producing combination products. ClO_2_ provides a useful alternative to current terminal sterilization modalities.

## Materials and methods

### Materials

- 1800 prefilled syringes (PFS) (Type 1 HPLC Grade distilled water) prepared under controlled conditions to be used as a maximum load.
- 9 internal process challenge devices (iPCDs) with biological indicators (*Geobacillus stearothermophilus*) placed inside. iPCDs by ClorDiSys (New Jersey, USA).
- 9 external process challenge devices (ePCDs) with biological indicators (*Geobacillus stearothermophilus*) placed inside. The housing was produced by ClorDiSys.
- 11 humidity and temperature monitors (TempTale/Sensitech—Massachusetts, USA).
- Sterilization chambers (Steridox-100 vacuum pressure chambers, produced by ClorDiSys)

### Chlorine dioxide gas

ClO_2_ is a unique single-electron-transfer-oxidizing agent known as a stable free radical. ClO_2_ is microbiocidal and is highly soluble in water. Unlike chlorine, its reaction chemistry does not lead to the formation of chlorinated organic products (trihalomethane and chloramine). At use concentrations, ClO_2_ is not flammable, explosive, carcinogenic, or ozone-depleting. After a sterilization cycle, chlorine dioxide gas breaks down into constituent parts, chlorite, chlorate, and chloride, which have relatively low toxicities in humans and are not cytotoxic or mutagenic [5].

ClO_2_ is a gas above −40°C (−40°F) at sterilization concentrations. Gaseous ClO_2_ is generated with the ClorDiSys CSI CD Cartridge (US EPA label # 80802-1) and is registered with the U.S. Environmental Protection Agency as a sterilant [6]. The US-EPA label specifies that chlorine dioxide can be used for sterilization applications. Prefilled syringes and medical devices are typically sterilized in an ambient temperature vacuum sterilizer.

### Validation process

Validation is a standard part of every sterilization process and demonstrates its effectiveness and reliability. Validation of a sterilization load (for example, a medical device or combination product) using ClO_2_ sterilization falls under the guidance of ISO 14937:2009 [7].

Validation of a sterilization process can be accomplished in several ways. Every validation method must demonstrate that the natural bioburden on a load is killed to a sterility assurance level (SAL) of 10^−6^ during the process. The validation method used in this experiment was the overkill method.

The overkill method is a conservative approach to terminal sterilization that goes beyond the minimum requirements needed to kill the target microorganisms, resulting in a high level of sterility assurance. The method uses a standardized, highly resistant microbial spore, typically *Geobacillus stearothermophilus* or *Bacillus atrophaeus*, as a biological indicator (BI). Both are more challenging to kill than the natural bioburden found on the load to be sterilized [8]. In this project, *Geobacillus stearothermophilus* was used. After exposure to sterilization, BIs are incubated in a growth-promoting medium under specific conditions to check for the growth of viable spores. No growth (a negative result) indicates the sterilization cycle was effective. The overkill method uses changes in process variables and varying load quantities to establish a sterilization window of minimum and maximum levels of sterilization.

Choosing a sublethal dose of sterilant is often the first step when determining the hierarchy of exposure levels. Exposure levels range from sublethal to lethal (half-cycle) to overkill (full or production sterilization cycles). The sublethal cycle, purposefully set below the expected lethal limit, used suboptimal sterilization parameters to kill some but not all the spores on the BIs, in particular, the ePCD. This nonlethal level becomes the known lower limit for subsequent sterilization cycles. The purpose of the sublethal cycle is to confirm that the ePCD is the “worst case” when compared to the iPCD and the natural bioburden on the load. All subsequent sterilization cycles will incorporate process variables above the lower limit.

**Fig 1:**
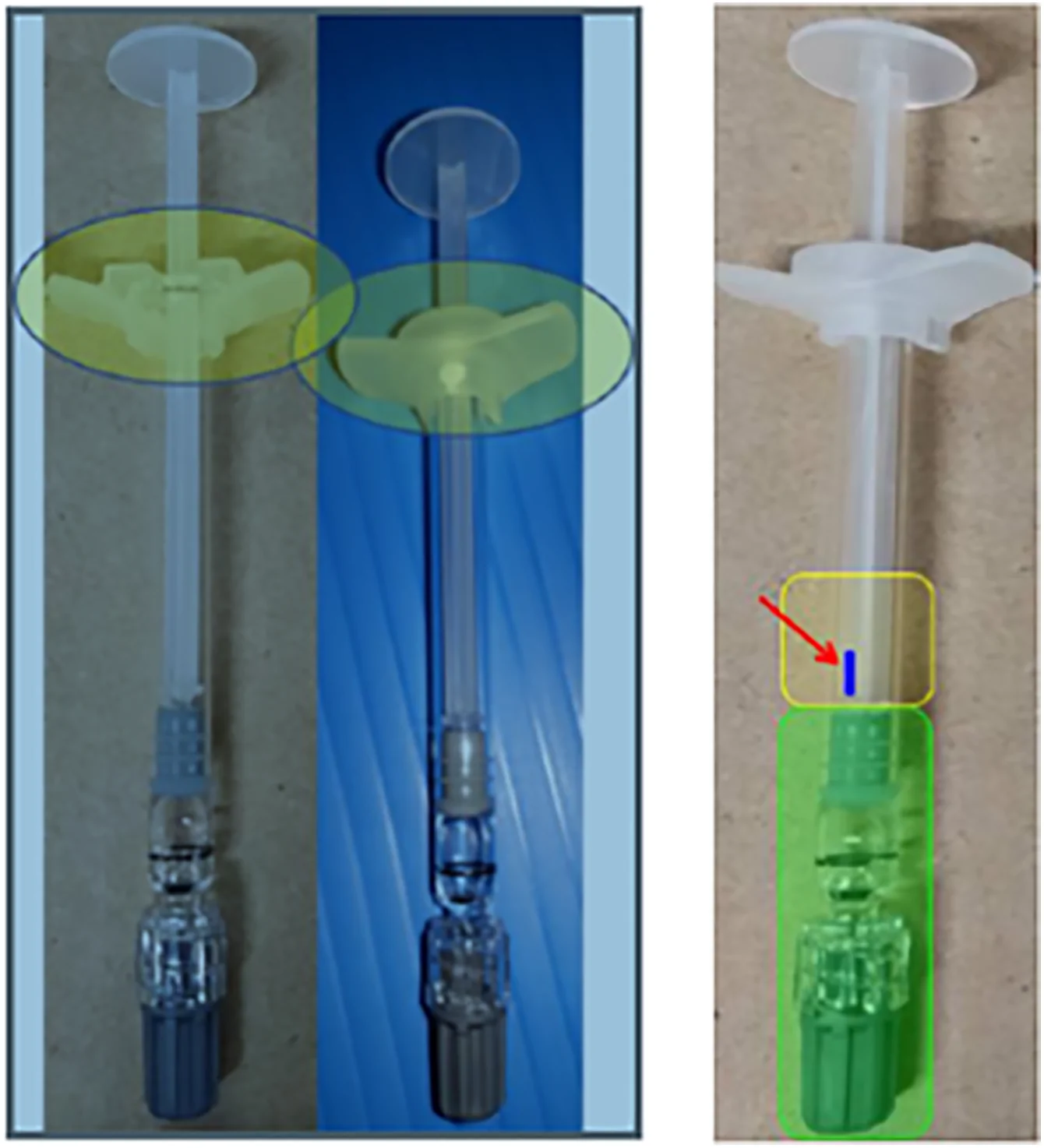
iPCD: biological indicator placed within the load (a prefilled syringe).

**Fig 2:**
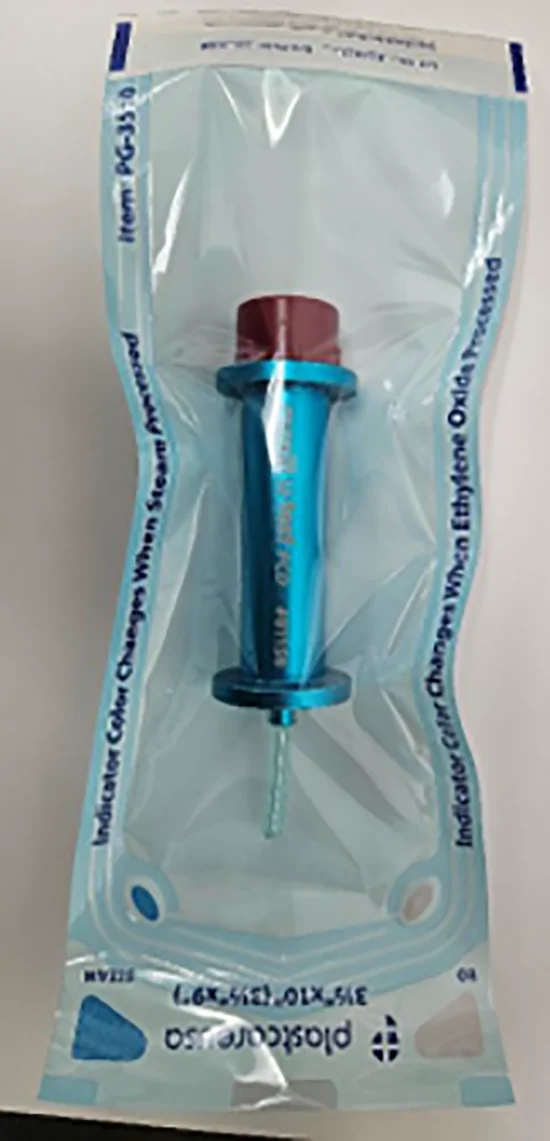
ePCD within sterilization pouch and placed alongside the load in the sterilization chamber.

**Fig 3:**
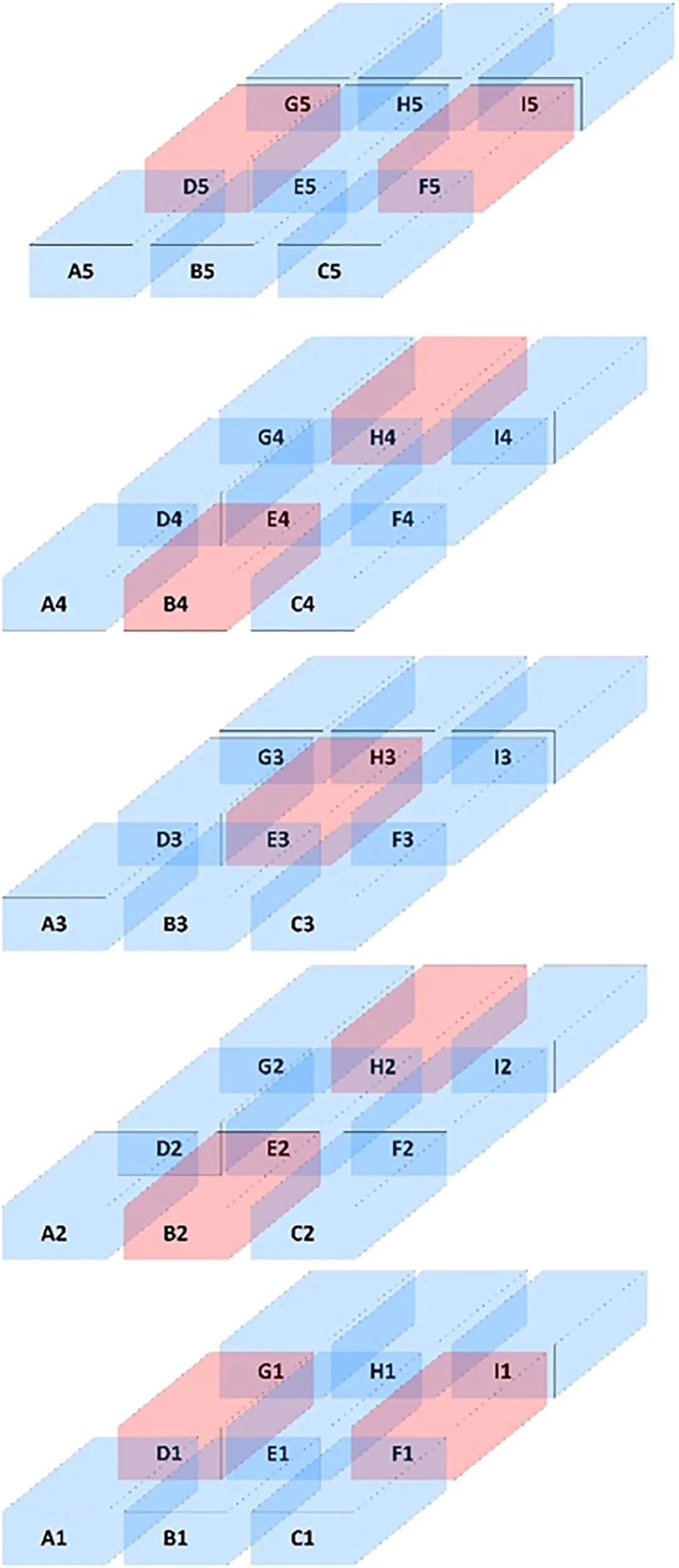
Product layout in the sterilization chamber. Red designates locations of iPCDs, ePCDs, and Dataloggers.

The validation process is accomplished by placing BIs within the load in the sterilization chamber (iPCDs) (Fig 1) and alongside the load (Fig 2), including difficult-to-reach locations (Fig 3). ePCDs are placed externally to the load and must be more challenging to kill than the natural bioburden and the iPCD within the load. The validation process continues by performing sterilization cycles at sublethal levels and then at lethal levels. After each cycle, the BIs are collected and processed to ensure spores are killed to the required SAL of 10^−6^. Ongoing production sterilization cycles are then set at the overkill level, which is double the dosage of chlorine dioxide as measured in ppm/hours, a half-cycle (lethal level).

The validation process used nineteen sterilization cycles to test a variety of load sizes and cycle parameters. Cycles included:

- One sublethal/fractional exposure cycle to establish the lethality hierarchy between the load’s natural bioburden and the iPCDs and ePCDs.
- Nine half-cycles at a variety of load sizes. The half-cycles used the cycle parameters needed to achieve lethality on the natural bioburden and a full kill on the iPCDs and ePCDs.
- Three full and half-cycles at minimal load
- One full and half-cycle at intermediate load

All loads were processed per protocol, except for the first load, which was a sublethal/fractional load. Each load was processed using both a half and a full sterilization cycle. Each half cycle exposed the product to approximately 50% of the dosage of chlorine dioxide as measured in ppm/hour. In addition to the half and full sterilization cycles, the loads consisted of maximum, minimum, and an intermediate quantity of PFS.

The validated ClO_2_ cycle was performed at ambient temperature, maintaining the cold-chain parameter by avoiding temperatures above 21°C. The average cycle lasted three hours and forty-five minutes, including preconditioning and aeration.

### Methods

#### Cold-chain parameters for PFS.

Restrictions on the handling of PFS included maintaining the product between 2°C and 8°C (35.6°F–46.6°F). These restrictions are typical of the handling requirements of cold-chain products.

#### Ingress and residual testing.

Ingress tests are designed to demonstrate the quantifiable presence or absence of chlorite, chloride, and chlorate via gas permeation or leakage within the cap, syringe barrel, plunger stopper, or liquid during ClO_2_ sterilization.

**Fig 4:**
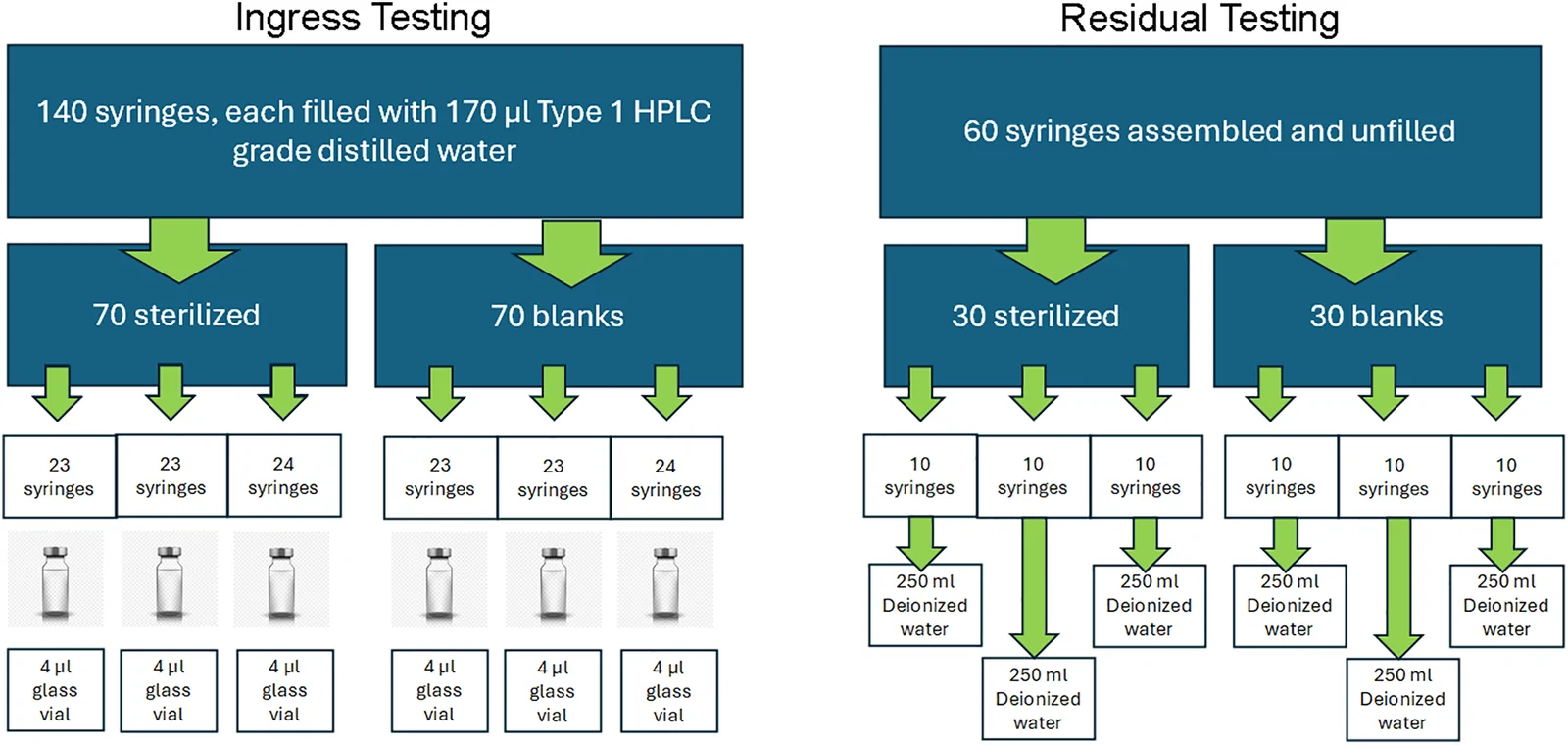
Ingress and residual testing methods.

For ingress testing, one hundred forty (140) syringe components were assembled and filled with type 1 high-performance liquid chromatography (HPLC) grade distilled water to the fill line of l70 μL (Fig 4). Seventy (70) filled syringes were used as blanks or controls, and the remaining 70 were exposed to chlorine dioxide sterilization.

Residual tests are designed to demonstrate the quantifiable presence or absence of chlorite, chloride, and chlorate on the outside of the PFS after ClO_2_ sterilization.

For residual testing, 60 syringe components were assembled (Fig 4). Thirty (30) assembled and unfilled syringes were used as blanks or controls, and the remaining 30 were exposed to ClO_2_ sterilization.

***Lab procedures*.** For ingress testing, filled samples were split into three groups of 23 syringes (with one group having 24 syringes). Syringe contents of each group were composited into 4 mL glass vials, which ran in duplicate for the three separate analytes (chlorite, chlorate, chloride). Unfilled samples were split into three groups of 10 syringes. Each group was composited in 250 mL of deionized water for two hours at room temperature. Solutions from each composite were run in duplicate for the three separate analytes.

For residual testing, sixty (60) syringe components were assembled. Thirty (30) assembled and unfilled syringes were used as blanks or controls, and the remaining 30 were exposed to ClO_2_ sterilization. To remove the residuals from the PFS, the PFS were submerged in a known amount of solution per ISO 11993-7 [9]. A verification test at Baron Labs demonstrated that almost all of the residue was dissolved by the solution prior to the ion chromatography testing concentrations using EPA Method 300.0, Determination of Inorganic Anions by Ion Chromatography [10].

#### Preparation summary.

ClorDiSys shipped sterilized samples at low temperature, surrounded by ice packs and in an insulated box, to Baron Labs, where they were stored at 4℃. Samples were extracted with the minimum volume of deionized water to ensure a proper extraction. A method blank of an unsterilized sample was prepared alongside the sterilized samples. After the samples were extracted, the solution was collected and analyzed by ion chromatography.

**Instrumentation used**

- Thermo Scientific Ion Chromatography
- Dionex IonPac™ AS22 Analytical Column 4 x 250 mm
- Dionex IonPac™ AG22 Guard Column 4 x 50 mm
- Dionex Electrolytically Regenerated Suppressor ERS 500 Carbonate 4 mm
- Chromeleon 7 Chromatography Data System
- Eluent:
  - 4.5mM Na2CO3/1.4 mM NaHCO3

***Sample analysis***. A standard containing Chloride, Chlorate, and Chlorite was run before and after the run. The sample solution was checked for any particulates in the extraction process. If particulates were present, the extract needed to be filtered. If particulates were not present, then the sample was run as is. The solution (1 mL) was then analyzed using Ion Chromatography per EPA method 300.0. The unsterilized samples were run first, followed by the sterilized samples. If there was a measured value above detection limits from the unsterilized samples, the measured values was subtracted from the sterilized samples. All samples were run in duplicate and averages recorded. After the data was collected and run, the customer request was calculated either per piece, surface area, or sample weight. A comprehensive test report was prepared and reviewed. The signed report was delivered to the customer by e-mail.

Each set of seventy syringes was then transferred to standard lab beakers for ion chromatography. Each set was averaged to get the final amount of chlorite, chlorate, and chloride in each sample.

***Lab procedure*.** The following steps were taken to follow the M710 Chlorine Dioxide Sterilization Feasibility Report.

The syringe was populated with spores on these syringe components: The body of the syringe was a Gerresheimer AG borosilicate glass type RTF® Syringe 1.0 ml (Luer Lock with TELC, inserted West 7025/65 grey closure, article number 663340012). The backstop that accompanies this syringe was also a Gerresheimer AG 1.0 ml Backstop (Polypropylene HP 371 P natural, article number 535200001). The plunger rod was a Becton-Dickinson (BO) “hypaktm Hypak™ plunger rod for backstop 1mll polypropylene natural large flange 20 (catalog/article number 47528108). The plunger was a West (Pharmaceutical Services Inc.) 1 ML Long NovaPure® Syringe Plunger 4023/50G.

The first location of the iPCD was the “back-side” of the backstop and the flange of the syringe body. This location was inoculated with a 6.4 mm/±¼ inch size quartz mat disc that is inoculated with 2: 1.0 x 1 as CFU of Geobacillus stearothermophilus. The second location of the iPCD was along the plunger rod and the glass syringe body, just above the position where it is screwed into the plunger itself. This location was inoculated with a 6.4 mm I ± ¼ inch size quartz mat disc that is inoculated with 2: 1.0 x 1 as CFU of Geobacillus stearothermophilus.

All ingress samples (Chloride, Chlorite, and Chlorate) were prepared in collaboration with contract laboratory Baron Analytical Laboratories LLC. According to the agreed plan (correct storage temperature, timings, etc.) between ClorDiSys and Baron Laboratory LLC. See Table 6 in Appendix 1.

The Tyvek lid was placed over the blister pack and sealed shut. Customer work instructions were used for proper loading procedure. The manufacturer instructions “OP-059 - CSI Steridox-VP Operation” were used for loading cycle parameters and running the desired cycle. The following OP-059 cycle parameters were used:

- Leak test set point (SP): 68 kPa
- Allowable pressure increase: 10 kPa
- Hold time: 1.0 minute
- Initial conditioning vacuum SP: 68.0 kPa
- CD charge vacuum SP: 68.0 kPa
- Exposure backfill pressure: 68.0 kPa
- Relative humidity (RH) pressure conditioning: OFF
- Control RH in exposure: OFF
- RH SP for Condition: 70% RH
- RH SP for Charge/Exposure: 0% RH
- Condition time: 60 minutes
- CD Setpoint: 5 mg/L
- PPM-hours:,8000 ppm-hours (full cycle)
- Control by ppm-hours toggle: ON

Once cycle parameters were confirmed, the cycle was started. The full cycle was run twice (in full to mimic the Single Lot Release approach). Between the cycles the iPCDs were refreshed since each cycle had its own set. ClorDiSys tested BIs for sterility per the internal operating procedure “OP-031, Dropping and Incubating Biological Indicators.” The following OP-031 parameters and were used and rules observed. The biological safety cabinet (BSC) certification sticker was confirmed current. The BSC blower was confirmed OFF and allowed to run for at least 10 minutes before commencing work. No personnel worked inside the BSC if there was an alarm condition. The sash was at the correct working height and the UV light was confirmed OFF. Readily accessible interior surfaces were disinfected with isopropyl alcohol or other accepted solution before use. The only items loaded were appropriate for the task being executed for the task. The cabinet was not overloaded. Each item was sprayed down before placement into the BSC. Personnel avoided disrupting airflow by minimizing movement (especially rapid movements) into and out of the BSC, and in areas near the BSC while working in the cabinet. The front grill and rear vents were not blocked by personnel or other materials. The cabinet was loaded at least four inches from the inside of the sash. After the work was completed, the BSC was wiped down with isopropyl alcohol or other accepted solution and the BSC was allowed to run for at least two to three minutes. After the completion of 7 days of incubation, results were documented. After the completion of both cycles (ingress sample will see two cycles). For a total dosage of 16,170 ppm-hours. Chloride, chlorite, and Chlorate ingress testing, results were documented.

#### Post-Sterilization expectations.

After a cycle, the gas rapidly degrades to its constituent parts. Therefore, no chlorine dioxide was expected to remain as a residual (whether as a liquid or a solid) on the outside of the PFS or the inside (cap, syringe barrel, plunger stopper, or liquid) during ingress testing. Instead, the byproducts of chlorite, chloride, and chlorate were tested for residual ingress on the PFS.

Baron Analytical Laboratories (Connecticut, USA) performed the residual and ingress tests per its validated ion chromatography process, which can detect chloride, chlorite, and chlorate levels to parts per million. Ion chromatography is an established and well-used testing method that separates ionized molecules based on differences in charge properties [11,12].

#### Evaluation criteria for chlorite and chlorate levels.

Currently, there is no harmonized standard or common specification for ClO_2_ residual levels on medical devices like PFS. However, toxicity guidelines are in place for the amount of chlorate and chlorite in drinking water (Table 1). According to the EPA, chlorite’s maximum contaminant level goal is 0.8 mg/L, and the maximum containment level for chlorite is 1.0 mg/L. [13] Although chlorate is not included in the EPA guidance, the World Health Organization has published a provisional guideline of 0.7 mg/L for chlorate amounts in drinking water. [14]

**Table 1:** EPA acceptable levels of chlorite and chlorate in drinking water.

Chemical Tested	Maximum Contaminant Level Goal (mg/L)	Maximum Contaminant Level (mg/L)
Chlorite	0.8	1.0
Chlorate	0.7	N/A

Drinking water guidelines serve as conservative parameters for measuring the toxicity of chlorite and chlorate on devices since many devices will not be ingested.

Although chloride results are included in the testing, the chloride levels are not included in the toxicity analysis. Chloride ions are abundant elements responsible for ionic homeostasis, osmotic pressure, and acid-based balance in the human body [15]. According to the World Health Organization, chloride concentrations in water exceeding 250 mg/L may affect the taste of the water [16]. Chloride is not considered toxic unless levels in the body are unbalanced [17].

## Results

### Residual testing results

A descriptive analysis of the residual testing results showed that although the amount of chlorite was slightly above the EPA contaminant level goal of 0.8 mg/L, the level was well below the maximum contaminant acceptable level of 1.0 mg/L. Table 2 lists a summary of results from residual testing, which demonstrates that residuals were within acceptable limits. Table 5 in Appendix 1 provides detailed results.

**Table 2:** Residual testing summary table.

	Sample Prefill Syringes (PFS)	
	PFS control (unsterilized)	PFS--Sterilized
**Average Chlorite**	0.74 mg/L	0.82 mg/L
**Maximum Contaminant Level Goal**	<0.8 mg/L	
**Maximum Contaminant Acceptable Level**	1.0 mg/L	
**Result (Pass/Fail)**		Pass
**Average Chloride**	1.36 mg/L	1.13 mg/L
**Acceptable Limit**	N/A	
**Result (Pass/Fail)**		Pass

PFS: prefilled syringe; avg: average.

### Ingress testing results

A descriptive analysis of the ingress testing results demonstrated that chlorite, chlorate, and chloride levels were below both the maximum contaminant level goal (0.8 mg/L) and the maximum contaminant level (1.0 mg/L) (Table 3).

**Table 3:** Ingress testing summary table.

	Sample	
**PFS control (unsterilized)**	**PFS--Sterilized**
**Chlorite (avg)**	0.78	0.71
**Maximum Contaminant Level Goal (mg/L)**		<0.8 mg/L
**Maximum Contaminant Acceptable Level (mg/L)**		1.0 mg/L
**Result (Pass/Fail)**		Pass
**Chloride (avg)**		1.06
**Maximum Contaminant Level Goal (mg/L)**		<0.8 mg/L
**Maximum Contaminant Level (mg/L)**		1.0 mg/L
**Result (Pass/Fail)**		Pass
**Chlorate (avg)**	N/A	0.37
**Maximum Contaminant Level Goal (mg/L)**		<0.7 mg/L
**Maximum Contaminant Level (mg/L)**		N/A
**Result (Pass/Fail)**		Pass

PFS: prefilled syringe; avg: average.

#### Sample analysis.

Samples 176458-1 and 176458-3 were split into three different composite groups, each made up of 23 syringes. The contents of the syringes were composited into three clean 4mL glass vials. The vials were then run in duplicate for the specified three analytes. Each run is tabulated in the table below (Table 3) and an average value is reported for all three composites. Samples 176458-2 and 176458-4 were split into three different composite groups; each group was made up of 10 syringes. Each composite of syringes was then extracted in exactly 250mL of deionized water for 2 hours at room temperature. The solution from each composite was then run in duplicate for the specified three analytes. Each run is tabulated below, with an average value reported for all three of the composites.

## Data analysis and statistical methods

Simple averages were used during the collection and analysis process. The data was not powered for formal statistical analysis.

### Discussion

#### Residue and ingress within acceptable low limits.

Residue testing demonstrated that the presence of chlorite, chloride, and chlorate was within acceptable limits as defined by the U.S. EPA [13]. Ingress testing demonstrated that any chlorite, chloride, and chlorate that had permeated or leaked with the cap, syringe barrel, plunger stopper, or liquid during sterilization were within acceptable limits.

Medical device manufacturers seek alternatives to EtO terminal sterilization, given the global concerns about its sustainability [18]. EtO terminal sterilization can also leave residues on medical devices. These residues include ethylene oxide (EtO), ethylene glycol (EG), and ethylene chlorohydrin (ECH), which take to time to “off-gas.” Off-gassing time adds to the total sterilization time for EtO sterilization [19]. Chlorine dioxide presents as a viable alternative to EtO sterilization, especially in cold-chain sterilization.

In this experiment, water served as a surrogate for the protein. The validated chlorine dioxide cycle was performed at ambient temperature, allowing the product to avoid experiencing temperatures over 21°C. The average cycle lasted three hours and forty-five minutes, including preconditioning and aeration. In comparison, the ClO_2_ sterilization process kept temperatures within the acceptable range for cold-chain sterilization so the drug would not degrade. Table 4 compares the EtO sterilization with the ClO_2_ sterilization process.

**Table 4:** Comparison: EtO vs. ClO_2_ sterilization process.

Step	Temperature range	Time range
**Ethylene Oxide Full Cycle**		
Preconditioning	38-49°C	18-24 hours
Conditioning	31-42°C	59-79 minutes
Gas Exposure	31-42°C	2.75-3 hours
Heated Aeration	38-55°C	12-18 hours
**Chlorine Dioxide Full Cycle**		
Preconditioning	21°C	12 minutes
Conditioning	20°C	60 minutes
Gas Exposure	21°C	2 hours
Aeration	20°C	30 minutes

#### Advantages of chlorine dioxide sterilization.

ClO_2_ is an effective choice as a terminal sterilant for cold chain sterilization needs. In addition to avoiding temperature spikes and minimizing TOR, ClO_2_ sterilization minimizes cycle length, suggesting that supply chain timing can also be shortened. ClO_2_ also provides the manufacturer with the capacity for in-house sterilization. The ClO_2_ sterilization process is also much simpler than other alternative sterilization modalities because of the ability to sterilize in unit cartons, with instructions for use (IFUs) and other packaging. This streamlines the process and avoids additional logistical complexities. ClO_2_ also eases environmental and health concerns compared to EtO and other sterilization methodologies [20,21].

#### Limitations.

This experiment describes ingress and residual testing on syringes prefilled with type 1 HPLC-grade distilled water, which may not be generalizable to all medications. Further testing is needed to determine if ClO_2_ sterilization affects different types of medications. Future experiments could use the same method to test ingress and residuals on other types of medical devices, such as inhalers or devices to treat corneal abrasions. While the sample size for testing was insufficient for a formal statistical analysis, the descriptive analysis showed promising results. Future experiments with larger sample sizes would enable more robust, generalizable conclusions.

## Conclusion

The sterilization of the PFS using ClO_2_ effectively maintained the required temperatures and kept residual and ingress chlorite, chloride, and chlorate within acceptable levels.

## Appendix 1—detailed testing results

### Residual testing detailed results

Table 5 provides detailed information for residual testing.

**Table 5:** Residual testing detail.

Sample ID	Testing data	Retention time	Amount (mg/L)	Peak area (UNITS)	Peak heights (UITS)
176458-2-1—Control/Blanks (Not exposed to chlorine dioxide gas for sterilization)					
Chlorite	5/26/22	3.83	0.76	63629	2622
Chloride	5/26/22	4.93	1.56	505280	21106
176458-2-2—Control/Blanks (Not exposed to chlorine dioxide gas for sterilization)					
Chlorite	5/26/22	3.82	0.70	53162	2265
Chloride	5/26/22	4.97	1.17	13324	510
176458-2-3—Control/Blanks (Not exposed to chlorine dioxide gas for sterilization)					
Chlorite	5/26/22	3.83	0.76	63069	2641
Chloride	5/26/22	4.95	1.35	432708	17057
176458-4-1—Exposed to chlorine dioxide gas for sterilization					
Chlorite	5/26/22	3.85	0.84	75791	3151
Chloride	5/26/22	4.92	1.09	341800	12649
176458-4-2—Exposed to chlorine dioxide gas for sterilization					
Chlorite	5/26/22	3.83	0.84	75342	3268
Chloride	5/26/22	4.92	1.46	469400	17848
176458-4-3—Exposed to chlorine dioxide gas for sterilization					
Chlorite	5/26/22	3.82	0.79	67934	3216
Chloride	5/26/22	4.87	0.83	249039	10782

### Ingress testing detailed results

Table 6 provides detailed information for ingress testing.

**Table 6:** Ingress testing detail.

Sample ID	Date	Retention Time	Amount	Peak Area	Peak Height
176458-1-1—Control/Blanks (Not exposed to chlorine dioxide gas for sterilization)					
Chlorite	5/26/22	3.77	0.88	80889	2873
Chloride	5/26/22	4.87	1.16	367014	18059
176458-1-2—Control/Blanks (Not exposed to chlorine dioxide gas for sterilization)					
Chlorite	5/26/22	3.75	0.63	43834	2173
Chloride	5/26/22	4.87	0.92	282764	13197
176458-1-3—Exposed to chlorine dioxide gas for sterilization					
Chlorite	5/26/22	3.80	0.84	75102	2392
Chloride	5/26/22	4.95	1.70	553621	22336
Chlorate	5/26/22	9.02	0.35	14775	678
176458-3-1—Exposed to chlorine dioxide gas for sterilization					
Chlorite	5/26/22	3.82	0.89	83050	2821
Chloride	5/26/22	4.95	1.49	480428	21350
Chlorate	5/26/22	9.02	0.40	20817	861
176458-3-2—Exposed to chlorine dioxide gas for sterilization					
Chlorite	5/26/22	3.85	0.87	79345	2679
Chloride	5/26/22	5.00	0.95	292015	13602
176458-3-3—Exposed to chlorine dioxide gas for sterilization					
Chlorite	5/26/22	3.85	0.36	10941	565
Chloride	5/26/22	4.98	0.75	222972	14227
Chlorate	5/26/22	9.07	0.39	19637	881

## Supporting information

S1 File IngressData. (PDF)

S2 File M710 ResidualsData. (PDF)
