# Supplementary material for: Chlorine dioxide gas sterilization of cold-chain or temperature-sensitive devices
Source: PLoS One. 2026 Aug 27;21(8):e0321345. doi: 10.1371/journal.pone.0321345 (PMC13521399; doi:10.1371/journal.pone.0321345)
Supplement: Supplementary file 1 [file pone.0321345.s001.pdf]

ClorDiSys

Steridox-VP Cycle Run Report (Page 1 of 3)

236 Run Number

Cycle Start Date: 05 / 24 / 22 End Date (MM/DD/YY): 05 / 24 / 22

User: CSI

Alarm Status: Cycle Successful No Abort Alarms Occurred

Cycle Parameters

[redacted]	Recipe Name
01	Loaded Recipe
68	Leak Test SP (K Pa)
10	Allow Press Inc (K Pa)
1.0	Hold Time (min)
68.0	Initial Vacuum SP (K Pa)
68.0	CD Charge Vacuum SP (K Pa)
68.0	Exposure Backfill Pressure (KPa)
Off	RH / Pressure Conditioning
Off	Control RH In Exposure
70	RH SP for Condition (%)
0	RH SP for Charge / Exposure (%)
60	Condition Time (min)
5.0	CD Charge Conc. (mg/L)
8000	PPM-Hrs
On	Control by PPM-Hrs

Cycle Run Step Data

	Time H : M : S	Temperature (Deg C)	RH (%)	Conc. (mg/L)	Pressure (KPa)
Cycle Start	09 : 35 : 28	24.4	49	0.0	100.1
At Vacuum Pull	09 : 37 : 39	24.5	44	0.0	67.2
Start Condition	09 : 56 : 14	24.9	70	0.0	68.9
Start Charge	10 : 56 : 22	25.3	70	0.0	67.9
Reading Before Backfill	11 : 05 : 12	25.3	69	5.2	73.8
Start Exposure	11 : 05 : 12	25.3	69	5.2	73.8
Start Aeration	15 : 19 : 21	23.8	58	5.2	87.2
Cycle Completed	15 : 46 : 06	23.9	58	0.0	100.1

Condition Phase

	Minimum	Maximum
Temperature (deg C)	24.8	25.4
RH (%)	65	73

Exposure Phase

	Minimum	Maximum
Concentration (mg/L)	5.0	5.4
Temperature (deg C)	23.7	25.4
RH (%)	58	69

Time & Consummable Usage

	H : M
Total Cycle Time	06 : 10.6
Final Dosage (PPM-Hrs)	8086
Chlorine PSI Used	98

	Remain	Used
Cartridge Minutes	996.8	16.3

Cartridge Expiration Date: 11 / 22

Cartridge Lot Number: 11032021C

Software Versions

HMI V 3.00 PLC V 3.00

Print V 1.01

Chamber Serial

SVP-05A-2021

Operator: [redacted] [redacted] Date: 05/25/2022 Mgmt Initials: [redacted]

Print Signature

ClorDiSys

Steridox-VP Config Parameters Report (Page 2 of 3)

236 Run Number

Cycle Start Date (MM / DD / YY) **05 / 24 / 22** User **CSI** [redacted] Recipe Name **01** Loaded Recipe

Chamber Serial # **SVP-05A-2021**

11	1 Leak Test Pause (sec)	95.0	23 High Vacuum SP (KPa)	2.0	43 Exposure CD Alm SP-X (mg/L)
150	2 Heat Up Time (sec)	10.0	24 Stop Pull Vacuum (P23-24)	300	44 Aeration Fault Timer (sec)
10	3 Condition Add to SP-X (%RH)	On	28 S90 Inj RH to SP	15	45 Cycle Hi RH SP (%RH)
15	4 Pause for RH Settle (sec)	On	29 S1040 Inj RH to SP	95	46 Hi Hi RH Alarm SP (%RH)
10	5 At Condition SP for X (sec)	On	30 S2020 Inj CD to SP	2.0	47 Hi CD Con SP+X (mg/L)
2.0	6 RH Burst Time (sec)	On	31 S2060 Inject CD to SP	15	48 Low Temp Alarm SP (Deg C)
60	8 Stm Inj Cool Down Time (sec)	On	32 S3020 Inject CD to SP	40	49 Hi Temperature Alarm SP (Deg C)
60	9 Heat Up Stm Inj if Cool (sec)	5.0	33 CD at SP for X (sec)	30	51 Vacuum Pull Fault (Min)
10	10 Ht Up Stm Inj is Not Cl (sec)	Off	34 Charge Exposure PR	30	52 Vacuum Break Fault (Min)
5	11 Gen PR Time (sec)	300	35 Ch Pressure Alarm Time (sec)	30.0	61 2nd Aeration Vacuum SP (S4080)
10	12 Charge Pause (sec)	1800	36 Hum Excessive Time (sec)	2.0	62 Add more RH SP-X
3.0	13 CD Charge Minimum Inject	5	37 Condition RH Alarm SP-X	5.0	63 Pause RH timer at SP-X
2.0	15 CD Expose Inject Time (sec)	2	38 Excessvie Cond Time Multiply	3.0	64 Keep RH on for X sec
25.0	17 Initial Aeratain Vacuum SP	0	39 Slow Charge Time X/Y	0.2	65 Adjust CD Exposure
0.5	19 CD Reduce SP (mg/L)	10	40 Slow Charge Concentration	0.0	66 Exposure at SP-X [Pause Timer
85.0	20 Aer Vac Break SP (KPa)	40	41 Excessive Charge Time	8	67 Vacuum Break cycles
95.0	21 S4120 Vac Brk SP (KPa)	2	42 Excessive Exposure Time		

Operator: [redacted] Print [redacted] Signature Date: 05/25/2022 Mgmt Initials: [redacted]

CD ClorDiSys

Steridox-VP Global Parameter Set Report (Page 3 of 3)

236 Run Number

Cycle Start Date (MM / DD / YY): 05 / 24 / 22 | User: CSI | [redacted] | Recipe Name: 01 Loaded Recipe | Chamber Serial #: SVP-05A-2021

300	60 Cartridge Maximum (Min)	On	85 Use CSI Steam Generator	A	100 Use CD A/B sensor
20	61 Gen Chlorine Flow Rate (LPM)	60.0	86 PT-3 Maximum Scale (PSI)	A	101 Use RH A/B sensor
1.0	62 Gen Pressure Relief SP (PSI)	10.0	87 Gen Pres Low / Loss Flt (PT3<X)(PSI)	A	102 Use TT A/B sensor
103.4	66 Vac Sen (PT-4A) Max (KPa)	17.0	88 CSI Steam Gen Heat Up Time (Min)	A	103 Use PT-4 A/B sensor
20	67 Reg Pressure Low Fault (PSI)	120	89 System Inactivity, Auto-Log Off (Min)	On	104 Use External Scrubber on Vac Pump
35	68 Reg Pressure Hi Fault (PSI)	On	90 Use Oil Vacuum Pump		
-40.0	69 Temp Lower Scale (Deg C)	5.0	91 RH Difference Alert (%RH)		
60.0	70 Temp Upper Scale (Deg C)	10.0	92 Temperature Difference Alert (Deg C)		
10	76 Gen Pressure Alarm (PSI)	10.0	93 Chamber Pressure Difference		
5	79 Cart Max Add (P60+X) Stop	0.5	94 CD Difference Alert (mg/L)		
50	80 Step 10 Vacuum SP (KPa)	0	95 Vacuum Break cycles		
80	81 Break Vacuum SP (KPa)	On	96 Use Automatic Doors		
30	82 Vacuum Pull SP (KPa)	103.4	97 Vac Sen (PT-4B) Max (KPa)		
8	83 Vacuum / Break Cycles	25.0	98 Automatic Door Gasket Deflate Time (sec)		
100	84 Chamber Volume (Cu Ft)	0.0	99 Vacuum Valve Delay Timer (sec)		

Note: Parameters 80-83 are used for chlorine and cartridge changeout

Operator: [redacted] Print [redacted] Signature Date: 05/25/2022 Mgmt Initials: [redacted]

ClorDiSys

Steridox-VP Cycle Run Report (Page 1 of 3)

237 Run Number

Cycle Start Date	End Date (MM/DD/YY)	User	Alarm Status
05 / 25 / 22	05 / 25 / 22	CSI	Cycle Successful No Abort Alarms Occurred

Cycle Parameters

[redacted]	Recipe Name
01	Loaded Recipe
68	Leak Test SP (K Pa)
10	Allow Press Inc (K Pa)
1.0	Hold Time (min)
68.0	Initial Vacuum SP (K Pa)
68.0	CD Charge Vacuum SP (K Pa)
68.0	Exposure Backfill Pressure (KPa)
Off	RH / Pressure Conditioning
Off	Control RH In Exposure
70	RH SP for Condition (%)
0	RH SP for Charge / Exposure (%)
60	Condition Time (min)
5.0	CD Charge Conc. (mg/L)
8000	PPM-Hrs
On	Control by PPM-Hrs

Cycle Run Step Data

	Time H : M : S	Temperature (Deg C)	RH (%)	Conc. (mg/L)	Pressure (KPa)
Cycle Start	08 : 35 : 48	22.1	38	0.0	100.5
At Vacuum Pull	08 : 37 : 59	22.1	30	0.0	67.1
Start Condition	09 : 07 : 40	23.4	70	0.0	69.8
Start Charge	10 : 08 : 44	24.6	68	0.0	67.9
Reading Before Backfill	10 : 15 : 44	24.6	69	5.3	72.9
Start Exposure	10 : 15 : 44	24.6	69	5.3	72.9
Start Aeration	14 : 30 : 43	24.1	59	5.2	84.3
Cycle Completed	14 : 57 : 28	24.1	58	0.0	100.3

Condition Phase

	Minimum	Maximum
Temperature (deg C)	23.4	24.6
RH (%)	65	72

Exposure Phase

	Minimum	Maximum
Concentration (mg/L)	5.0	5.5
Temperature (deg C)	23.8	24.7
RH (%)	59	69

Time & Consummable Usage

			Remain	Used
Total Cycle Time (H : M)	06 : 21.6	Cartridge Minutes	289.8	10.4
Final Dosage (PPM-Hrs)	8084	Cartridge Expiration Date	05 / 23	
Chlorine PSI Used	66	Cartridge Lot Number	04282022B	

Software Versions

HMI V 3.00 PLC V 3.00

Print V 1.01

Chamber Serial #

SVP-05A-2021

Operator: [redacted] [redacted] Date: 05/25/2022 Mgmt Initials: [redacted]

Print Signature

ClorDiSys

Steridox-VP Config Parameters Report (Page 2 of 3)

237 Run Number

Cycle Start Date (MM / DD / YY) 05 / 25 / 22 | User CSI | [redacted] | Recipe Name 01 Loaded Recipe | Chamber Serial # SVP-05A-2021

11	1 Leak Test Pause (sec)	95.0	23 High Vacuum SP (KPa)	2.0	43 Exposure CD Alm SP-X (mg/L)
150	2 Heat Up Time (sec)	10.0	24 Stop Pull Vacuum (P23-24)	300	44 Aeration Fault Timer (sec)
10	3 Condition Add to SP-X (%RH)	On	28 S90 Inj RH to SP	15	45 Cycle Hi RH SP (%RH)
15	4 Pause for RH Settle (sec)	On	29 S1040 Inj RH to SP	95	46 Hi Hi RH Alarm SP (%RH)
10	5 At Condition SP for X (sec)	On	30 S2020 Inj CD to SP	2.0	47 Hi CD Con SP+X (mg/L)
2.0	6 RH Burst Time (sec)	On	31 S2060 Inject CD to SP	15	48 Low Temp Alarm SP (Deg C)
60	8 Stm Inj Cool Down Time (sec)	On	32 S3020 Inject CD to SP	40	49 Hi Temperature Alarm SP (Deg C)
60	9 Heat Up Stm Inj if Cool (sec)	5.0	33 CD at SP for X (sec)	30	51 Vacuum Pull Fault (Min)
10	10 Ht Up Stm Inj is Not Cl (sec)	Off	34 Charge Exposure PR	30	52 Vacuum Break Fault (Min)
5	11 Gen PR Time (sec)	300	35 Ch Pressure Alarm Time (sec)	30.0	61 2nd Aeration Vacuum SP (S4080)
10	12 Charge Pause (sec)	1800	36 Hum Excessive Time (sec)	2.0	62 Add more RH SP-X
3.0	13 CD Charge Minimum Inject	5	37 Condition RH Alarm SP-X	5.0	63 Pause RH timer at SP-X
2.0	15 CD Expose Inject Time (sec)	2	38 Excessvie Cond Time Multiply	3.0	64 Keep RH on for X sec
25.0	17 Initial Aeratain Vacuum SP	0	39 Slow Charge Time X/Y	0.2	65 Adjust CD Exposure
0.5	19 CD Reduce SP (mg/L)	10	40 Slow Charge Concentration	0.0	66 Exposure at SP-X (Pause Timer
85.0	20 Aer Vac Break SP (KPa)	40	41 Excessive Charge Time	8	67 Vacuum Break cycles
95.0	21 S4120 Vac Brk SP (KPa)	2	42 Excessive Exposure Time		

Operator: [redacted] Print

[redacted] Signature

Date: 05/25/2022

Mgmt Initials: [redacted]

ClorDiSys

Steridox-VP Global Parameter Set Report (Page 3 of 3)

237 Run Number

Cycle Start Date (MM / DD / YY) 05 / 25 / 22 | User CSI | [redacted] | Recipe Name 01 Loaded Recipe | Chamber Serial # SVP-05A-2021

300	60 Cartridge Maximum (Min)	On	85 Use CSI Steam Generator	A	100 Use CD A/B sensor
20	61 Gen Chlorine Flow Rate (LPM)	60.0	86 PT-3 Maximum Scale (PSI)	A	101 Use RH A/B sensor
1.0	62 Gen Pressure Relief SP (PSI)	10.0	87 Gen Pres Low / Loss Flt (PT3<X) (PSI)	A	102 Use TT A/B sensor
103.4	66 Vac Sen (PT-4A) Max (KPa)	17.0	88 CSI Steam Gen Heat Up Time (Min)	A	103 Use PT-4 A/B sensor
20	67 Reg Pressure Low Fault (PSI)	120	89 System Inactivity, Auto-Log Off (Min)	On	104 Use External Scrubber on Vac Pump
35	68 Reg Pressure Hi Fault (PSI)	On	90 Use Oil Vacuum Pump		
-40.0	69 Temp Lower Scale (Deg C)	5.0	91 RH Difference Alert (%RH)		
60.0	70 Temp Upper Scale (Deg C)	10.0	92 Temperature Difference Alert (Deg C)		
10	76 Gen Pressure Alarm (PSI)	10.0	93 Chamber Pressure Difference		
5	79 Cart Max Add (P60+X) Stop	0.5	94 CD Difference Alert (mg/L)		
50	80 Step 10 Vacuum SP (KPa)	0	95 Vacuum Break cycles		
80	81 Break Vacuum SP (KPa)	On	96 Use Automatic Doors		
30	82 Vacuum Pull SP (KPa)	103.4	97 Vac Sen (PT-4B) Max (KPa)		
8	83 Vacuum / Break Cycles	25.0	98 Automatic Door Gasket Deflate Time (sec)		
100	84 Chamber Volume (Cu Ft)	0.0	99 Vacuum Valve Delay Timer (sec)		

Note: Parameters 80-83 are used for chlorine and cartridge changeout

Operator: [redacted] Print

[redacted] Signature

Date: 05/25/2022

Mgmt Initials: [redacted]

ATTACHMENT 4

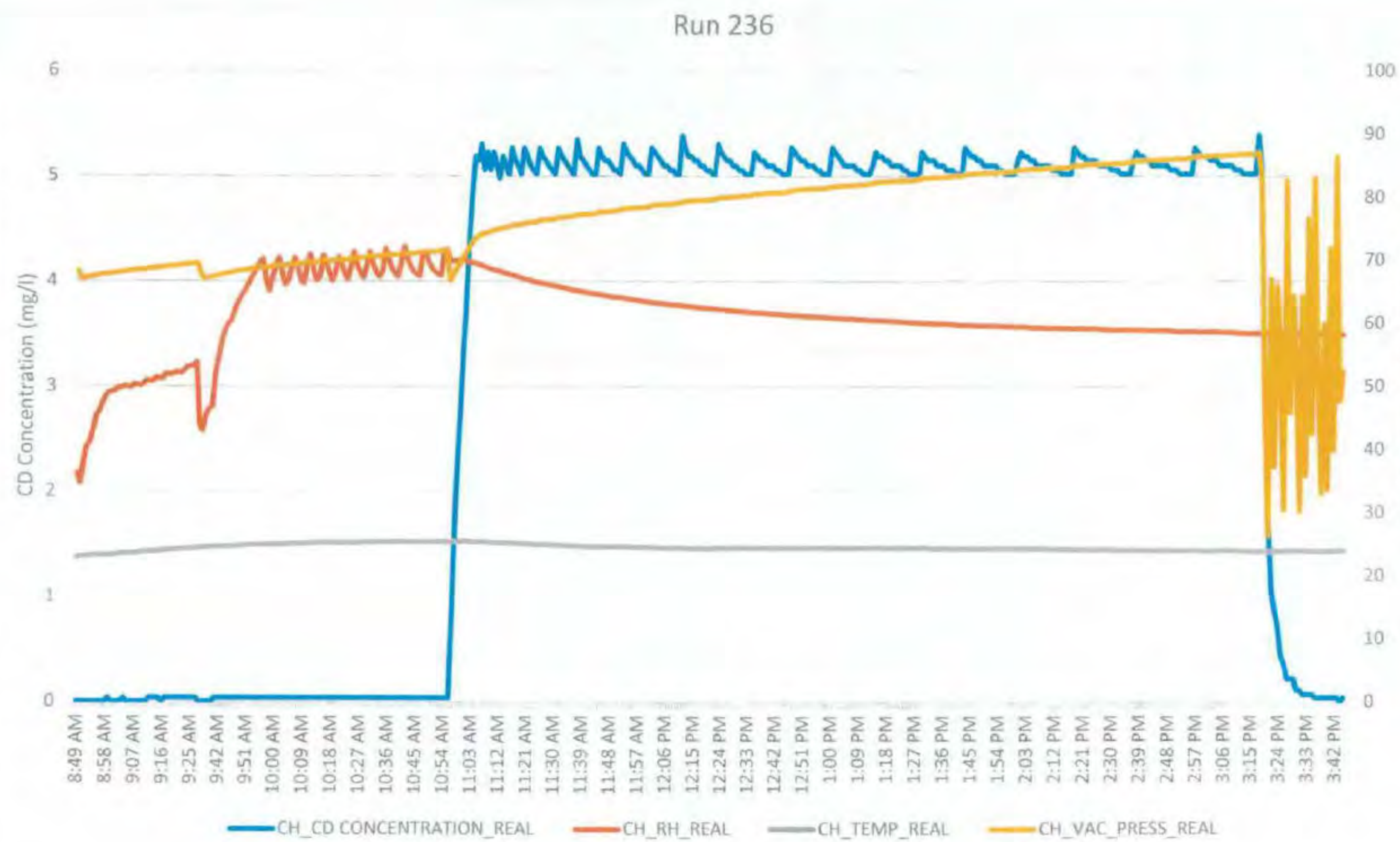

Run 236
CD Concentration (mg/l)
RH, Temperatre (˚F), Pressure
CH_CD CONCENTRATION_REAL
CH_RH_REAL
CH_TEMP_REAL
CH_VAC_PRESS_REAL

Date	CH_CD CONCENTRATION_REAL	CH_RH_REAL	CH_TEMP_REAL	CH_VAC_PRESS_REAL	PPM-HRS_	RUNNUMBER_DOUBLE
5/24/2022 9:36	0	43.59999847	24.29999924	67.69999695	0	236
5/24/2022 9:37	0	43	24.5	67.09999847	0	236
5/24/2022 9:38	0	45.5	24.5	67.19999695	0	236
5/24/2022 9:39	0	46.40000153	24.5	67.30000305	0	236
5/24/2022 9:40	0	46.79999924	24.60000038	67.40000153	0	236
5/24/2022 9:41	0.039999999	52	24.60000038	67.59999847	0	236
5/24/2022 9:42	0.039999999	54.79999924	24.60000038	67.69999695	0	236
5/24/2022 9:43	0.039999999	57.09999847	24.60000038	67.80000305	0	236
5/24/2022 9:44	0.039999999	58.90000153	24.60000038	67.90000153	0	236
5/24/2022 9:45	0.039999999	60	24.60000038	68	0	236
5/24/2022 9:46	0.039999999	60.5	24.60000038	68.09999847	0	236
5/24/2022 9:47	0.039999999	62	24.60000038	68.19999695	0	236
5/24/2022 9:48	0.039999999	63.20000076	24.60000038	68.30000305	0	236
5/24/2022 9:49	0.039999999	64.19999695	24.70000076	68.40000153	0	236
5/24/2022 9:50	0.039999999	65	24.70000076	68.40000153	0	236
5/24/2022 9:51	0.039999999	65.90000153	24.79999924	68.5	0	236
5/24/2022 9:52	0.039999999	66.90000153	24.79999924	68.59999847	0	236
5/24/2022 9:53	0.039999999	67.80000305	24.79999924	68.69999695	0	236
5/24/2022 9:54	0.039999999	68.69999695	24.89999962	68.80000305	0	236
5/24/2022 9:55	0.039999999	69.69999695	24.89999962	68.90000153	0	236
5/24/2022 9:56	0.039999999	70.19999695	24.89999962	68.80000305	0	236
5/24/2022 9:57	0.039999999	67	24.89999962	68.80000305	0	236
5/24/2022 9:58	0.039999999	65.09999847	24.89999962	68.90000153	0	236
5/24/2022 9:59	0.039999999	67.30000305	24.89999962	69	0	236
5/24/2022 10:00	0.039999999	69.19999695	24.89999962	69.19999695	0	236
5/24/2022 10:01	0.039999999	70.40000153	24.89999962	69.19999695	0	236
5/24/2022 10:02	0.039999999	68.19999695	24.89999962	69.09999847	0	236
5/24/2022 10:03	0.039999999	66.09999847	24.89999962	69.19999695	0	236
5/24/2022 10:04	0.039999999	66.59999847	25	69.30000305	0	236
5/24/2022 10:05	0.039999999	68.90000153	25	69.40000153	0	236
5/24/2022 10:06	0.039999999	70.40000153	25	69.40000153	0	236
5/24/2022 10:07	0.039999999	69	25	69.40000153	0	236
5/24/2022 10:08	0.039999999	66.80000305	25	69.40000153	0	236

5/24/2022 10:09	0.039999999	66.30000305	25.10000038	69.5	0	236
5/24/2022 10:10	0.039999999	69.5	25.10000038	69.69999695	0	236
5/24/2022 10:11	0.039999999	70.90000153	25.10000038	69.69999695	0	236
5/24/2022 10:12	0.039999999	68.40000153	25.10000038	69.69999695	0	236
5/24/2022 10:13	0.039999999	66.80000305	25.20000076	69.69999695	0	236
5/24/2022 10:14	0.039999999	67.30000305	25.20000076	69.80000305	0	236
5/24/2022 10:15	0.039999999	70.69999695	25.20000076	69.90000153	0	236
5/24/2022 10:16	0.039999999	69.90000153	25.20000076	69.90000153	0	236
5/24/2022 10:17	0.039999999	67.90000153	25.20000076	69.90000153	0	236
5/24/2022 10:18	0.039999999	66.69999695	25.20000076	69.90000153	0	236
5/24/2022 10:19	0.039999999	68.19999695	25.20000076	70.09999847	0	236
5/24/2022 10:20	0.039999999	70.59999847	25.20000076	70.09999847	0	236
5/24/2022 10:21	0.039999999	69.5	25.20000076	70.09999847	0	236
5/24/2022 10:22	0.039999999	67.80000305	25.20000076	70.09999847	0	236
5/24/2022 10:23	0.039999999	66.90000153	25.20000076	70.19999695	0	236
5/24/2022 10:24	0.039999999	69.30000305	25.20000076	70.30000305	0	236
5/24/2022 10:25	0.039999999	71.40000153	25.20000076	70.30000305	0	236
5/24/2022 10:26	0.039999999	69.30000305	25.20000076	70.30000305	0	236
5/24/2022 10:27	0.039999999	68	25.20000076	70.40000153	0	236
5/24/2022 10:28	0.039999999	67.19999695	25.20000076	70.40000153	0	236
5/24/2022 10:29	0.039999999	69.19999695	25.29999924	70.59999847	0	236
5/24/2022 10:30	0.039999999	71.40000153	25.29999924	70.59999847	0	236
5/24/2022 10:31	0.039999999	69.5	25.20000076	70.5	0	236
5/24/2022 10:32	0.039999999	68.19999695	25.29999924	70.59999847	0	236
5/24/2022 10:33	0.039999999	67.40000153	25.29999924	70.59999847	0	236
5/24/2022 10:34	0.039999999	68.30000305	25.29999924	70.80000305	0	236
5/24/2022 10:35	0.039999999	71.90000153	25.29999924	70.80000305	0	236
5/24/2022 10:36	0.039999999	70.30000305	25.29999924	70.80000305	0	236
5/24/2022 10:37	0.039999999	68.80000305	25.29999924	70.80000305	0	236
5/24/2022 10:38	0.039999999	68	25.29999924	70.90000153	0	236
5/24/2022 10:39	0.039999999	67.40000153	25.29999924	70.90000153	0	236
5/24/2022 10:40	0.039999999	69.59999847	25.39999962	71.09999847	0	236
5/24/2022 10:41	0.039999999	72.30000305	25.29999924	71.09999847	0	236
5/24/2022 10:42	0.039999999	70.40000153	25.29999924	71.09999847	0	236

5/24/2022 11:17	5.150000095	67.59999847	25.20000076	75.59999847	560.0139	236
5/24/2022 11:18	5.099999905	67.40000153	25.10000038	75.69999695	590.7839	236
5/24/2022 11:19	5.019999981	67.30000305	25.10000038	75.69999695	621.0713	236
5/24/2022 11:20	5.269999981	67.09999847	25	76	653.3496	236
5/24/2022 11:21	5.190000057	67	25	76	684.6626	236
5/24/2022 11:22	5.099999905	66.90000153	25	76.09999847	715.4326	236
5/24/2022 11:23	5.059999943	66.80000305	25	76.09999847	745.9613	236
5/24/2022 11:24	5.019999981	66.59999847	24.89999962	76.09999847	776.2487	236
5/24/2022 11:25	5.269999981	66.5	24.89999962	76.40000153	808.0443	236
5/24/2022 11:26	5.190000057	66.40000153	24.89999962	76.40000153	839.3573	236
5/24/2022 11:27	5.150000095	66.30000305	24.89999962	76.5	870.429	236
5/24/2022 11:28	5.099999905	66.19999695	24.89999962	76.5	901.199	236
5/24/2022 11:29	5.059999943	66.09999847	24.89999962	76.5	931.7277	236
5/24/2022 11:30	5.019999981	66	24.89999962	76.59999847	962.015	236
5/24/2022 11:31	5.269999981	65.90000153	24.79999924	76.90000153	993.8107	236
5/24/2022 11:32	5.190000057	65.80000305	24.79999924	76.90000153	1025.124	236
5/24/2022 11:33	5.150000095	65.69999695	24.79999924	77	1055.894	236
5/24/2022 11:34	5.099999905	65.59999847	24.79999924	77	1086.664	236
5/24/2022 11:35	5.059999943	65.5	24.70000076	77	1117.192	236
5/24/2022 11:36	5.019999981	65.30000305	24.70000076	77	1147.48	236
5/24/2022 11:37	5.309999943	65.30000305	24.70000076	77.30000305	1179.034	236
5/24/2022 11:38	5.190000057	65.19999695	24.60000038	77.30000305	1210.347	236
5/24/2022 11:39	5.150000095	65.09999847	24.60000038	77.30000305	1241.419	236
5/24/2022 11:40	5.099999905	65	24.60000038	77.40000153	1272.189	236
5/24/2022 11:41	5.059999943	64.90000153	24.60000038	77.40000153	1302.717	236
5/24/2022 11:42	5.019999981	64.80000305	24.60000038	77.40000153	1333.005	236
5/24/2022 11:43	5.019999981	64.69999695	24.60000038	77.5	1363.292	236
5/24/2022 11:44	5.269999981	64.69999695	24.60000038	77.80000305	1395.088	236
5/24/2022 11:45	5.190000057	64.59999847	24.60000038	77.80000305	1426.401	236
5/24/2022 11:46	5.150000095	64.5	24.60000038	77.80000305	1457.472	236
5/24/2022 11:47	5.099999905	64.40000153	24.60000038	77.90000153	1488.544	236
5/24/2022 11:48	5.099999905	64.30000305	24.60000038	77.90000153	1519.314	236
5/24/2022 11:49	5.059999943	64.19999695	24.60000038	77.90000153	1549.843	236
5/24/2022 11:50	5.059999943	64.19999695	24.60000038	77.90000153	1580.371	236

5/24/2022 11:51	5.019999981	64.09999847	24.60000038	78	1610.659	236
5/24/2022 11:52	5.309999943	64.09999847	24.60000038	78.30000305	1642.454	236
5/24/2022 11:53	5.230000019	64	24.60000038	78.30000305	1674.009	236
5/24/2022 11:54	5.190000057	63.90000153	24.5	78.30000305	1705.322	236
5/24/2022 11:55	5.150000095	63.79999924	24.5	78.40000153	1736.393	236
5/24/2022 11:56	5.099999905	63.79999924	24.5	78.40000153	1767.163	236
5/24/2022 11:57	5.099999905	63.70000076	24.5	78.40000153	1797.933	236
5/24/2022 11:58	5.059999943	63.59999847	24.5	78.5	1828.462	236
5/24/2022 11:59	5.059999943	63.5	24.5	78.5	1858.991	236
5/24/2022 12:00	5.019999981	63.5	24.5	78.59999847	1889.278	236
5/24/2022 12:01	5.269999981	63.40000153	24.5	78.80000305	1920.833	236
5/24/2022 12:02	5.230000019	63.29999924	24.39999962	78.80000305	1952.387	236
5/24/2022 12:03	5.190000057	63.29999924	24.39999962	78.90000153	1983.7	236
5/24/2022 12:04	5.150000095	63.20000076	24.39999962	78.90000153	2014.771	236
5/24/2022 12:05	5.150000095	63.20000076	24.39999962	78.90000153	2045.542	236
5/24/2022 12:06	5.099999905	63.09999847	24.39999962	79	2076.312	236
5/24/2022 12:07	5.059999943	63.09999847	24.39999962	79	2107.082	236
5/24/2022 12:08	5.059999943	63	24.39999962	79	2137.61	236
5/24/2022 12:09	5.019999981	63	24.39999962	79.09999847	2167.897	236
5/24/2022 12:10	5.019999981	62.90000153	24.39999962	79.09999847	2198.185	236
5/24/2022 12:11	5.389999866	62.90000153	24.39999962	79.40000153	2229.98	236
5/24/2022 12:12	5.269999981	62.79999924	24.39999962	79.40000153	2261.776	236
5/24/2022 12:13	5.190000057	62.79999924	24.29999924	79.5	2293.331	236
5/24/2022 12:14	5.190000057	62.70000076	24.39999962	79.5	2324.644	236
5/24/2022 12:15	5.150000095	62.70000076	24.39999962	79.5	2355.715	236
5/24/2022 12:16	5.099999905	62.70000076	24.39999962	79.59999847	2386.485	236
5/24/2022 12:17	5.099999905	62.59999847	24.29999924	79.59999847	2417.255	236
5/24/2022 12:18	5.099999905	62.5	24.39999962	79.59999847	2448.025	236
5/24/2022 12:19	5.059999943	62.5	24.29999924	79.59999847	2478.554	236
5/24/2022 12:20	5.059999943	62.5	24.39999962	79.69999695	2509.083	236
5/24/2022 12:21	5.019999981	62.40000153	24.29999924	79.69999695	2539.37	236
5/24/2022 12:22	5.019999981	62.29999924	24.39999962	79.69999695	2569.657	236
5/24/2022 12:23	5.309999943	62.29999924	24.29999924	80	2602.177	236
5/24/2022 12:24	5.230000019	62.29999924	24.39999962	80	2633.731	236

5/24/2022 12:25	5.190000057	62.20000076	24.39999962	80.09999847	2665.044	236
5/24/2022 12:26	5.150000095	62.20000076	24.29999924	80.09999847	2696.116	236
5/24/2022 12:27	5.099999905	62.09999847	24.39999962	80.09999847	2727.188	236
5/24/2022 12:28	5.099999905	62.09999847	24.39999962	80.09999847	2757.958	236
5/24/2022 12:29	5.099999905	62.09999847	24.39999962	80.19999695	2788.728	236
5/24/2022 12:30	5.059999943	62	24.39999962	80.19999695	2819.257	236
5/24/2022 12:31	5.059999943	62	24.39999962	80.19999695	2849.785	236
5/24/2022 12:32	5.019999981	61.90000153	24.39999962	80.30000305	2880.073	236
5/24/2022 12:33	5.019999981	61.90000153	24.39999962	80.30000305	2910.36	236
5/24/2022 12:34	5.019999981	61.79999924	24.39999962	80.30000305	2940.647	236
5/24/2022 12:35	5.230000019	61.79999924	24.39999962	80.59999847	2972.443	236
5/24/2022 12:36	5.190000057	61.79999924	24.39999962	80.59999847	3003.756	236
5/24/2022 12:37	5.150000095	61.70000076	24.39999962	80.69999695	3035.069	236
5/24/2022 12:38	5.150000095	61.70000076	24.39999962	80.69999695	3066.141	236
5/24/2022 12:39	5.099999905	61.70000076	24.39999962	80.69999695	3097.212	236
5/24/2022 12:40	5.099999905	61.59999847	24.39999962	80.80000305	3127.982	236
5/24/2022 12:41	5.099999905	61.59999847	24.39999962	80.80000305	3158.752	236
5/24/2022 12:42	5.059999943	61.59999847	24.39999962	80.80000305	3189.281	236
5/24/2022 12:43	5.059999943	61.5	24.39999962	80.90000153	3219.81	236
5/24/2022 12:44	5.019999981	61.5	24.39999962	80.90000153	3250.097	236
5/24/2022 12:45	5.019999981	61.40000153	24.39999962	80.90000153	3280.384	236
5/24/2022 12:46	5.019999981	61.40000153	24.39999962	80.90000153	3310.672	236
5/24/2022 12:47	5.269999981	61.29999924	24.39999962	81.19999695	3342.467	236
5/24/2022 12:48	5.230000019	61.40000153	24.39999962	81.19999695	3374.022	236
5/24/2022 12:49	5.190000057	61.29999924	24.39999962	81.30000305	3405.335	236
5/24/2022 12:50	5.150000095	61.29999924	24.39999962	81.30000305	3436.406	236
5/24/2022 12:51	5.150000095	61.20000076	24.39999962	81.30000305	3467.478	236
5/24/2022 12:52	5.099999905	61.20000076	24.39999962	81.40000153	3498.248	236
5/24/2022 12:53	5.099999905	61.20000076	24.39999962	81.40000153	3529.018	236
5/24/2022 12:54	5.099999905	61.09999847	24.39999962	81.40000153	3559.788	236
5/24/2022 12:55	5.059999943	61.09999847	24.39999962	81.40000153	3590.317	236
5/24/2022 12:56	5.059999943	61.09999847	24.39999962	81.5	3620.845	236
5/24/2022 12:57	5.019999981	61.09999847	24.39999962	81.5	3651.374	236
5/24/2022 12:58	5.019999981	61	24.39999962	81.5	3681.661	236

5/24/2022 12:59	5.019999981	61	24.39999962	81.59999847	3711.949	236
5/24/2022 13:00	5.269999981	61	24.39999962	81.80000305	3744.227	236
5/24/2022 13:01	5.230000019	61	24.39999962	81.80000305	3775.781	236
5/24/2022 13:02	5.190000057	60.90000153	24.39999962	81.90000153	3807.094	236
5/24/2022 13:03	5.150000095	60.90000153	24.39999962	81.90000153	3838.166	236
5/24/2022 13:04	5.099999905	60.79999924	24.39999962	81.90000153	3869.238	236
5/24/2022 13:05	5.099999905	60.79999924	24.39999962	81.90000153	3900.008	236
5/24/2022 13:06	5.099999905	60.79999924	24.39999962	82	3930.778	236
5/24/2022 13:07	5.099999905	60.79999924	24.39999962	82	3961.548	236
5/24/2022 13:08	5.059999943	60.70000076	24.39999962	82	3992.077	236
5/24/2022 13:09	5.059999943	60.70000076	24.39999962	82.09999847	4022.605	236
5/24/2022 13:10	5.019999981	60.70000076	24.39999962	82.09999847	4052.893	236
5/24/2022 13:11	5.019999981	60.70000076	24.39999962	82.09999847	4083.18	236
5/24/2022 13:12	5.019999981	60.59999847	24.39999962	82.19999695	4113.467	236
5/24/2022 13:13	5.099999905	60.59999847	24.39999962	82.30000305	4143.754	236
5/24/2022 13:14	5.230000019	60.59999847	24.39999962	82.40000153	4175.309	236
5/24/2022 13:15	5.190000057	60.5	24.5	82.5	4206.622	236
5/24/2022 13:16	5.190000057	60.5	24.39999962	82.5	4237.935	236
5/24/2022 13:17	5.150000095	60.5	24.39999962	82.5	4269.006	236
5/24/2022 13:18	5.150000095	60.40000153	24.39999962	82.59999847	4300.078	236
5/24/2022 13:19	5.099999905	60.40000153	24.39999962	82.59999847	4330.848	236
5/24/2022 13:20	5.099999905	60.40000153	24.5	82.59999847	4361.618	236
5/24/2022 13:21	5.099999905	60.40000153	24.39999962	82.59999847	4392.388	236
5/24/2022 13:22	5.099999905	60.29999924	24.39999962	82.69999695	4423.158	236
5/24/2022 13:23	5.059999943	60.29999924	24.39999962	82.69999695	4453.687	236
5/24/2022 13:24	5.059999943	60.29999924	24.39999962	82.69999695	4484.216	236
5/24/2022 13:25	5.019999981	60.20000076	24.39999962	82.69999695	4514.503	236
5/24/2022 13:26	5.019999981	60.20000076	24.39999962	82.69999695	4544.79	236
5/24/2022 13:27	5.019999981	60.20000076	24.39999962	82.80000305	4575.077	236
5/24/2022 13:28	5.019999981	60.09999847	24.39999962	82.80000305	4605.364	236
5/24/2022 13:29	5.230000019	60.20000076	24.39999962	83.09999847	4636.918	236
5/24/2022 13:30	5.190000057	60.09999847	24.39999962	83.09999847	4668.231	236
5/24/2022 13:31	5.150000095	60.09999847	24.39999962	83.09999847	4699.303	236
5/24/2022 13:32	5.150000095	60.09999847	24.39999962	83.09999847	4730.375	236

5/24/2022 13:33	5.099999905	60	24.39999962	83.09999847	4761.447	236
5/24/2022 13:34	5.099999905	60	24.29999924	83.19999695	4792.217	236
5/24/2022 13:35	5.099999905	60	24.29999924	83.30000305	4822.987	236
5/24/2022 13:36	5.099999905	60	24.39999962	83.30000305	4853.757	236
5/24/2022 13:37	5.059999943	60	24.29999924	83.30000305	4884.286	236
5/24/2022 13:38	5.059999943	60	24.29999924	83.30000305	4914.814	236
5/24/2022 13:39	5.059999943	59.90000153	24.29999924	83.30000305	4945.343	236
5/24/2022 13:40	5.019999981	59.90000153	24.29999924	83.40000153	4975.63	236
5/24/2022 13:41	5.019999981	59.90000153	24.29999924	83.40000153	5005.917	236
5/24/2022 13:42	5.019999981	59.90000153	24.29999924	83.40000153	5036.205	236
5/24/2022 13:43	5.269999981	59.79999924	24.29999924	83.69999695	5067.518	236
5/24/2022 13:44	5.230000019	59.79999924	24.29999924	83.69999695	5099.072	236
5/24/2022 13:45	5.190000057	59.79999924	24.29999924	83.69999695	5130.385	236
5/24/2022 13:46	5.190000057	59.70000076	24.29999924	83.80000305	5161.698	236
5/24/2022 13:47	5.150000095	59.70000076	24.29999924	83.80000305	5192.77	236
5/24/2022 13:48	5.150000095	59.70000076	24.29999924	83.80000305	5223.841	236
5/24/2022 13:49	5.150000095	59.70000076	24.29999924	83.90000153	5254.913	236
5/24/2022 13:50	5.099999905	59.70000076	24.29999924	83.90000153	5285.683	236
5/24/2022 13:51	5.099999905	59.70000076	24.29999924	83.90000153	5316.453	236
5/24/2022 13:52	5.099999905	59.59999847	24.29999924	83.90000153	5347.223	236
5/24/2022 13:53	5.099999905	59.59999847	24.29999924	84	5377.993	236
5/24/2022 13:54	5.059999943	59.59999847	24.29999924	84	5408.522	236
5/24/2022 13:55	5.059999943	59.59999847	24.29999924	84	5439.051	236
5/24/2022 13:56	5.059999943	59.59999847	24.29999924	84	5469.58	236
5/24/2022 13:57	5.019999981	59.5	24.29999924	84.09999847	5499.867	236
5/24/2022 13:58	5.019999981	59.5	24.29999924	84.09999847	5530.154	236
5/24/2022 13:59	5.019999981	59.5	24.29999924	84.09999847	5560.441	236
5/24/2022 14:00	5.150000095	59.5	24.29999924	84.30000305	5590.728	236
5/24/2022 14:01	5.230000019	59.5	24.29999924	84.40000153	5622.282	236
5/24/2022 14:02	5.190000057	59.5	24.29999924	84.40000153	5653.595	236
5/24/2022 14:03	5.190000057	59.5	24.29999924	84.40000153	5684.908	236
5/24/2022 14:04	5.150000095	59.40000153	24.29999924	84.5	5715.98	236
5/24/2022 14:05	5.150000095	59.40000153	24.29999924	84.5	5747.052	236
5/24/2022 14:06	5.150000095	59.40000153	24.29999924	84.5	5778.124	236

5/24/2022 14:07	5.099999905	59.40000153	24.29999924	84.5	5808.894	236
5/24/2022 14:08	5.099999905	59.29999924	24.29999924	84.59999847	5839.664	236
5/24/2022 14:09	5.099999905	59.29999924	24.29999924	84.59999847	5870.434	236
5/24/2022 14:10	5.099999905	59.29999924	24.29999924	84.59999847	5901.204	236
5/24/2022 14:11	5.059999943	59.29999924	24.20000076	84.59999847	5931.732	236
5/24/2022 14:12	5.059999943	59.29999924	24.20000076	84.69999695	5962.261	236
5/24/2022 14:13	5.059999943	59.29999924	24.20000076	84.69999695	5992.79	236
5/24/2022 14:14	5.019999981	59.29999924	24.20000076	84.69999695	6023.077	236
5/24/2022 14:15	5.019999981	59.29999924	24.20000076	84.69999695	6053.364	236
5/24/2022 14:16	5.019999981	59.29999924	24.10000038	84.80000305	6083.651	236
5/24/2022 14:17	5.019999981	59.20000076	24.10000038	84.80000305	6113.938	236
5/24/2022 14:18	5.269999981	59.29999924	24.20000076	85	6145.734	236
5/24/2022 14:19	5.230000019	59.29999924	24.10000038	85.09999847	6177.289	236
5/24/2022 14:20	5.190000057	59.29999924	24.10000038	85.09999847	6208.602	236
5/24/2022 14:21	5.190000057	59.20000076	24.10000038	85.09999847	6239.915	236
5/24/2022 14:22	5.150000095	59.20000076	24.10000038	85.09999847	6270.986	236
5/24/2022 14:23	5.150000095	59.20000076	24.10000038	85.19999695	6302.058	236
5/24/2022 14:24	5.150000095	59.20000076	24.10000038	85.19999695	6333.13	236
5/24/2022 14:25	5.099999905	59.20000076	24.10000038	85.19999695	6363.9	236
5/24/2022 14:26	5.099999905	59.09999847	24.10000038	85.19999695	6394.67	236
5/24/2022 14:27	5.099999905	59.09999847	24.10000038	85.30000305	6425.44	236
5/24/2022 14:28	5.099999905	59.09999847	24.10000038	85.30000305	6456.21	236
5/24/2022 14:29	5.099999905	59.09999847	24.10000038	85.30000305	6486.98	236
5/24/2022 14:30	5.059999943	59.09999847	24.10000038	85.40000153	6517.509	236
5/24/2022 14:31	5.059999943	59	24	85.40000153	6548.038	236
5/24/2022 14:32	5.059999943	59	24	85.40000153	6578.566	236
5/24/2022 14:33	5.019999981	59	24	85.40000153	6608.854	236
5/24/2022 14:34	5.019999981	59	24	85.40000153	6639.141	236
5/24/2022 14:35	5.019999981	59	24	85.5	6669.428	236
5/24/2022 14:36	5.019999981	59	24	85.5	6699.715	236
5/24/2022 14:37	5.150000095	59	24.10000038	85.59999847	6730.002	236
5/24/2022 14:38	5.230000019	59	24	85.80000305	6761.556	236
5/24/2022 14:39	5.190000057	59	24	85.80000305	6792.869	236
5/24/2022 14:40	5.190000057	58.90000153	24.10000038	85.80000305	6824.182	236

5/24/2022 14:41	5.150000095	58.90000153	24	85.90000153	6855.254	236
5/24/2022 14:42	5.150000095	58.90000153	24	85.90000153	6886.326	236
5/24/2022 14:43	5.099999905	58.90000153	24	85.90000153	6917.397	236
5/24/2022 14:44	5.099999905	58.90000153	24	85.90000153	6948.167	236
5/24/2022 14:45	5.099999905	58.90000153	24	85.90000153	6978.938	236
5/24/2022 14:46	5.099999905	58.90000153	24.10000038	85.90000153	7009.708	236
5/24/2022 14:47	5.099999905	58.90000153	24	86	7040.478	236
5/24/2022 14:48	5.099999905	58.90000153	24	86	7071.248	236
5/24/2022 14:49	5.059999943	58.90000153	24.10000038	86	7101.776	236
5/24/2022 14:50	5.059999943	58.79999924	24.10000038	86	7132.305	236
5/24/2022 14:51	5.059999943	58.79999924	24	86.09999847	7162.834	236
5/24/2022 14:52	5.019999981	58.70000076	24	86.09999847	7193.121	236
5/24/2022 14:53	5.019999981	58.79999924	24	86.09999847	7223.408	236
5/24/2022 14:54	5.019999981	58.79999924	24	86.19999695	7253.695	236
5/24/2022 14:55	5.019999981	58.70000076	24	86.19999695	7283.982	236
5/24/2022 14:56	5.019999981	58.70000076	24	86.19999695	7314.27	236
5/24/2022 14:57	5.269999981	58.70000076	24	86.40000153	7346.065	236
5/24/2022 14:58	5.230000019	58.70000076	23.89999962	86.5	7377.62	236
5/24/2022 14:59	5.190000057	58.70000076	23.89999962	86.5	7408.933	236
5/24/2022 15:00	5.190000057	58.70000076	23.89999962	86.5	7440.246	236
5/24/2022 15:01	5.190000057	58.70000076	23.89999962	86.5	7471.559	236
5/24/2022 15:02	5.150000095	58.70000076	23.89999962	86.59999847	7502.63	236
5/24/2022 15:03	5.150000095	58.70000076	24	86.59999847	7533.702	236
5/24/2022 15:04	5.150000095	58.70000076	23.89999962	86.59999847	7564.774	236
5/24/2022 15:05	5.099999905	58.70000076	24	86.59999847	7595.544	236
5/24/2022 15:06	5.099999905	58.59999847	24	86.69999695	7626.314	236
5/24/2022 15:07	5.099999905	58.59999847	24	86.69999695	7657.084	236
5/24/2022 15:08	5.099999905	58.59999847	24	86.69999695	7687.854	236
5/24/2022 15:09	5.099999905	58.59999847	24	86.69999695	7718.624	236
5/24/2022 15:10	5.059999943	58.59999847	24	86.69999695	7749.153	236
5/24/2022 15:11	5.059999943	58.5	24	86.80000305	7779.682	236
5/24/2022 15:12	5.059999943	58.5	24	86.80000305	7810.21	236
5/24/2022 15:13	5.059999943	58.5	23.89999962	86.80000305	7840.739	236
5/24/2022 15:14	5.019999981	58.5	23.89999962	86.80000305	7871.026	236

5/24/2022 15:15	5.019999981	58.40000153	23.79999924	86.80000305	7901.313	236
5/24/2022 15:16	5.019999981	58.5	23.79999924	86.80000305	7931.601	236
5/24/2022 15:17	5.019999981	58.40000153	23.79999924	86.90000153	7961.888	236
5/24/2022 15:18	5.349999905	58.5	23.79999924	87.09999847	7993.925	236
5/24/2022 15:19	5.059999943	58.40000153	23.79999924	83.09999847	8025.479	236
5/24/2022 15:20	2.980000019	58.5	23.79999924	45.90000153	8044.363	236
5/24/2022 15:21	1.820000052	58.5	23.79999924	26.10000038	8055.766	236
5/24/2022 15:22	1.059999943	58.5	23.79999924	67.09999847	8061.98	236
5/24/2022 15:23	0.879999995	58.40000153	23.79999924	37.29999924	8067.531	236
5/24/2022 15:24	0.730000019	58.40000153	23.79999924	66.5	8072.599	236
5/24/2022 15:25	0.439999998	58.40000153	23.89999962	54.5	8075.254	236
5/24/2022 15:26	0.360000014	58.5	23.79999924	30.5	8077.426	236
5/24/2022 15:27	0.219999999	58.40000153	23.89999962	83	8078.934	236
5/24/2022 15:28	0.219999999	58.29999924	23.89999962	45.90000153	8080.261	236
5/24/2022 15:29	0.219999999	58.40000153	23.89999962	66.40000153	8081.588	236
5/24/2022 15:30	0.140000001	58.29999924	23.89999962	54	8082.252	236
5/24/2022 15:31	0.109999999	58.29999924	23.89999962	30.39999962	8082.916	236
5/24/2022 15:32	0.07	58.29999924	23.89999962	64.19999695	8083.338	236
5/24/2022 15:33	0.07	58.29999924	23.79999924	35.70000076	8083.76	236
5/24/2022 15:34	0.07	58.29999924	23.79999924	77	8084.183	236
5/24/2022 15:35	0.07	58.20000076	23.79999924	42.5	8084.605	236
5/24/2022 15:36	0.039999999	58.20000076	23.79999924	84	8084.846	236
5/24/2022 15:37	0.039999999	58.29999924	23.79999924	50.40000153	8085.087	236
5/24/2022 15:38	0.039999999	58.20000076	23.79999924	34.29999924	8085.329	236
5/24/2022 15:39	0.039999999	58.20000076	23.79999924	60.20000076	8085.57	236
5/24/2022 15:40	0.039999999	58.20000076	23.79999924	33.79999924	8085.811	236
5/24/2022 15:41	0.039999999	58.20000076	23.79999924	71.5	8086.052	236
5/24/2022 15:42	0.039999999	58.20000076	23.89999962	39.90000153	8086.293	236
5/24/2022 15:43	0.039999999	58.20000076	23.79999924	86.30000305	8086.535	236
5/24/2022 15:44	0	58.20000076	23.79999924	47.40000153	8086.535	236
5/24/2022 15:45	0.039999999	58.20000076	23.89999962	53.59999847	8086.535	236

Date	CONC._A	CONC._B	TEMP_A	TEMP_B	RH_A	RH_B	VAC_A	VAC_B	DIFF_ALRN	DIFF_ALRN	RUNNUMBER_DOUBL
5/24/2022 9:36	0	0	24.34676	24.73749	43.8	42.5	68.3	68.8	0	0	236
5/24/2022 9:37	0	0	24.46886	24.81075	43	43.2	67.1	67.6	0	0	236
5/24/2022 9:38	0	0	24.46886	24.85959	45.5	45.8	67.2	67.7	0	0	236
5/24/2022 9:39	0	0	24.49329	24.884	46.4	46.6	67.3	67.8	0	0	236
5/24/2022 9:40	0	0	24.56654	24.90842	46.8	47.1	67.4	67.9	0	0	236
5/24/2022 9:41	0.036179	0	24.56654	24.93285	52	52.3	67.6	68.1	0	0	236
5/24/2022 9:42	0.036179	0	24.59097	24.93285	54.8	55.2	67.7	68.2	0	0	236
5/24/2022 9:43	0.036179	0	24.61539	24.93285	57.1	57.4	67.8	68.4	0	0	236
5/24/2022 9:44	0.036179	0	24.61539	24.93285	58.9	59.2	67.9	68.4	0	0	236
5/24/2022 9:45	0.036179	0	24.61539	24.93285	60	60.4	68	68.5	0	0	236
5/24/2022 9:46	0.036179	0	24.61539	24.93285	60.5	60.9	68.1	68.6	0	0	236
5/24/2022 9:47	0.036179	0	24.63981	24.93285	62	62.4	68.2	68.7	0	0	236
5/24/2022 9:48	0.036179	0	24.63981	24.93285	63.2	63.6	68.3	68.8	0	0	236
5/24/2022 9:49	0.036179	0	24.68864	24.95727	64.2	64.6	68.4	68.9	0	0	236
5/24/2022 9:50	0.036179	0	24.71307	24.98169	65	65.4	68.4	69	0	0	236
5/24/2022 9:51	0.036179	0	24.78632	25.0061	65.9	66.1	68.5	69	0	0	236
5/24/2022 9:52	0.036179	0	24.81075	25.0061	66.9	67	68.6	69.1	0	0	236
5/24/2022 9:53	0.036179	0	24.81075	25.0061	67.8	67.7	68.7	69.2	0	0	236
5/24/2022 9:54	0.036179	0	24.884	25.03053	68.7	68.6	68.8	69.3	0	0	236
5/24/2022 9:55	0.036179	0	24.884	25.07937	69.7	69.4	68.9	69.4	0	0	236
5/24/2022 9:56	0.036179	0	24.85959	25.03053	70.2	69.7	68.8	69.3	0	134	236
5/24/2022 9:57	0.036179	0	24.884	25.0061	67	66.5	68.8	69.3	0	0	236
5/24/2022 9:58	0.036179	0	24.884	25.05495	65.1	64.6	68.9	69.4	0	0	236
5/24/2022 9:59	0.036179	0	24.884	25.10378	67.3	67	69	69.6	0	0	236
5/24/2022 10:00	0.036179	0	24.884	25.07937	69.2	69	69.1	69.6	0	0	236
5/24/2022 10:01	0.036179	0	24.93285	25.07937	70.4	70.2	69.2	69.7	0	100	236
5/24/2022 10:02	0.036179	0	24.93285	25.07937	68.2	67.8	69.1	69.6	0	698	236
5/24/2022 10:03	0.036179	0	24.90842	25.10378	66.1	65.8	69.1	69.6	0	0	236
5/24/2022 10:04	0.036179	0	24.95727	25.15263	66.6	66.5	69.3	69.8	0	0	236
5/24/2022 10:05	0.036179	0	24.95727	25.15263	68.9	68.8	69.4	69.9	0	0	236
5/24/2022 10:06	0.036179	0	25.0061	25.15263	70.4	70.3	69.4	69.9	0	25	236
5/24/2022 10:07	0.036179	0	24.98169	25.1282	69	68.5	69.4	69.9	0	624	236
5/24/2022 10:08	0.036179	0	24.98169	25.15263	66.8	66.5	69.4	69.9	0	0	236

5/24/2022 10:09	0.036179	0	25.07937	25.20147	66.3	66.1	69.6	70	0	0	236
5/24/2022 10:10	0.036179	0	25.1282	25.25031	69.5	69.3	69.6	70.2	0	0	236
5/24/2022 10:11	0.036179	0	25.07937	25.25031	70.9	70.5	69.7	70.1	0	292	236
5/24/2022 10:12	0.036179	0	25.10378	25.27473	68.4	67.9	69.6	70.2	0	901	236
5/24/2022 10:13	0.036179	0	25.1282	25.29915	66.8	66.3	69.7	70.2	0	0	236
5/24/2022 10:14	0.036179	0	25.20147	25.32356	67.3	67	69.8	70.3	0	0	236
5/24/2022 10:15	0.036179	0	25.20147	25.34798	70.8	70.5	69.9	70.4	0	4	236
5/24/2022 10:16	0.036179	0	25.17705	25.32356	69.9	69.2	69.9	70.4	0	603	236
5/24/2022 10:17	0.036179	0	25.20147	25.32356	67.9	67.3	69.9	70.4	0	0	236
5/24/2022 10:18	0.036179	0	25.20147	25.34798	66.7	66.1	69.9	70.4	0	0	236
5/24/2022 10:19	0.036179	0	25.20147	25.37241	68.1	67.7	70.1	70.6	0	0	236
5/24/2022 10:20	0.036179	0	25.20147	25.42125	70.6	70.2	70.1	70.7	0	41	236
5/24/2022 10:21	0.036179	0	25.20147	25.39683	69.5	68.9	70.1	70.7	0	650	236
5/24/2022 10:22	0.036179	0	25.20147	25.39683	67.8	67.2	70.1	70.7	0	0	236
5/24/2022 10:23	0.036179	0	25.20147	25.42125	66.9	66.2	70.2	70.7	0	0	236
5/24/2022 10:24	0.036179	0	25.25031	25.47009	69.3	68.9	70.3	70.9	0	0	236
5/24/2022 10:25	0.036179	0	25.20147	25.44566	71.4	70.8	70.3	70.9	0	292	236
5/24/2022 10:26	0.036179	0	25.20147	25.47009	69.4	68.6	70.3	70.9	0	901	236
5/24/2022 10:27	0.036179	0	25.22588	25.47009	68	67.3	70.4	70.9	0	0	236
5/24/2022 10:28	0.036179	0	25.20147	25.44566	67.2	66.5	70.4	70.9	0	0	236
5/24/2022 10:29	0.036179	0	25.27473	25.51893	69.3	68.8	70.6	71.1	0	0	236
5/24/2022 10:30	0.036179	0	25.25031	25.49451	71.4	70.6	70.6	71.1	0	304	236
5/24/2022 10:31	0.036179	0	25.20147	25.51893	69.5	68.5	70.6	71.1	0	903	236
5/24/2022 10:32	0.036179	0	25.25031	25.51893	68.2	67.3	70.6	71.1	0	1501	236
5/24/2022 10:33	0.036179	0	25.25031	25.54335	67.4	66.5	70.6	71.1	0	0	236
5/24/2022 10:34	0.036179	0	25.32356	25.56776	68.3	67.6	70.8	71.3	0	0	236
5/24/2022 10:35	0.036179	0	25.27473	25.51893	71.9	71.1	70.9	71.4	0	209	236
5/24/2022 10:36	0.036179	0	25.27473	25.51893	70.4	69.4	70.8	71.4	0	808	236
5/24/2022 10:37	0.036179	0	25.27473	25.54335	68.8	67.9	70.9	71.4	0	1408	236
5/24/2022 10:38	0.036179	0	25.27473	25.54335	68	67.1	70.9	71.4	0	2017	236
5/24/2022 10:39	0.036179	0	25.27473	25.56776	67.4	66.5	70.9	71.4	0	0	236
5/24/2022 10:40	0.036179	0	25.39683	25.64103	69.6	68.9	71.1	71.6	0	0	236
5/24/2022 10:41	0.036179	0	25.32356	25.56776	72.3	71.4	71.1	71.6	0	375	236
5/24/2022 10:42	0.036179	0	25.27473	25.54335	70.4	69.4	71.1	71.6	0	982	236

5/24/2022 10:43	0.036179	0	25.27473	25.54335	69.1	68.2	71.1	71.6	0	1579	236
5/24/2022 10:44	0.036179	0	25.34798	25.56776	68.3	67.4	71.1	71.7	0	2175	236
5/24/2022 10:45	0.036179	0	25.29915	25.59219	67.8	66.8	71.2	71.7	0	0	236
5/24/2022 10:46	0.036179	0	25.34798	25.66544	67.5	66.5	71.3	71.8	0	0	236
5/24/2022 10:47	0.036179	0	25.34798	25.68987	71.4	70.5	71.4	71.9	0	66	236
5/24/2022 10:48	0.036179	0	25.27473	25.61661	71.3	70.2	71.4	71.9	0	662	236
5/24/2022 10:49	0.036179	0	25.34798	25.64103	69.8	68.6	71.4	71.9	0	1256	236
5/24/2022 10:50	0.036179	0	25.29915	25.61661	68.8	67.8	71.4	71.9	0	1865	236
5/24/2022 10:51	0.036179	0	25.32356	25.64103	68.2	67.3	71.5	72	0	2466	236
5/24/2022 10:52	0.036179	0	25.29915	25.64103	67.8	66.8	71.5	72	0	0	236
5/24/2022 10:53	0.036179	0	25.32356	25.66544	67.6	66.5	71.6	72.1	0	0	236
5/24/2022 10:54	0.036179	0	25.39683	25.71429	71.7	70.8	71.7	72.2	0	41	236
5/24/2022 10:55	0.036179	0	25.32356	25.64103	71.8	70.6	71.7	72.2	0	633	236
5/24/2022 10:56	0.036179	0	25.34798	25.64103	70.1	69	66.8	67.3	0	0	236
5/24/2022 10:57	0.953004	0.915875	25.37241	25.71429	69.9	68.9	67.8	68.3	0	0	236
5/24/2022 10:58	1.8555	1.779456	25.39683	25.81197	70	69	68.7	69.2	0	0	236
5/24/2022 10:59	2.508011	2.469324	25.42125	25.81197	70.1	69.1	69.5	70	0	0	236
5/24/2022 11:00	3.092876	3.053619	25.42125	25.81197	70.1	69	70.3	70.9	0	0	236
5/24/2022 11:01	3.686291	3.686291	25.42125	25.78754	70	68.9	71.1	71.7	0	0	236
5/24/2022 11:02	4.288256	4.247859	25.37241	25.78754	69.9	68.7	72	72.5	0	0	236
5/24/2022 11:03	4.816875	4.775984	25.34798	25.76313	69.7	68.5	72.8	73.4	0	0	236
5/24/2022 11:04	5.186604	5.145371	25.34798	25.73871	69.6	68.3	73.5	74.1	0	0	236
5/24/2022 11:05	5.145371	5.104176	25.32356	25.71429	69.4	68.1	73.8	74.3	157	0	236
5/24/2022 11:06	5.310531	5.269185	25.29915	25.68987	69.3	67.9	74.2	74.7	0	0	236
5/24/2022 11:07	5.063019	5.0219	25.25031	25.64103	69.1	67.7	74.2	74.7	511	0	236
5/24/2022 11:08	5.227875	5.186604	25.27473	25.64103	68.9	67.6	74.5	75.1	128	0	236
5/24/2022 11:09	5.063019	5.0219	25.25031	25.56776	68.7	67.4	74.5	75.1	730	0	236
5/24/2022 11:10	5.227875	5.186604	25.25031	25.51893	68.5	67.3	74.8	75.4	125	0	236
5/24/2022 11:11	5.104176	5.063019	25.20147	25.54335	68.4	67.1	74.9	75.4	713	0	236
5/24/2022 11:12	4.980819	4.939776	25.20147	25.49451	68.3	66.9	75	75.5	0	0	236
5/24/2022 11:13	5.186604	5.186604	25.17705	25.47009	68.1	66.8	75.2	75.8	344	0	236
5/24/2022 11:14	5.104176	5.063019	25.20147	25.42125	68	66.6	75.3	75.8	934	0	236
5/24/2022 11:15	5.0219	4.980819	25.17705	25.42125	67.8	66.5	75.3	75.9	1536	0	236
5/24/2022 11:16	5.269185	5.227875	25.15263	25.37241	67.7	66.3	75.6	76.2	170	0	236

5/24/2022 11:17	5.145371	5.145371	25.1282	25.34798	67.6	66.2	75.7	76.2	761	0	236
5/24/2022 11:18	5.104176	5.063019	25.1282	25.29915	67.4	66.1	75.7	76.2	1363	0	236
5/24/2022 11:19	5.0219	5.0219	25.07937	25.32356	67.3	66	75.7	76.3	1963	0	236
5/24/2022 11:20	5.269185	5.269185	25.03053	25.25031	67.1	65.9	76	76.6	12	0	236
5/24/2022 11:21	5.186604	5.145371	25.0061	25.22588	67	65.8	76	76.6	610	0	236
5/24/2022 11:22	5.104176	5.104176	24.95727	25.20147	66.9	65.7	76.1	76.6	1208	0	236
5/24/2022 11:23	5.063019	5.0219	24.95727	25.17705	66.8	65.6	76.1	76.6	1817	0	236
5/24/2022 11:24	5.0219	4.980819	24.90842	25.1282	66.6	65.5	76.1	76.7	2416	0	236
5/24/2022 11:25	5.269185	5.269185	24.90842	25.10378	66.5	65.4	76.4	77	111	0	236
5/24/2022 11:26	5.186604	5.145371	24.884	25.05495	66.4	65.3	76.5	77	699	0	236
5/24/2022 11:27	5.145371	5.104176	24.884	25.03053	66.3	65.3	76.5	77	1298	0	236
5/24/2022 11:28	5.104176	5.063019	24.884	25.0061	66.2	65.1	76.5	77.1	1896	0	236
5/24/2022 11:29	5.063019	5.0219	24.884	25.0061	66.1	65.1	76.5	77.1	2504	0	236
5/24/2022 11:30	5.0219	4.980819	24.884	24.93285	66	65	76.6	77.2	3107	0	236
5/24/2022 11:31	5.269185	5.227875	24.85959	24.93285	65.9	65	76.9	77.4	249	0	236
5/24/2022 11:32	5.186604	5.145371	24.81075	24.90842	65.7	64.9	76.9	77.5	850	0	236
5/24/2022 11:33	5.145371	5.104176	24.78632	24.884	65.7	64.9	76.9	77.5	1450	0	236
5/24/2022 11:34	5.104176	5.063019	24.76191	24.85959	65.6	64.8	77	77.5	2054	0	236
5/24/2022 11:35	5.063019	5.0219	24.71307	24.85959	65.5	64.7	77	77.5	2646	0	236
5/24/2022 11:36	5.0219	5.0219	24.71307	24.83517	65.3	64.7	77	77.6	3248	0	236
5/24/2022 11:37	5.310531	5.310531	24.66422	24.81075	65.3	64.6	77.3	77.8	0	0	236
5/24/2022 11:38	5.186604	5.186604	24.66422	24.76191	65.2	64.6	77.3	77.9	562	0	236
5/24/2022 11:39	5.145371	5.104176	24.66422	24.78632	65.1	64.5	77.3	77.9	1141	0	236
5/24/2022 11:40	5.104176	5.104176	24.63981	24.78632	65	64.5	77.4	77.9	1746	0	236
5/24/2022 11:41	5.063019	5.0219	24.61539	24.73749	64.9	64.4	77.4	78	2346	0	236
5/24/2022 11:42	5.0219	5.0219	24.61539	24.71307	64.8	64.3	77.4	78	2946	0	236
5/24/2022 11:43	5.0219	4.980819	24.59097	24.71307	64.7	64.3	77.5	78	3548	0	236
5/24/2022 11:44	5.269185	5.269185	24.59097	24.68864	64.7	64.2	77.8	78.3	256	0	236
5/24/2022 11:45	5.186604	5.186604	24.59097	24.68864	64.6	64.2	77.8	78.4	854	0	236
5/24/2022 11:46	5.145371	5.145371	24.61539	24.63981	64.5	64.2	77.8	78.4	1452	0	236
5/24/2022 11:47	5.145371	5.104176	24.61539	24.61539	64.4	64.1	77.8	78.4	2051	0	236
5/24/2022 11:48	5.104176	5.104176	24.59097	24.61539	64.3	64.1	77.9	78.5	2660	0	236
5/24/2022 11:49	5.063019	5.063019	24.59097	24.63981	64.2	64	77.9	78.5	3248	0	236
5/24/2022 11:50	5.063019	5.0219	24.59097	24.61539	64.2	64	78	78.5	3847	0	236

5/24/2022 11:51	5.0219	5.0219	24.59097	24.59097	64.1	63.9	78	78.6	4456	0	236
5/24/2022 11:52	5.310531	5.310531	24.56654	24.56654	64.1	63.9	78.3	78.9	0	0	236
5/24/2022 11:53	5.227875	5.227875	24.56654	24.56654	64	63.9	78.3	78.9	554	0	236
5/24/2022 11:54	5.186604	5.145371	24.56654	24.56654	63.9	63.8	78.3	78.9	1152	0	236
5/24/2022 11:55	5.145371	5.145371	24.51771	24.56654	63.8	63.8	78.4	78.9	1740	0	236
5/24/2022 11:56	5.104176	5.104176	24.51771	24.54212	63.8	63.7	78.4	79	2339	0	236
5/24/2022 11:57	5.104176	5.063019	24.51771	24.51771	63.7	63.6	78.5	79	2937	0	236
5/24/2022 11:58	5.063019	5.063019	24.49329	24.49329	63.6	63.7	78.5	79	3546	0	236
5/24/2022 11:59	5.0219	5.0219	24.49329	24.51771	63.5	63.6	78.5	79	4144	0	236
5/24/2022 12:00	5.0219	5.0219	24.46886	24.46886	63.5	63.6	78.6	79.1	4743	0	236
5/24/2022 12:01	5.269185	5.269185	24.44444	24.49329	63.4	63.5	78.8	79.4	0	0	236
5/24/2022 12:02	5.227875	5.227875	24.44444	24.46886	63.3	63.5	78.8	79.4	503	0	236
5/24/2022 12:03	5.186604	5.186604	24.44444	24.46886	63.3	63.5	78.9	79.4	1102	0	236
5/24/2022 12:04	5.145371	5.145371	24.39561	24.46886	63.2	63.4	78.9	79.5	1718	0	236
5/24/2022 12:05	5.104176	5.104176	24.42003	24.46886	63.2	63.4	78.9	79.5	2298	0	236
5/24/2022 12:06	5.104176	5.104176	24.34676	24.44444	63.1	63.4	79	79.5	2900	0	236
5/24/2022 12:07	5.063019	5.063019	24.39561	24.42003	63	63.3	79	79.5	3511	0	236
5/24/2022 12:08	5.063019	5.063019	24.37119	24.42003	63	63.3	79	79.6	4100	0	236
5/24/2022 12:09	5.0219	5.0219	24.39561	24.44444	63	63.3	79.1	79.6	4715	0	236
5/24/2022 12:10	5.0219	5.0219	24.37119	24.42003	62.9	63.2	79.1	79.6	5314	0	236
5/24/2022 12:11	5.393339	5.351916	24.39561	24.39561	62.9	63.2	79.4	80	0	0	236
5/24/2022 12:12	5.269185	5.227875	24.34676	24.44444	62.8	63.1	79.4	80	539	0	236
5/24/2022 12:13	5.186604	5.186604	24.37119	24.42003	62.8	63.1	79.5	80	1144	0	236
5/24/2022 12:14	5.186604	5.186604	24.37119	24.37119	62.7	63.1	79.5	80	1748	0	236
5/24/2022 12:15	5.145371	5.145371	24.37119	24.42003	62.7	63.1	79.5	80.1	2352	0	236
5/24/2022 12:16	5.145371	5.104176	24.37119	24.42003	62.6	63	79.6	80.1	2947	0	236
5/24/2022 12:17	5.104176	5.104176	24.34676	24.42003	62.6	63	79.6	80.1	3540	0	236
5/24/2022 12:18	5.104176	5.063019	24.37119	24.42003	62.5	62.9	79.6	80.2	4143	0	236
5/24/2022 12:19	5.063019	5.063019	24.34676	24.42003	62.5	62.9	79.6	80.2	4749	0	236
5/24/2022 12:20	5.063019	5.0219	24.34676	24.42003	62.5	62.9	79.7	80.2	5347	0	236
5/24/2022 12:21	5.0219	5.0219	24.34676	24.42003	62.5	62.9	79.7	80.2	5946	0	236
5/24/2022 12:22	5.0219	5.0219	24.34676	24.39561	62.3	62.8	79.7	80.3	0	0	236
5/24/2022 12:23	5.310531	5.310531	24.37119	24.42003	62.3	62.8	80	80.6	0	0	236
5/24/2022 12:24	5.227875	5.227875	24.37119	24.39561	62.3	62.8	80	80.6	0	0	236

5/24/2022 12:25	5.186604	5.186604	24.37119	24.42003	62.2	62.8	80	80.6	0	0	236
5/24/2022 12:26	5.145371	5.145371	24.37119	24.42003	62.2	62.7	80.1	80.7	0	0	236
5/24/2022 12:27	5.145371	5.104176	24.37119	24.44444	62.1	62.7	80.1	80.7	0	0	236
5/24/2022 12:28	5.104176	5.104176	24.37119	24.44444	62.1	62.7	80.1	80.7	0	0	236
5/24/2022 12:29	5.104176	5.104176	24.37119	24.42003	62.1	62.6	80.2	80.8	0	0	236
5/24/2022 12:30	5.063019	5.063019	24.37119	24.39561	62	62.6	80.2	80.8	0	0	236
5/24/2022 12:31	5.063019	5.0219	24.39561	24.44444	62	62.5	80.3	80.8	0	0	236
5/24/2022 12:32	5.0219	5.0219	24.34676	24.42003	61.9	62.5	80.3	80.9	0	0	236
5/24/2022 12:33	5.0219	5.0219	24.39561	24.42003	61.9	62.5	80.3	80.9	0	0	236
5/24/2022 12:34	5.0219	5.0219	24.39561	24.42003	61.8	62.4	80.3	80.9	0	0	236
5/24/2022 12:35	5.227875	5.227875	24.39561	24.44444	61.8	62.4	80.6	81.2	0	0	236
5/24/2022 12:36	5.186604	5.186604	24.39561	24.44444	61.8	62.4	80.6	81.2	0	0	236
5/24/2022 12:37	5.145371	5.145371	24.39561	24.46886	61.7	62.4	80.6	81.2	0	0	236
5/24/2022 12:38	5.145371	5.145371	24.39561	24.42003	61.7	62.3	80.7	81.3	0	0	236
5/24/2022 12:39	5.104176	5.104176	24.39561	24.44444	61.7	62.3	80.7	81.3	0	0	236
5/24/2022 12:40	5.104176	5.104176	24.39561	24.42003	61.6	62.3	80.8	81.3	0	0	236
5/24/2022 12:41	5.104176	5.063019	24.39561	24.46886	61.6	62.2	80.8	81.3	0	0	236
5/24/2022 12:42	5.063019	5.063019	24.37119	24.42003	61.6	62.2	80.8	81.4	0	0	236
5/24/2022 12:43	5.063019	5.063019	24.42003	24.44444	61.5	62.2	80.9	81.4	0	0	236
5/24/2022 12:44	5.0219	5.0219	24.39561	24.42003	61.5	62.1	80.9	81.4	0	0	236
5/24/2022 12:45	5.0219	5.0219	24.39561	24.44444	61.4	62.1	80.9	81.5	0	0	236
5/24/2022 12:46	5.0219	5.0219	24.37119	24.44444	61.4	62.1	80.9	81.5	0	0	236
5/24/2022 12:47	5.269185	5.269185	24.37119	24.44444	61.3	62.1	81.2	81.8	0	0	236
5/24/2022 12:48	5.227875	5.186604	24.39561	24.44444	61.4	62.1	81.2	81.8	0	0	236
5/24/2022 12:49	5.186604	5.186604	24.39561	24.44444	61.3	62	81.3	81.8	0	0	236
5/24/2022 12:50	5.145371	5.145371	24.39561	24.44444	61.3	62	81.3	81.9	0	0	236
5/24/2022 12:51	5.145371	5.145371	24.39561	24.44444	61.3	62	81.3	81.9	0	0	236
5/24/2022 12:52	5.104176	5.104176	24.39561	24.42003	61.2	62	81.4	81.9	0	0	236
5/24/2022 12:53	5.104176	5.104176	24.39561	24.44444	61.2	62	81.4	82	0	0	236
5/24/2022 12:54	5.104176	5.063019	24.39561	24.44444	61.1	61.9	81.4	82	0	0	236
5/24/2022 12:55	5.063019	5.063019	24.39561	24.44444	61.1	61.9	81.4	82	0	0	236
5/24/2022 12:56	5.063019	5.063019	24.39561	24.42003	61.1	61.9	81.5	82	0	0	236
5/24/2022 12:57	5.0219	5.0219	24.37119	24.46886	61.1	61.8	81.5	82.1	0	0	236
5/24/2022 12:58	5.0219	5.0219	24.39561	24.44444	61	61.8	81.5	82.1	0	0	236

5/24/2022 12:59	5.0219	5.0219	24.39561	24.42003	61	61.8	81.6	82.1	0	0	236
5/24/2022 13:00	5.269185	5.269185	24.42003	24.46886	61	61.8	81.8	82.4	0	0	236
5/24/2022 13:01	5.227875	5.186604	24.42003	24.42003	60.9	61.7	81.8	82.4	0	0	236
5/24/2022 13:02	5.186604	5.186604	24.39561	24.42003	60.9	61.7	81.8	82.5	0	0	236
5/24/2022 13:03	5.145371	5.145371	24.39561	24.44444	60.9	61.7	81.9	82.5	0	0	236
5/24/2022 13:04	5.145371	5.145371	24.39561	24.46886	60.8	61.6	81.9	82.5	0	0	236
5/24/2022 13:05	5.104176	5.104176	24.39561	24.46886	60.8	61.6	81.9	82.5	0	0	236
5/24/2022 13:06	5.104176	5.104176	24.39561	24.44444	60.8	61.6	82	82.5	0	0	236
5/24/2022 13:07	5.104176	5.063019	24.39561	24.42003	60.8	61.6	82	82.6	0	0	236
5/24/2022 13:08	5.063019	5.063019	24.39561	24.44444	60.7	61.5	82	82.6	0	0	236
5/24/2022 13:09	5.063019	5.063019	24.42003	24.44444	60.7	61.6	82.1	82.6	0	0	236
5/24/2022 13:10	5.0219	5.063019	24.42003	24.46886	60.7	61.5	82.1	82.6	0	0	236
5/24/2022 13:11	5.0219	5.0219	24.39561	24.46886	60.7	61.5	82.1	82.7	0	0	236
5/24/2022 13:12	5.0219	5.0219	24.39561	24.46886	60.6	61.5	82.1	82.7	0	0	236
5/24/2022 13:13	5.104176	5.104176	24.42003	24.44444	60.6	61.4	82.3	82.9	0	0	236
5/24/2022 13:14	5.227875	5.227875	24.44444	24.46886	60.6	61.4	82.5	83	0	0	236
5/24/2022 13:15	5.186604	5.186604	24.46886	24.44444	60.5	61.4	82.5	83	0	0	236
5/24/2022 13:16	5.186604	5.186604	24.44444	24.46886	60.5	61.3	82.5	83.1	0	0	236
5/24/2022 13:17	5.145371	5.145371	24.42003	24.46886	60.5	61.3	82.5	83.1	0	0	236
5/24/2022 13:18	5.145371	5.145371	24.44444	24.46886	60.4	61.3	82.6	83.1	0	0	236
5/24/2022 13:19	5.104176	5.104176	24.42003	24.46886	60.4	61.2	82.6	83.1	0	0	236
5/24/2022 13:20	5.104176	5.104176	24.44444	24.46886	60.4	61.2	82.6	83.2	0	0	236
5/24/2022 13:21	5.104176	5.104176	24.42003	24.46886	60.4	61.2	82.6	83.2	0	0	236
5/24/2022 13:22	5.104176	5.063019	24.39561	24.46886	60.3	61.2	82.7	83.3	0	0	236
5/24/2022 13:23	5.063019	5.063019	24.42003	24.46886	60.3	61.1	82.7	83.3	0	0	236
5/24/2022 13:24	5.063019	5.063019	24.39561	24.44444	60.3	61.2	82.7	83.3	0	0	236
5/24/2022 13:25	5.0219	5.0219	24.39561	24.46886	60.2	61.1	82.7	83.3	0	0	236
5/24/2022 13:26	5.0219	5.0219	24.39561	24.44444	60.2	61.1	82.7	83.4	0	0	236
5/24/2022 13:27	5.0219	5.0219	24.39561	24.44444	60.2	61.1	82.8	83.4	0	0	236
5/24/2022 13:28	4.980819	5.0219	24.39561	24.46886	60.1	61.1	82.8	83.4	0	0	236
5/24/2022 13:29	5.227875	5.227875	24.39561	24.44444	60.2	61	83.1	83.7	0	0	236
5/24/2022 13:30	5.186604	5.186604	24.39561	24.44444	60.1	61	83.1	83.7	0	0	236
5/24/2022 13:31	5.145371	5.145371	24.39561	24.44444	60.1	61	83.1	83.7	0	0	236
5/24/2022 13:32	5.145371	5.145371	24.37119	24.44444	60.1	61	83.1	83.7	0	0	236

5/24/2022 10:43	0.039999999	69.09999847	25.29999924	71.09999847	0	236
5/24/2022 10:44	0.039999999	68.30000305	25.29999924	71.09999847	0	236
5/24/2022 10:45	0.039999999	67.80000305	25.29999924	71.19999695	0	236
5/24/2022 10:46	0.039999999	67.5	25.29999924	71.30000305	0	236
5/24/2022 10:47	0.039999999	71.40000153	25.29999924	71.40000153	0	236
5/24/2022 10:48	0.039999999	71.30000305	25.29999924	71.40000153	0	236
5/24/2022 10:49	0.039999999	69.80000305	25.29999924	71.40000153	0	236
5/24/2022 10:50	0.039999999	68.80000305	25.29999924	71.40000153	0	236
5/24/2022 10:51	0.039999999	68.19999695	25.29999924	71.5	0	236
5/24/2022 10:52	0.039999999	67.80000305	25.29999924	71.5	0	236
5/24/2022 10:53	0.039999999	67.59999847	25.29999924	71.59999847	0	236
5/24/2022 10:54	0.039999999	71.69999695	25.39999962	71.69999695	0	236
5/24/2022 10:55	0.039999999	71.80000305	25.29999924	71.69999695	0	236
5/24/2022 10:56	0.039999999	70.09999847	25.29999924	66.80000305	0	236
5/24/2022 10:57	0.949999988	69.90000153	25.29999924	67.80000305	5.309333	236
5/24/2022 10:58	1.820000052	70	25.39999962	68.69999695	15.566	236
5/24/2022 10:59	2.50999999	70.09999847	25.39999962	69.5	30.227	236
5/24/2022 11:00	3.089999914	70.09999847	25.39999962	70.30000305	48.62867	236
5/24/2022 11:01	3.730000019	70	25.39999962	71.19999695	70.65034	236
5/24/2022 11:02	4.289999962	69.90000153	25.39999962	72	96.05067	236
5/24/2022 11:03	4.820000172	69.69999695	25.29999924	72.80000305	124.6487	236
5/24/2022 11:04	5.190000057	69.59999847	25.29999924	73.5	155.7203	236
5/24/2022 11:05	5.150000095	69.40000153	25.29999924	73.80000305	187.0333	236
5/24/2022 11:06	5.309999943	69.30000305	25.29999924	74.19999695	218.5877	236
5/24/2022 11:07	5.059999943	69.09999847	25.29999924	74.19999695	249.3577	236
5/24/2022 11:08	5.230000019	68.90000153	25.29999924	74.5	280.912	236
5/24/2022 11:09	5.059999943	68.69999695	25.20000076	74.5	311.4407	236
5/24/2022 11:10	5.230000019	68.5	25.20000076	74.90000153	343.2363	236
5/24/2022 11:11	5.099999905	68.40000153	25.20000076	74.90000153	374.0063	236
5/24/2022 11:12	4.980000019	68.30000305	25.20000076	75	404.2936	236
5/24/2022 11:13	5.190000057	68.09999847	25.20000076	75.19999695	435.848	236
5/24/2022 11:14	5.099999905	68	25.20000076	75.30000305	466.618	236
5/24/2022 11:15	5.019999981	67.80000305	25.20000076	75.30000305	496.9053	236
5/24/2022 11:16	5.269999981	67.69999695	25.20000076	75.59999847	528.7009	236

5/24/2022 13:33	5.145371	5.104176	24.34676	24.46886	60	60.9	83.2	83.8	0	0	236
5/24/2022 13:34	5.104176	5.104176	24.34676	24.46886	60	61	83.2	83.8	0	0	236
5/24/2022 13:35	5.104176	5.104176	24.34676	24.44444	60	60.9	83.2	83.8	0	0	236
5/24/2022 13:36	5.104176	5.104176	24.37119	24.42003	60	60.9	83.3	83.9	0	0	236
5/24/2022 13:37	5.063019	5.063019	24.34676	24.44444	60	60.9	83.3	83.9	0	0	236
5/24/2022 13:38	5.063019	5.063019	24.34676	24.44444	60	60.9	83.3	83.9	0	0	236
5/24/2022 13:39	5.0219	5.0219	24.34676	24.44444	59.9	60.8	83.4	83.9	0	0	236
5/24/2022 13:40	5.0219	5.0219	24.34676	24.39561	59.9	60.8	83.4	84	0	0	236
5/24/2022 13:41	5.0219	5.0219	24.32234	24.42003	59.9	60.8	83.4	84	0	0	236
5/24/2022 13:42	5.0219	5.0219	24.32234	24.42003	59.8	60.8	83.4	84	0	0	236
5/24/2022 13:43	5.269185	5.269185	24.32234	24.42003	59.8	60.8	83.7	84.3	0	0	236
5/24/2022 13:44	5.227875	5.227875	24.32234	24.42003	59.8	60.7	83.7	84.3	0	0	236
5/24/2022 13:45	5.186604	5.186604	24.32234	24.42003	59.7	60.7	83.8	84.3	0	0	236
5/24/2022 13:46	5.186604	5.186604	24.32234	24.39561	59.7	60.7	83.8	84.3	0	0	236
5/24/2022 13:47	5.145371	5.145371	24.32234	24.42003	59.7	60.7	83.8	84.4	0	0	236
5/24/2022 13:48	5.145371	5.145371	24.32234	24.37119	59.7	60.7	83.8	84.4	0	0	236
5/24/2022 13:49	5.104176	5.145371	24.32234	24.39561	59.7	60.6	83.8	84.4	0	0	236
5/24/2022 13:50	5.104176	5.104176	24.29793	24.39561	59.7	60.6	83.9	84.4	0	0	236
5/24/2022 13:51	5.104176	5.104176	24.32234	24.37119	59.7	60.6	83.9	84.5	0	0	236
5/24/2022 13:52	5.104176	5.104176	24.32234	24.37119	59.6	60.6	83.9	84.5	0	0	236
5/24/2022 13:53	5.104176	5.063019	24.32234	24.42003	59.7	60.6	83.9	84.5	0	0	236
5/24/2022 13:54	5.063019	5.063019	24.32234	24.37119	59.6	60.5	84	84.6	0	0	236
5/24/2022 13:55	5.063019	5.063019	24.32234	24.39561	59.6	60.5	84	84.6	0	0	236
5/24/2022 13:56	5.0219	5.063019	24.32234	24.37119	59.6	60.5	84	84.6	0	0	236
5/24/2022 13:57	5.0219	5.0219	24.32234	24.39561	59.6	60.5	84	84.7	0	0	236
5/24/2022 13:58	5.0219	5.0219	24.32234	24.39561	59.5	60.5	84.1	84.7	0	0	236
5/24/2022 13:59	5.0219	5.0219	24.32234	24.39561	59.5	60.5	84.1	84.7	0	0	236
5/24/2022 14:00	5.145371	5.145371	24.29793	24.37119	59.5	60.4	84.3	84.9	0	0	236
5/24/2022 14:01	5.227875	5.227875	24.32234	24.39561	59.5	60.4	84.4	85	0	0	236
5/24/2022 14:02	5.186604	5.186604	24.32234	24.39561	59.5	60.4	84.4	85	0	0	236
5/24/2022 14:03	5.186604	5.186604	24.32234	24.37119	59.5	60.4	84.5	85	0	0	236
5/24/2022 14:04	5.145371	5.145371	24.32234	24.39561	59.4	60.3	84.5	85.1	0	0	236
5/24/2022 14:05	5.145371	5.145371	24.32234	24.37119	59.4	60.3	84.5	85.1	0	0	236
5/24/2022 14:06	5.104176	5.104176	24.32234	24.37119	59.4	60.3	84.5	85.1	0	0	236

5/24/2022 14:07	5.104176	5.104176	24.32234	24.34676	59.4	60.3	84.5	85.1	0	0	236
5/24/2022 14:08	5.104176	5.104176	24.32234	24.34676	59.3	60.3	84.6	85.2	0	0	236
5/24/2022 14:09	5.104176	5.104176	24.29793	24.32234	59.3	60.3	84.6	85.2	0	0	236
5/24/2022 14:10	5.104176	5.063019	24.27351	24.32234	59.3	60.2	84.6	85.2	0	0	236
5/24/2022 14:11	5.063019	5.063019	24.24908	24.29793	59.3	60.2	84.6	85.2	0	0	236
5/24/2022 14:12	5.063019	5.063019	24.22466	24.29793	59.3	60.2	84.7	85.2	0	0	236
5/24/2022 14:13	5.063019	5.063019	24.20025	24.29793	59.3	60.2	84.7	85.3	0	0	236
5/24/2022 14:14	5.0219	5.0219	24.15141	24.29793	59.3	60.2	84.7	85.3	0	0	236
5/24/2022 14:15	5.0219	5.0219	24.15141	24.24908	59.2	60.1	84.7	85.3	0	0	236
5/24/2022 14:16	5.0219	5.0219	24.15141	24.24908	59.3	60.2	84.7	85.3	0	0	236
5/24/2022 14:17	5.0219	5.0219	24.10256	24.24908	59.2	60.1	84.8	85.4	0	0	236
5/24/2022 14:18	5.269185	5.269185	24.15141	24.22466	59.2	60.2	85	85.6	0	0	236
5/24/2022 14:19	5.227875	5.227875	24.12698	24.22466	59.2	60.1	85.1	85.6	0	0	236
5/24/2022 14:20	5.186604	5.186604	24.10256	24.22466	59.3	60.1	85.1	85.7	0	0	236
5/24/2022 14:21	5.186604	5.186604	24.07815	24.22466	59.2	60.1	85.1	85.7	0	0	236
5/24/2022 14:22	5.145371	5.145371	24.07815	24.22466	59.2	60.1	85.2	85.8	0	0	236
5/24/2022 14:23	5.145371	5.145371	24.07815	24.20025	59.1	60.1	85.2	85.8	0	0	236
5/24/2022 14:24	5.145371	5.145371	24.12698	24.22466	59.2	60.1	85.2	85.8	0	0	236
5/24/2022 14:25	5.104176	5.104176	24.10256	24.22466	59.2	60	85.2	85.8	0	0	236
5/24/2022 14:26	5.104176	5.104176	24.07815	24.22466	59.1	60.1	85.2	85.9	0	0	236
5/24/2022 14:27	5.104176	5.104176	24.10256	24.20025	59.1	60	85.3	85.9	0	0	236
5/24/2022 14:28	5.104176	5.104176	24.10256	24.20025	59.1	60	85.3	85.9	0	0	236
5/24/2022 14:29	5.104176	5.063019	24.07815	24.17583	59.1	59.9	85.3	85.9	0	0	236
5/24/2022 14:30	5.063019	5.063019	24.05373	24.20025	59	60	85.4	86	0	0	236
5/24/2022 14:31	5.063019	5.063019	24.05373	24.17583	59	60	85.4	86	0	0	236
5/24/2022 14:32	5.063019	5.0219	24.0293	24.17583	59	59.9	85.4	86	0	0	236
5/24/2022 14:33	5.0219	5.0219	24.0293	24.15141	59	59.9	85.4	86	0	0	236
5/24/2022 14:34	5.0219	5.0219	24.05373	24.15141	59	59.9	85.4	86	0	0	236
5/24/2022 14:35	5.0219	5.0219	24.0293	24.15141	59	59.9	85.5	86.1	0	0	236
5/24/2022 14:36	5.0219	5.0219	24.0293	24.15141	59	59.9	85.5	86.1	0	0	236
5/24/2022 14:37	5.145371	5.145371	24.0293	24.12698	59	59.9	85.7	86.3	0	0	236
5/24/2022 14:38	5.227875	5.227875	24.0293	24.15141	59	59.9	85.8	86.4	0	0	236
5/24/2022 14:39	5.186604	5.186604	24.0293	24.15141	59	59.9	85.8	86.4	0	0	236
5/24/2022 14:40	5.186604	5.186604	24.0293	24.12698	59	59.8	85.8	86.4	0	0	236

5/24/2022 14:41	5.145371	5.145371	24.0293	24.12698	58.9	59.9	85.9	86.4	0	0	236
5/24/2022 14:42	5.145371	5.145371	24.0293	24.12698	58.9	59.8	85.9	86.5	0	0	236
5/24/2022 14:43	5.145371	5.145371	24.00488	24.10256	58.9	59.8	85.9	86.5	0	0	236
5/24/2022 14:44	5.104176	5.104176	24.05373	24.12698	58.9	59.8	85.9	86.5	0	0	236
5/24/2022 14:45	5.104176	5.104176	24.00488	24.12698	58.9	59.8	85.9	86.6	0	0	236
5/24/2022 14:46	5.104176	5.104176	24.05373	24.12698	58.9	59.8	86	86.6	0	0	236
5/24/2022 14:47	5.104176	5.104176	24.0293	24.12698	58.9	59.8	86	86.6	0	0	236
5/24/2022 14:48	5.104176	5.063019	24.0293	24.12698	58.9	59.7	86	86.6	0	0	236
5/24/2022 14:49	5.063019	5.063019	24.05373	24.10256	58.9	59.7	86	86.6	0	0	236
5/24/2022 14:50	5.063019	5.063019	24.0293	24.12698	58.8	59.7	86	86.6	0	0	236
5/24/2022 14:51	5.063019	5.063019	24.00488	24.10256	58.8	59.7	86.1	86.7	0	0	236
5/24/2022 14:52	5.0219	5.0219	24.0293	24.15141	58.7	59.6	86.1	86.7	0	0	236
5/24/2022 14:53	5.0219	5.0219	24.0293	24.05373	58.8	59.6	86.1	86.7	0	0	236
5/24/2022 14:54	5.0219	5.0219	24.00488	24.05373	58.8	59.6	86.2	86.7	0	0	236
5/24/2022 14:55	5.0219	5.0219	24.00488	24.05373	58.7	59.6	86.2	86.8	0	0	236
5/24/2022 14:56	5.0219	5.0219	23.95604	24.05373	58.7	59.5	86.2	86.8	0	0	236
5/24/2022 14:57	5.269185	5.269185	23.98046	24.07815	58.8	59.6	86.5	87	0	0	236
5/24/2022 14:58	5.227875	5.227875	23.95604	24.05373	58.7	59.6	86.5	87.1	0	0	236
5/24/2022 14:59	5.186604	5.186604	23.95604	24.05373	58.7	59.6	86.5	87.1	0	0	236
5/24/2022 15:00	5.186604	5.186604	23.93163	24.0293	58.7	59.5	86.5	87.1	0	0	236
5/24/2022 15:01	5.186604	5.145371	23.93163	24.00488	58.7	59.6	86.5	87.1	0	0	236
5/24/2022 15:02	5.145371	5.145371	23.95604	24.0293	58.7	59.5	86.5	87.2	0	0	236
5/24/2022 15:03	5.145371	5.145371	23.98046	24.0293	58.7	59.6	86.6	87.2	0	0	236
5/24/2022 15:04	5.145371	5.145371	23.95604	24.0293	58.7	59.5	86.6	87.2	0	0	236
5/24/2022 15:05	5.104176	5.104176	23.93163	24.00488	58.7	59.5	86.6	87.2	0	0	236
5/24/2022 15:06	5.104176	5.104176	23.95604	24.0293	58.6	59.5	86.7	87.2	0	0	236
5/24/2022 15:07	5.104176	5.104176	24.00488	24.0293	58.6	59.5	86.7	87.3	0	0	236
5/24/2022 15:08	5.104176	5.104176	23.95604	24.05373	58.6	59.5	86.7	87.3	0	0	236
5/24/2022 15:09	5.104176	5.063019	23.98046	24.0293	58.6	59.4	86.7	87.3	0	0	236
5/24/2022 15:10	5.063019	5.063019	23.95604	24.05373	58.6	59.4	86.7	87.3	0	0	236
5/24/2022 15:11	5.063019	5.063019	24.00488	24.05373	58.5	59.4	86.7	87.3	0	0	236
5/24/2022 15:12	5.063019	5.063019	23.93163	24.0293	58.5	59.4	86.8	87.4	0	0	236
5/24/2022 15:13	5.0219	5.0219	23.88279	24.00488	58.4	59.3	86.8	87.4	0	0	236
5/24/2022 15:14	5.0219	5.0219	23.85836	23.98046	58.5	59.4	86.8	87.4	0	0	236

5/24/2022 15:15	5.0219	5.0219	23.80952	23.98046	58.4	59.3	86.8	87.4	0	0	236
5/24/2022 15:16	5.0219	5.0219	23.80952	23.98046	58.5	59.3	86.8	87.4	0	0	236
5/24/2022 15:17	5.0219	5.0219	23.80952	23.98046	58.4	59.3	86.8	87.4	0	0	236
5/24/2022 15:18	5.393339	5.393339	23.80952	23.95604	58.4	59.3	87.1	87.7	0	0	236
5/24/2022 15:19	5.063019	5.063019	23.78511	23.95604	58.4	59.3	83.3	83.9	0	0	236
5/24/2022 15:20	2.975219	2.975219	23.80952	23.93163	58.5	59.3	46.3	46.6	0	0	236
5/24/2022 15:21	1.817459	1.817459	23.76068	23.93163	58.5	59.3	26.3	26.5	0	0	236
5/24/2022 15:22	1.064619	1.064619	23.80952	23.93163	58.5	59.2	67.1	67.6	0	0	236
5/24/2022 15:23	0.878784	0.878784	23.78511	23.95604	58.4	59.3	37.4	37.7	0	0	236
5/24/2022 15:24	0.7308	0.7308	23.80952	23.9072	58.5	59.2	65.8	66.2	0	0	236
5/24/2022 15:25	0.436656	0.473291	23.85836	23.98046	58.4	59.3	54.2	54.6	0	0	236
5/24/2022 15:26	0.3635	0.400059	23.83395	23.95604	58.4	59.2	30.4	30.7	0	0	236
5/24/2022 15:27	0.217644	0.254051	23.85836	23.98046	58.4	59.3	82.7	83.3	0	0	236
5/24/2022 15:28	0.217644	0.254051	23.88279	23.98046	58.4	59.2	45.8	46.2	0	0	236
5/24/2022 15:29	0.217644	0.217644	23.85836	23.98046	58.4	59.2	64.2	64.7	0	0	236
5/24/2022 15:30	0.144944	0.144944	23.88279	23.98046	58.3	59.2	54.5	54.9	0	0	236
5/24/2022 15:31	0.108651	0.144944	23.85836	23.98046	58.3	59.1	30.3	30.6	0	0	236
5/24/2022 15:32	0.072396	0.108651	23.85836	23.98046	58.3	59.1	64.3	64.8	0	0	236
5/24/2022 15:33	0.072396	0.108651	23.83395	23.98046	58.2	59.1	35.9	36.2	0	0	236
5/24/2022 15:34	0.072396	0.072396	23.83395	23.95604	58.2	59.1	76.8	77.3	0	0	236
5/24/2022 15:35	0.072396	0.072396	23.78511	23.95604	58.3	59.1	42.6	43	0	0	236
5/24/2022 15:36	0.072396	0.072396	23.78511	23.93163	58.2	59.1	82.9	83.5	0	0	236
5/24/2022 15:37	0.036179	0.072396	23.76068	23.93163	58.3	59	50.8	51.2	0	0	236
5/24/2022 15:38	0.036179	0.072396	23.76068	23.93163	58.2	59	32.8	33	0	0	236
5/24/2022 15:39	0.036179	0.072396	23.78511	23.93163	58.3	59	59.9	60.4	0	0	236
5/24/2022 15:40	0.036179	0.072396	23.78511	23.9072	58.2	59.1	33.6	33.9	0	0	236
5/24/2022 15:41	0.036179	0.072396	23.78511	23.9072	58.2	59	71.8	72.3	0	0	236
5/24/2022 15:42	0.036179	0.072396	23.85836	23.9072	58.2	59	40	40.3	0	0	236
5/24/2022 15:43	0.036179	0.072396	23.78511	23.93163	58.2	59	86.4	87.1	0	0	236
5/24/2022 15:44	0	0.072396	23.83395	23.93163	58.2	59	47.6	48	0	0	236
5/24/2022 15:45	0.036179	0.072396	23.85836	23.93163	58.2	59	52.1	52.6	0	0	236

Date	CONC._A	CONC._B	TEMP_A	TEMP_B	RH_A	RH_B	VAC_A	VAC_B	DIFF_ALRN	DIFF_ALRN	RUNNUMBER_DOUBL
5/24/2022 9:36	0	0	24.34676	24.73749	43.8	42.5	68.3	68.8	0	0	236
5/24/2022 9:37	0	0	24.46886	24.81075	43	43.2	67.1	67.6	0	0	236
5/24/2022 9:38	0	0	24.46886	24.85959	45.5	45.8	67.2	67.7	0	0	236
5/24/2022 9:39	0	0	24.49329	24.884	46.4	46.6	67.3	67.8	0	0	236
5/24/2022 9:40	0	0	24.56654	24.90842	46.8	47.1	67.4	67.9	0	0	236
5/24/2022 9:41	0.036179	0	24.56654	24.93285	52	52.3	67.6	68.1	0	0	236
5/24/2022 9:42	0.036179	0	24.59097	24.93285	54.8	55.2	67.7	68.2	0	0	236
5/24/2022 9:43	0.036179	0	24.61539	24.93285	57.1	57.4	67.8	68.4	0	0	236
5/24/2022 9:44	0.036179	0	24.61539	24.93285	58.9	59.2	67.9	68.4	0	0	236
5/24/2022 9:45	0.036179	0	24.61539	24.93285	60	60.4	68	68.5	0	0	236
5/24/2022 9:46	0.036179	0	24.61539	24.93285	60.5	60.9	68.1	68.6	0	0	236
5/24/2022 9:47	0.036179	0	24.63981	24.93285	62	62.4	68.2	68.7	0	0	236
5/24/2022 9:48	0.036179	0	24.63981	24.93285	63.2	63.6	68.3	68.8	0	0	236
5/24/2022 9:49	0.036179	0	24.68864	24.95727	64.2	64.6	68.4	68.9	0	0	236
5/24/2022 9:50	0.036179	0	24.71307	24.98169	65	65.4	68.4	69	0	0	236
5/24/2022 9:51	0.036179	0	24.78632	25.0061	65.9	66.1	68.5	69	0	0	236
5/24/2022 9:52	0.036179	0	24.81075	25.0061	66.9	67	68.6	69.1	0	0	236
5/24/2022 9:53	0.036179	0	24.81075	25.0061	67.8	67.7	68.7	69.2	0	0	236
5/24/2022 9:54	0.036179	0	24.884	25.03053	68.7	68.6	68.8	69.3	0	0	236
5/24/2022 9:55	0.036179	0	24.884	25.07937	69.7	69.4	68.9	69.4	0	0	236
5/24/2022 9:56	0.036179	0	24.85959	25.03053	70.2	69.7	68.8	69.3	0	134	236
5/24/2022 9:57	0.036179	0	24.884	25.0061	67	66.5	68.8	69.3	0	0	236
5/24/2022 9:58	0.036179	0	24.884	25.05495	65.1	64.6	68.9	69.4	0	0	236
5/24/2022 9:59	0.036179	0	24.884	25.10378	67.3	67	69	69.6	0	0	236
5/24/2022 10:00	0.036179	0	24.884	25.07937	69.2	69	69.1	69.6	0	0	236
5/24/2022 10:01	0.036179	0	24.93285	25.07937	70.4	70.2	69.2	69.7	0	100	236
5/24/2022 10:02	0.036179	0	24.93285	25.07937	68.2	67.8	69.1	69.6	0	698	236
5/24/2022 10:03	0.036179	0	24.90842	25.10378	66.1	65.8	69.1	69.6	0	0	236
5/24/2022 10:04	0.036179	0	24.95727	25.15263	66.6	66.5	69.3	69.8	0	0	236
5/24/2022 10:05	0.036179	0	24.95727	25.15263	68.9	68.8	69.4	69.9	0	0	236
5/24/2022 10:06	0.036179	0	25.0061	25.15263	70.4	70.3	69.4	69.9	0	25	236
5/24/2022 10:07	0.036179	0	24.98169	25.1282	69	68.5	69.4	69.9	0	624	236
5/24/2022 10:08	0.036179	0	24.98169	25.15263	66.8	66.5	69.4	69.9	0	0	236

5/24/2022 10:09	0.036179	0	25.07937	25.20147	66.3	66.1	69.6	70	0	0	236
5/24/2022 10:10	0.036179	0	25.1282	25.25031	69.5	69.3	69.6	70.2	0	0	236
5/24/2022 10:11	0.036179	0	25.07937	25.25031	70.9	70.5	69.7	70.1	0	292	236
5/24/2022 10:12	0.036179	0	25.10378	25.27473	68.4	67.9	69.6	70.2	0	901	236
5/24/2022 10:13	0.036179	0	25.1282	25.29915	66.8	66.3	69.7	70.2	0	0	236
5/24/2022 10:14	0.036179	0	25.20147	25.32356	67.3	67	69.8	70.3	0	0	236
5/24/2022 10:15	0.036179	0	25.20147	25.34798	70.8	70.5	69.9	70.4	0	4	236
5/24/2022 10:16	0.036179	0	25.17705	25.32356	69.9	69.2	69.9	70.4	0	603	236
5/24/2022 10:17	0.036179	0	25.20147	25.32356	67.9	67.3	69.9	70.4	0	0	236
5/24/2022 10:18	0.036179	0	25.20147	25.34798	66.7	66.1	69.9	70.4	0	0	236
5/24/2022 10:19	0.036179	0	25.20147	25.37241	68.1	67.7	70.1	70.6	0	0	236
5/24/2022 10:20	0.036179	0	25.20147	25.42125	70.6	70.2	70.1	70.7	0	41	236
5/24/2022 10:21	0.036179	0	25.20147	25.39683	69.5	68.9	70.1	70.7	0	650	236
5/24/2022 10:22	0.036179	0	25.20147	25.39683	67.8	67.2	70.1	70.7	0	0	236
5/24/2022 10:23	0.036179	0	25.20147	25.42125	66.9	66.2	70.2	70.7	0	0	236
5/24/2022 10:24	0.036179	0	25.25031	25.47009	69.3	68.9	70.3	70.9	0	0	236
5/24/2022 10:25	0.036179	0	25.20147	25.44566	71.4	70.8	70.3	70.9	0	292	236
5/24/2022 10:26	0.036179	0	25.20147	25.47009	69.4	68.6	70.3	70.9	0	901	236
5/24/2022 10:27	0.036179	0	25.22588	25.47009	68	67.3	70.4	70.9	0	0	236
5/24/2022 10:28	0.036179	0	25.20147	25.44566	67.2	66.5	70.4	70.9	0	0	236
5/24/2022 10:29	0.036179	0	25.27473	25.51893	69.3	68.8	70.6	71.1	0	0	236
5/24/2022 10:30	0.036179	0	25.25031	25.49451	71.4	70.6	70.6	71.1	0	304	236
5/24/2022 10:31	0.036179	0	25.20147	25.51893	69.5	68.5	70.6	71.1	0	903	236
5/24/2022 10:32	0.036179	0	25.25031	25.51893	68.2	67.3	70.6	71.1	0	1501	236
5/24/2022 10:33	0.036179	0	25.25031	25.54335	67.4	66.5	70.6	71.1	0	0	236
5/24/2022 10:34	0.036179	0	25.32356	25.56776	68.3	67.6	70.8	71.3	0	0	236
5/24/2022 10:35	0.036179	0	25.27473	25.51893	71.9	71.1	70.9	71.4	0	209	236
5/24/2022 10:36	0.036179	0	25.27473	25.51893	70.4	69.4	70.8	71.4	0	808	236
5/24/2022 10:37	0.036179	0	25.27473	25.54335	68.8	67.9	70.9	71.4	0	1408	236
5/24/2022 10:38	0.036179	0	25.27473	25.54335	68	67.1	70.9	71.4	0	2017	236
5/24/2022 10:39	0.036179	0	25.27473	25.56776	67.4	66.5	70.9	71.4	0	0	236
5/24/2022 10:40	0.036179	0	25.39683	25.64103	69.6	68.9	71.1	71.6	0	0	236
5/24/2022 10:41	0.036179	0	25.32356	25.56776	72.3	71.4	71.1	71.6	0	375	236
5/24/2022 10:42	0.036179	0	25.27473	25.54335	70.4	69.4	71.1	71.6	0	982	236

5/24/2022 10:43	0.036179	0	25.27473	25.54335	69.1	68.2	71.1	71.6	0	1579	236
5/24/2022 10:44	0.036179	0	25.34798	25.56776	68.3	67.4	71.1	71.7	0	2175	236
5/24/2022 10:45	0.036179	0	25.29915	25.59219	67.8	66.8	71.2	71.7	0	0	236
5/24/2022 10:46	0.036179	0	25.34798	25.66544	67.5	66.5	71.3	71.8	0	0	236
5/24/2022 10:47	0.036179	0	25.34798	25.68987	71.4	70.5	71.4	71.9	0	66	236
5/24/2022 10:48	0.036179	0	25.27473	25.61661	71.3	70.2	71.4	71.9	0	662	236
5/24/2022 10:49	0.036179	0	25.34798	25.64103	69.8	68.6	71.4	71.9	0	1256	236
5/24/2022 10:50	0.036179	0	25.29915	25.61661	68.8	67.8	71.4	71.9	0	1865	236
5/24/2022 10:51	0.036179	0	25.32356	25.64103	68.2	67.3	71.5	72	0	2466	236
5/24/2022 10:52	0.036179	0	25.29915	25.64103	67.8	66.8	71.5	72	0	0	236
5/24/2022 10:53	0.036179	0	25.32356	25.66544	67.6	66.5	71.6	72.1	0	0	236
5/24/2022 10:54	0.036179	0	25.39683	25.71429	71.7	70.8	71.7	72.2	0	41	236
5/24/2022 10:55	0.036179	0	25.32356	25.64103	71.8	70.6	71.7	72.2	0	633	236
5/24/2022 10:56	0.036179	0	25.34798	25.64103	70.1	69	66.8	67.3	0	0	236
5/24/2022 10:57	0.953004	0.915875	25.37241	25.71429	69.9	68.9	67.8	68.3	0	0	236
5/24/2022 10:58	1.8555	1.779456	25.39683	25.81197	70	69	68.7	69.2	0	0	236
5/24/2022 10:59	2.508011	2.469324	25.42125	25.81197	70.1	69.1	69.5	70	0	0	236
5/24/2022 11:00	3.092876	3.053619	25.42125	25.81197	70.1	69	70.3	70.9	0	0	236
5/24/2022 11:01	3.686291	3.686291	25.42125	25.78754	70	68.9	71.1	71.7	0	0	236
5/24/2022 11:02	4.288256	4.247859	25.37241	25.78754	69.9	68.7	72	72.5	0	0	236
5/24/2022 11:03	4.816875	4.775984	25.34798	25.76313	69.7	68.5	72.8	73.4	0	0	236
5/24/2022 11:04	5.186604	5.145371	25.34798	25.73871	69.6	68.3	73.5	74.1	0	0	236
5/24/2022 11:05	5.145371	5.104176	25.32356	25.71429	69.4	68.1	73.8	74.3	157	0	236
5/24/2022 11:06	5.310531	5.269185	25.29915	25.68987	69.3	67.9	74.2	74.7	0	0	236
5/24/2022 11:07	5.063019	5.0219	25.25031	25.64103	69.1	67.7	74.2	74.7	511	0	236
5/24/2022 11:08	5.227875	5.186604	25.27473	25.64103	68.9	67.6	74.5	75.1	128	0	236
5/24/2022 11:09	5.063019	5.0219	25.25031	25.56776	68.7	67.4	74.5	75.1	730	0	236
5/24/2022 11:10	5.227875	5.186604	25.25031	25.51893	68.5	67.3	74.8	75.4	125	0	236
5/24/2022 11:11	5.104176	5.063019	25.20147	25.54335	68.4	67.1	74.9	75.4	713	0	236
5/24/2022 11:12	4.980819	4.939776	25.20147	25.49451	68.3	66.9	75	75.5	0	0	236
5/24/2022 11:13	5.186604	5.186604	25.17705	25.47009	68.1	66.8	75.2	75.8	344	0	236
5/24/2022 11:14	5.104176	5.063019	25.20147	25.42125	68	66.6	75.3	75.8	934	0	236
5/24/2022 11:15	5.0219	4.980819	25.17705	25.42125	67.8	66.5	75.3	75.9	1536	0	236
5/24/2022 11:16	5.269185	5.227875	25.15263	25.37241	67.7	66.3	75.6	76.2	170	0	236

5/24/2022 11:17	5.145371	5.145371	25.1282	25.34798	67.6	66.2	75.7	76.2	761	0	236
5/24/2022 11:18	5.104176	5.063019	25.1282	25.29915	67.4	66.1	75.7	76.2	1363	0	236
5/24/2022 11:19	5.0219	5.0219	25.07937	25.32356	67.3	66	75.7	76.3	1963	0	236
5/24/2022 11:20	5.269185	5.269185	25.03053	25.25031	67.1	65.9	76	76.6	12	0	236
5/24/2022 11:21	5.186604	5.145371	25.0061	25.22588	67	65.8	76	76.6	610	0	236
5/24/2022 11:22	5.104176	5.104176	24.95727	25.20147	66.9	65.7	76.1	76.6	1208	0	236
5/24/2022 11:23	5.063019	5.0219	24.95727	25.17705	66.8	65.6	76.1	76.6	1817	0	236
5/24/2022 11:24	5.0219	4.980819	24.90842	25.1282	66.6	65.5	76.1	76.7	2416	0	236
5/24/2022 11:25	5.269185	5.269185	24.90842	25.10378	66.5	65.4	76.4	77	111	0	236
5/24/2022 11:26	5.186604	5.145371	24.884	25.05495	66.4	65.3	76.5	77	699	0	236
5/24/2022 11:27	5.145371	5.104176	24.884	25.03053	66.3	65.3	76.5	77	1298	0	236
5/24/2022 11:28	5.104176	5.063019	24.884	25.0061	66.2	65.1	76.5	77.1	1896	0	236
5/24/2022 11:29	5.063019	5.0219	24.884	25.0061	66.1	65.1	76.5	77.1	2504	0	236
5/24/2022 11:30	5.0219	4.980819	24.884	24.93285	66	65	76.6	77.2	3107	0	236
5/24/2022 11:31	5.269185	5.227875	24.85959	24.93285	65.9	65	76.9	77.4	249	0	236
5/24/2022 11:32	5.186604	5.145371	24.81075	24.90842	65.7	64.9	76.9	77.5	850	0	236
5/24/2022 11:33	5.145371	5.104176	24.78632	24.884	65.7	64.9	76.9	77.5	1450	0	236
5/24/2022 11:34	5.104176	5.063019	24.76191	24.85959	65.6	64.8	77	77.5	2054	0	236
5/24/2022 11:35	5.063019	5.0219	24.71307	24.85959	65.5	64.7	77	77.5	2646	0	236
5/24/2022 11:36	5.0219	5.0219	24.71307	24.83517	65.3	64.7	77	77.6	3248	0	236
5/24/2022 11:37	5.310531	5.310531	24.66422	24.81075	65.3	64.6	77.3	77.8	0	0	236
5/24/2022 11:38	5.186604	5.186604	24.66422	24.76191	65.2	64.6	77.3	77.9	562	0	236
5/24/2022 11:39	5.145371	5.104176	24.66422	24.78632	65.1	64.5	77.3	77.9	1141	0	236
5/24/2022 11:40	5.104176	5.104176	24.63981	24.78632	65	64.5	77.4	77.9	1746	0	236
5/24/2022 11:41	5.063019	5.0219	24.61539	24.73749	64.9	64.4	77.4	78	2346	0	236
5/24/2022 11:42	5.0219	5.0219	24.61539	24.71307	64.8	64.3	77.4	78	2946	0	236
5/24/2022 11:43	5.0219	4.980819	24.59097	24.71307	64.7	64.3	77.5	78	3548	0	236
5/24/2022 11:44	5.269185	5.269185	24.59097	24.68864	64.7	64.2	77.8	78.3	256	0	236
5/24/2022 11:45	5.186604	5.186604	24.59097	24.68864	64.6	64.2	77.8	78.4	854	0	236
5/24/2022 11:46	5.145371	5.145371	24.61539	24.63981	64.5	64.2	77.8	78.4	1452	0	236
5/24/2022 11:47	5.145371	5.104176	24.61539	24.61539	64.4	64.1	77.8	78.4	2051	0	236
5/24/2022 11:48	5.104176	5.104176	24.59097	24.61539	64.3	64.1	77.9	78.5	2660	0	236
5/24/2022 11:49	5.063019	5.063019	24.59097	24.63981	64.2	64	77.9	78.5	3248	0	236
5/24/2022 11:50	5.063019	5.0219	24.59097	24.61539	64.2	64	78	78.5	3847	0	236

5/24/2022 11:51	5.0219	5.0219	24.59097	24.59097	64.1	63.9	78	78.6	4456	0	236
5/24/2022 11:52	5.310531	5.310531	24.56654	24.56654	64.1	63.9	78.3	78.9	0	0	236
5/24/2022 11:53	5.227875	5.227875	24.56654	24.56654	64	63.9	78.3	78.9	554	0	236
5/24/2022 11:54	5.186604	5.145371	24.56654	24.56654	63.9	63.8	78.3	78.9	1152	0	236
5/24/2022 11:55	5.145371	5.145371	24.51771	24.56654	63.8	63.8	78.4	78.9	1740	0	236
5/24/2022 11:56	5.104176	5.104176	24.51771	24.54212	63.8	63.7	78.4	79	2339	0	236
5/24/2022 11:57	5.104176	5.063019	24.51771	24.51771	63.7	63.6	78.5	79	2937	0	236
5/24/2022 11:58	5.063019	5.063019	24.49329	24.49329	63.6	63.7	78.5	79	3546	0	236
5/24/2022 11:59	5.0219	5.0219	24.49329	24.51771	63.5	63.6	78.5	79	4144	0	236
5/24/2022 12:00	5.0219	5.0219	24.46886	24.46886	63.5	63.6	78.6	79.1	4743	0	236
5/24/2022 12:01	5.269185	5.269185	24.44444	24.49329	63.4	63.5	78.8	79.4	0	0	236
5/24/2022 12:02	5.227875	5.227875	24.44444	24.46886	63.3	63.5	78.8	79.4	503	0	236
5/24/2022 12:03	5.186604	5.186604	24.44444	24.46886	63.3	63.5	78.9	79.4	1102	0	236
5/24/2022 12:04	5.145371	5.145371	24.39561	24.46886	63.2	63.4	78.9	79.5	1718	0	236
5/24/2022 12:05	5.104176	5.104176	24.42003	24.46886	63.2	63.4	78.9	79.5	2298	0	236
5/24/2022 12:06	5.104176	5.104176	24.34676	24.44444	63.1	63.4	79	79.5	2900	0	236
5/24/2022 12:07	5.063019	5.063019	24.39561	24.42003	63	63.3	79	79.5	3511	0	236
5/24/2022 12:08	5.063019	5.063019	24.37119	24.42003	63	63.3	79	79.6	4100	0	236
5/24/2022 12:09	5.0219	5.0219	24.39561	24.44444	63	63.3	79.1	79.6	4715	0	236
5/24/2022 12:10	5.0219	5.0219	24.37119	24.42003	62.9	63.2	79.1	79.6	5314	0	236
5/24/2022 12:11	5.393339	5.351916	24.39561	24.39561	62.9	63.2	79.4	80	0	0	236
5/24/2022 12:12	5.269185	5.227875	24.34676	24.44444	62.8	63.1	79.4	80	539	0	236
5/24/2022 12:13	5.186604	5.186604	24.37119	24.42003	62.8	63.1	79.5	80	1144	0	236
5/24/2022 12:14	5.186604	5.186604	24.37119	24.37119	62.7	63.1	79.5	80	1748	0	236
5/24/2022 12:15	5.145371	5.145371	24.37119	24.42003	62.7	63.1	79.5	80.1	2352	0	236
5/24/2022 12:16	5.145371	5.104176	24.37119	24.42003	62.6	63	79.6	80.1	2947	0	236
5/24/2022 12:17	5.104176	5.104176	24.34676	24.42003	62.6	63	79.6	80.1	3540	0	236
5/24/2022 12:18	5.104176	5.063019	24.37119	24.42003	62.5	62.9	79.6	80.2	4143	0	236
5/24/2022 12:19	5.063019	5.063019	24.34676	24.42003	62.5	62.9	79.6	80.2	4749	0	236
5/24/2022 12:20	5.063019	5.0219	24.34676	24.42003	62.5	62.9	79.7	80.2	5347	0	236
5/24/2022 12:21	5.0219	5.0219	24.34676	24.42003	62.5	62.9	79.7	80.2	5946	0	236
5/24/2022 12:22	5.0219	5.0219	24.34676	24.39561	62.3	62.8	79.7	80.3	0	0	236
5/24/2022 12:23	5.310531	5.310531	24.37119	24.42003	62.3	62.8	80	80.6	0	0	236
5/24/2022 12:24	5.227875	5.227875	24.37119	24.39561	62.3	62.8	80	80.6	0	0	236

5/24/2022 12:25	5.186604	5.186604	24.37119	24.42003	62.2	62.8	80	80.6	0	0	236
5/24/2022 12:26	5.145371	5.145371	24.37119	24.42003	62.2	62.7	80.1	80.7	0	0	236
5/24/2022 12:27	5.145371	5.104176	24.37119	24.44444	62.1	62.7	80.1	80.7	0	0	236
5/24/2022 12:28	5.104176	5.104176	24.37119	24.44444	62.1	62.7	80.1	80.7	0	0	236
5/24/2022 12:29	5.104176	5.104176	24.37119	24.42003	62.1	62.6	80.2	80.8	0	0	236
5/24/2022 12:30	5.063019	5.063019	24.37119	24.39561	62	62.6	80.2	80.8	0	0	236
5/24/2022 12:31	5.063019	5.0219	24.39561	24.44444	62	62.5	80.3	80.8	0	0	236
5/24/2022 12:32	5.0219	5.0219	24.34676	24.42003	61.9	62.5	80.3	80.9	0	0	236
5/24/2022 12:33	5.0219	5.0219	24.39561	24.42003	61.9	62.5	80.3	80.9	0	0	236
5/24/2022 12:34	5.0219	5.0219	24.39561	24.42003	61.8	62.4	80.3	80.9	0	0	236
5/24/2022 12:35	5.227875	5.227875	24.39561	24.44444	61.8	62.4	80.6	81.2	0	0	236
5/24/2022 12:36	5.186604	5.186604	24.39561	24.44444	61.8	62.4	80.6	81.2	0	0	236
5/24/2022 12:37	5.145371	5.145371	24.39561	24.46886	61.7	62.4	80.6	81.2	0	0	236
5/24/2022 12:38	5.145371	5.145371	24.39561	24.42003	61.7	62.3	80.7	81.3	0	0	236
5/24/2022 12:39	5.104176	5.104176	24.39561	24.44444	61.7	62.3	80.7	81.3	0	0	236
5/24/2022 12:40	5.104176	5.104176	24.39561	24.42003	61.6	62.3	80.8	81.3	0	0	236
5/24/2022 12:41	5.104176	5.063019	24.39561	24.46886	61.6	62.2	80.8	81.3	0	0	236
5/24/2022 12:42	5.063019	5.063019	24.37119	24.42003	61.6	62.2	80.8	81.4	0	0	236
5/24/2022 12:43	5.063019	5.063019	24.42003	24.44444	61.5	62.2	80.9	81.4	0	0	236
5/24/2022 12:44	5.0219	5.0219	24.39561	24.42003	61.5	62.1	80.9	81.4	0	0	236
5/24/2022 12:45	5.0219	5.0219	24.39561	24.44444	61.4	62.1	80.9	81.5	0	0	236
5/24/2022 12:46	5.0219	5.0219	24.37119	24.44444	61.4	62.1	80.9	81.5	0	0	236
5/24/2022 12:47	5.269185	5.269185	24.37119	24.44444	61.3	62.1	81.2	81.8	0	0	236
5/24/2022 12:48	5.227875	5.186604	24.39561	24.44444	61.4	62.1	81.2	81.8	0	0	236
5/24/2022 12:49	5.186604	5.186604	24.39561	24.44444	61.3	62	81.3	81.8	0	0	236
5/24/2022 12:50	5.145371	5.145371	24.39561	24.44444	61.3	62	81.3	81.9	0	0	236
5/24/2022 12:51	5.145371	5.145371	24.39561	24.44444	61.3	62	81.3	81.9	0	0	236
5/24/2022 12:52	5.104176	5.104176	24.39561	24.42003	61.2	62	81.4	81.9	0	0	236
5/24/2022 12:53	5.104176	5.104176	24.39561	24.44444	61.2	62	81.4	82	0	0	236
5/24/2022 12:54	5.104176	5.063019	24.39561	24.44444	61.1	61.9	81.4	82	0	0	236
5/24/2022 12:55	5.063019	5.063019	24.39561	24.44444	61.1	61.9	81.4	82	0	0	236
5/24/2022 12:56	5.063019	5.063019	24.39561	24.42003	61.1	61.9	81.5	82	0	0	236
5/24/2022 12:57	5.0219	5.0219	24.37119	24.46886	61.1	61.8	81.5	82.1	0	0	236
5/24/2022 12:58	5.0219	5.0219	24.39561	24.44444	61	61.8	81.5	82.1	0	0	236

5/24/2022 12:59	5.0219	5.0219	24.39561	24.42003	61	61.8	81.6	82.1	0	0	236
5/24/2022 13:00	5.269185	5.269185	24.42003	24.46886	61	61.8	81.8	82.4	0	0	236
5/24/2022 13:01	5.227875	5.186604	24.42003	24.42003	60.9	61.7	81.8	82.4	0	0	236
5/24/2022 13:02	5.186604	5.186604	24.39561	24.42003	60.9	61.7	81.8	82.5	0	0	236
5/24/2022 13:03	5.145371	5.145371	24.39561	24.44444	60.9	61.7	81.9	82.5	0	0	236
5/24/2022 13:04	5.145371	5.145371	24.39561	24.46886	60.8	61.6	81.9	82.5	0	0	236
5/24/2022 13:05	5.104176	5.104176	24.39561	24.46886	60.8	61.6	81.9	82.5	0	0	236
5/24/2022 13:06	5.104176	5.104176	24.39561	24.44444	60.8	61.6	82	82.5	0	0	236
5/24/2022 13:07	5.104176	5.063019	24.39561	24.42003	60.8	61.6	82	82.6	0	0	236
5/24/2022 13:08	5.063019	5.063019	24.39561	24.44444	60.7	61.5	82	82.6	0	0	236
5/24/2022 13:09	5.063019	5.063019	24.42003	24.44444	60.7	61.6	82.1	82.6	0	0	236
5/24/2022 13:10	5.0219	5.063019	24.42003	24.46886	60.7	61.5	82.1	82.6	0	0	236
5/24/2022 13:11	5.0219	5.0219	24.39561	24.46886	60.7	61.5	82.1	82.7	0	0	236
5/24/2022 13:12	5.0219	5.0219	24.39561	24.46886	60.6	61.5	82.1	82.7	0	0	236
5/24/2022 13:13	5.104176	5.104176	24.42003	24.44444	60.6	61.4	82.3	82.9	0	0	236
5/24/2022 13:14	5.227875	5.227875	24.44444	24.46886	60.6	61.4	82.5	83	0	0	236
5/24/2022 13:15	5.186604	5.186604	24.46886	24.44444	60.5	61.4	82.5	83	0	0	236
5/24/2022 13:16	5.186604	5.186604	24.44444	24.46886	60.5	61.3	82.5	83.1	0	0	236
5/24/2022 13:17	5.145371	5.145371	24.42003	24.46886	60.5	61.3	82.5	83.1	0	0	236
5/24/2022 13:18	5.145371	5.145371	24.44444	24.46886	60.4	61.3	82.6	83.1	0	0	236
5/24/2022 13:19	5.104176	5.104176	24.42003	24.46886	60.4	61.2	82.6	83.1	0	0	236
5/24/2022 13:20	5.104176	5.104176	24.44444	24.46886	60.4	61.2	82.6	83.2	0	0	236
5/24/2022 13:21	5.104176	5.104176	24.42003	24.46886	60.4	61.2	82.6	83.2	0	0	236
5/24/2022 13:22	5.104176	5.063019	24.39561	24.46886	60.3	61.2	82.7	83.3	0	0	236
5/24/2022 13:23	5.063019	5.063019	24.42003	24.46886	60.3	61.1	82.7	83.3	0	0	236
5/24/2022 13:24	5.063019	5.063019	24.39561	24.44444	60.3	61.2	82.7	83.3	0	0	236
5/24/2022 13:25	5.0219	5.0219	24.39561	24.46886	60.2	61.1	82.7	83.3	0	0	236
5/24/2022 13:26	5.0219	5.0219	24.39561	24.44444	60.2	61.1	82.7	83.4	0	0	236
5/24/2022 13:27	5.0219	5.0219	24.39561	24.44444	60.2	61.1	82.8	83.4	0	0	236
5/24/2022 13:28	4.980819	5.0219	24.39561	24.46886	60.1	61.1	82.8	83.4	0	0	236
5/24/2022 13:29	5.227875	5.227875	24.39561	24.44444	60.2	61	83.1	83.7	0	0	236
5/24/2022 13:30	5.186604	5.186604	24.39561	24.44444	60.1	61	83.1	83.7	0	0	236
5/24/2022 13:31	5.145371	5.145371	24.39561	24.44444	60.1	61	83.1	83.7	0	0	236
5/24/2022 13:32	5.145371	5.145371	24.37119	24.44444	60.1	61	83.1	83.7	0	0	236

5/24/2022 13:33	5.145371	5.104176	24.34676	24.46886	60	60.9	83.2	83.8	0	0	236
5/24/2022 13:34	5.104176	5.104176	24.34676	24.46886	60	61	83.2	83.8	0	0	236
5/24/2022 13:35	5.104176	5.104176	24.34676	24.44444	60	60.9	83.2	83.8	0	0	236
5/24/2022 13:36	5.104176	5.104176	24.37119	24.42003	60	60.9	83.3	83.9	0	0	236
5/24/2022 13:37	5.063019	5.063019	24.34676	24.44444	60	60.9	83.3	83.9	0	0	236
5/24/2022 13:38	5.063019	5.063019	24.34676	24.44444	60	60.9	83.3	83.9	0	0	236
5/24/2022 13:39	5.0219	5.0219	24.34676	24.44444	59.9	60.8	83.4	83.9	0	0	236
5/24/2022 13:40	5.0219	5.0219	24.34676	24.39561	59.9	60.8	83.4	84	0	0	236
5/24/2022 13:41	5.0219	5.0219	24.32234	24.42003	59.9	60.8	83.4	84	0	0	236
5/24/2022 13:42	5.0219	5.0219	24.32234	24.42003	59.8	60.8	83.4	84	0	0	236
5/24/2022 13:43	5.269185	5.269185	24.32234	24.42003	59.8	60.8	83.7	84.3	0	0	236
5/24/2022 13:44	5.227875	5.227875	24.32234	24.42003	59.8	60.7	83.7	84.3	0	0	236
5/24/2022 13:45	5.186604	5.186604	24.32234	24.42003	59.7	60.7	83.8	84.3	0	0	236
5/24/2022 13:46	5.186604	5.186604	24.32234	24.39561	59.7	60.7	83.8	84.3	0	0	236
5/24/2022 13:47	5.145371	5.145371	24.32234	24.42003	59.7	60.7	83.8	84.4	0	0	236
5/24/2022 13:48	5.145371	5.145371	24.32234	24.37119	59.7	60.7	83.8	84.4	0	0	236
5/24/2022 13:49	5.104176	5.145371	24.32234	24.39561	59.7	60.6	83.8	84.4	0	0	236
5/24/2022 13:50	5.104176	5.104176	24.29793	24.39561	59.7	60.6	83.9	84.4	0	0	236
5/24/2022 13:51	5.104176	5.104176	24.32234	24.37119	59.7	60.6	83.9	84.5	0	0	236
5/24/2022 13:52	5.104176	5.104176	24.32234	24.37119	59.6	60.6	83.9	84.5	0	0	236
5/24/2022 13:53	5.104176	5.063019	24.32234	24.42003	59.7	60.6	83.9	84.5	0	0	236
5/24/2022 13:54	5.063019	5.063019	24.32234	24.37119	59.6	60.5	84	84.6	0	0	236
5/24/2022 13:55	5.063019	5.063019	24.32234	24.39561	59.6	60.5	84	84.6	0	0	236
5/24/2022 13:56	5.0219	5.063019	24.32234	24.37119	59.6	60.5	84	84.6	0	0	236
5/24/2022 13:57	5.0219	5.0219	24.32234	24.39561	59.6	60.5	84	84.7	0	0	236
5/24/2022 13:58	5.0219	5.0219	24.32234	24.39561	59.5	60.5	84.1	84.7	0	0	236
5/24/2022 13:59	5.0219	5.0219	24.32234	24.39561	59.5	60.5	84.1	84.7	0	0	236
5/24/2022 14:00	5.145371	5.145371	24.29793	24.37119	59.5	60.4	84.3	84.9	0	0	236
5/24/2022 14:01	5.227875	5.227875	24.32234	24.39561	59.5	60.4	84.4	85	0	0	236
5/24/2022 14:02	5.186604	5.186604	24.32234	24.39561	59.5	60.4	84.4	85	0	0	236
5/24/2022 14:03	5.186604	5.186604	24.32234	24.37119	59.5	60.4	84.5	85	0	0	236
5/24/2022 14:04	5.145371	5.145371	24.32234	24.39561	59.4	60.3	84.5	85.1	0	0	236
5/24/2022 14:05	5.145371	5.145371	24.32234	24.37119	59.4	60.3	84.5	85.1	0	0	236
5/24/2022 14:06	5.104176	5.104176	24.32234	24.37119	59.4	60.3	84.5	85.1	0	0	236

5/24/2022 14:07	5.104176	5.104176	24.32234	24.34676	59.4	60.3	84.5	85.1	0	0	236
5/24/2022 14:08	5.104176	5.104176	24.32234	24.34676	59.3	60.3	84.6	85.2	0	0	236
5/24/2022 14:09	5.104176	5.104176	24.29793	24.32234	59.3	60.3	84.6	85.2	0	0	236
5/24/2022 14:10	5.104176	5.063019	24.27351	24.32234	59.3	60.2	84.6	85.2	0	0	236
5/24/2022 14:11	5.063019	5.063019	24.24908	24.29793	59.3	60.2	84.6	85.2	0	0	236
5/24/2022 14:12	5.063019	5.063019	24.22466	24.29793	59.3	60.2	84.7	85.2	0	0	236
5/24/2022 14:13	5.063019	5.063019	24.20025	24.29793	59.3	60.2	84.7	85.3	0	0	236
5/24/2022 14:14	5.0219	5.0219	24.15141	24.29793	59.3	60.2	84.7	85.3	0	0	236
5/24/2022 14:15	5.0219	5.0219	24.15141	24.24908	59.2	60.1	84.7	85.3	0	0	236
5/24/2022 14:16	5.0219	5.0219	24.15141	24.24908	59.3	60.2	84.7	85.3	0	0	236
5/24/2022 14:17	5.0219	5.0219	24.10256	24.24908	59.2	60.1	84.8	85.4	0	0	236
5/24/2022 14:18	5.269185	5.269185	24.15141	24.22466	59.2	60.2	85	85.6	0	0	236
5/24/2022 14:19	5.227875	5.227875	24.12698	24.22466	59.2	60.1	85.1	85.6	0	0	236
5/24/2022 14:20	5.186604	5.186604	24.10256	24.22466	59.3	60.1	85.1	85.7	0	0	236
5/24/2022 14:21	5.186604	5.186604	24.07815	24.22466	59.2	60.1	85.1	85.7	0	0	236
5/24/2022 14:22	5.145371	5.145371	24.07815	24.22466	59.2	60.1	85.2	85.8	0	0	236
5/24/2022 14:23	5.145371	5.145371	24.07815	24.20025	59.1	60.1	85.2	85.8	0	0	236
5/24/2022 14:24	5.145371	5.145371	24.12698	24.22466	59.2	60.1	85.2	85.8	0	0	236
5/24/2022 14:25	5.104176	5.104176	24.10256	24.22466	59.2	60	85.2	85.8	0	0	236
5/24/2022 14:26	5.104176	5.104176	24.07815	24.22466	59.1	60.1	85.2	85.9	0	0	236
5/24/2022 14:27	5.104176	5.104176	24.10256	24.20025	59.1	60	85.3	85.9	0	0	236
5/24/2022 14:28	5.104176	5.104176	24.10256	24.20025	59.1	60	85.3	85.9	0	0	236
5/24/2022 14:29	5.104176	5.063019	24.07815	24.17583	59.1	59.9	85.3	85.9	0	0	236
5/24/2022 14:30	5.063019	5.063019	24.05373	24.20025	59	60	85.4	86	0	0	236
5/24/2022 14:31	5.063019	5.063019	24.05373	24.17583	59	60	85.4	86	0	0	236
5/24/2022 14:32	5.063019	5.0219	24.0293	24.17583	59	59.9	85.4	86	0	0	236
5/24/2022 14:33	5.0219	5.0219	24.0293	24.15141	59	59.9	85.4	86	0	0	236
5/24/2022 14:34	5.0219	5.0219	24.05373	24.15141	59	59.9	85.4	86	0	0	236
5/24/2022 14:35	5.0219	5.0219	24.0293	24.15141	59	59.9	85.5	86.1	0	0	236
5/24/2022 14:36	5.0219	5.0219	24.0293	24.15141	59	59.9	85.5	86.1	0	0	236
5/24/2022 14:37	5.145371	5.145371	24.0293	24.12698	59	59.9	85.7	86.3	0	0	236
5/24/2022 14:38	5.227875	5.227875	24.0293	24.15141	59	59.9	85.8	86.4	0	0	236
5/24/2022 14:39	5.186604	5.186604	24.0293	24.15141	59	59.9	85.8	86.4	0	0	236
5/24/2022 14:40	5.186604	5.186604	24.0293	24.12698	59	59.8	85.8	86.4	0	0	236

5/24/2022 14:41	5.145371	5.145371	24.0293	24.12698	58.9	59.9	85.9	86.4	0	0	236
5/24/2022 14:42	5.145371	5.145371	24.0293	24.12698	58.9	59.8	85.9	86.5	0	0	236
5/24/2022 14:43	5.145371	5.145371	24.00488	24.10256	58.9	59.8	85.9	86.5	0	0	236
5/24/2022 14:44	5.104176	5.104176	24.05373	24.12698	58.9	59.8	85.9	86.5	0	0	236
5/24/2022 14:45	5.104176	5.104176	24.00488	24.12698	58.9	59.8	85.9	86.6	0	0	236
5/24/2022 14:46	5.104176	5.104176	24.05373	24.12698	58.9	59.8	86	86.6	0	0	236
5/24/2022 14:47	5.104176	5.104176	24.0293	24.12698	58.9	59.8	86	86.6	0	0	236
5/24/2022 14:48	5.104176	5.063019	24.0293	24.12698	58.9	59.7	86	86.6	0	0	236
5/24/2022 14:49	5.063019	5.063019	24.05373	24.10256	58.9	59.7	86	86.6	0	0	236
5/24/2022 14:50	5.063019	5.063019	24.0293	24.12698	58.8	59.7	86	86.6	0	0	236
5/24/2022 14:51	5.063019	5.063019	24.00488	24.10256	58.8	59.7	86.1	86.7	0	0	236
5/24/2022 14:52	5.0219	5.0219	24.0293	24.15141	58.7	59.6	86.1	86.7	0	0	236
5/24/2022 14:53	5.0219	5.0219	24.0293	24.05373	58.8	59.6	86.1	86.7	0	0	236
5/24/2022 14:54	5.0219	5.0219	24.00488	24.05373	58.8	59.6	86.2	86.7	0	0	236
5/24/2022 14:55	5.0219	5.0219	24.00488	24.05373	58.7	59.6	86.2	86.8	0	0	236
5/24/2022 14:56	5.0219	5.0219	23.95604	24.05373	58.7	59.5	86.2	86.8	0	0	236
5/24/2022 14:57	5.269185	5.269185	23.98046	24.07815	58.8	59.6	86.5	87	0	0	236
5/24/2022 14:58	5.227875	5.227875	23.95604	24.05373	58.7	59.6	86.5	87.1	0	0	236
5/24/2022 14:59	5.186604	5.186604	23.95604	24.05373	58.7	59.6	86.5	87.1	0	0	236
5/24/2022 15:00	5.186604	5.186604	23.93163	24.0293	58.7	59.5	86.5	87.1	0	0	236
5/24/2022 15:01	5.186604	5.145371	23.93163	24.00488	58.7	59.6	86.5	87.1	0	0	236
5/24/2022 15:02	5.145371	5.145371	23.95604	24.0293	58.7	59.5	86.5	87.2	0	0	236
5/24/2022 15:03	5.145371	5.145371	23.98046	24.0293	58.7	59.6	86.6	87.2	0	0	236
5/24/2022 15:04	5.145371	5.145371	23.95604	24.0293	58.7	59.5	86.6	87.2	0	0	236
5/24/2022 15:05	5.104176	5.104176	23.93163	24.00488	58.7	59.5	86.6	87.2	0	0	236
5/24/2022 15:06	5.104176	5.104176	23.95604	24.0293	58.6	59.5	86.7	87.2	0	0	236
5/24/2022 15:07	5.104176	5.104176	24.00488	24.0293	58.6	59.5	86.7	87.3	0	0	236
5/24/2022 15:08	5.104176	5.104176	23.95604	24.05373	58.6	59.5	86.7	87.3	0	0	236
5/24/2022 15:09	5.104176	5.063019	23.98046	24.0293	58.6	59.4	86.7	87.3	0	0	236
5/24/2022 15:10	5.063019	5.063019	23.95604	24.05373	58.6	59.4	86.7	87.3	0	0	236
5/24/2022 15:11	5.063019	5.063019	24.00488	24.05373	58.5	59.4	86.7	87.3	0	0	236
5/24/2022 15:12	5.063019	5.063019	23.93163	24.0293	58.5	59.4	86.8	87.4	0	0	236
5/24/2022 15:13	5.0219	5.0219	23.88279	24.00488	58.4	59.3	86.8	87.4	0	0	236
5/24/2022 15:14	5.0219	5.0219	23.85836	23.98046	58.5	59.4	86.8	87.4	0	0	236

5/24/2022 15:15	5.0219	5.0219	23.80952	23.98046	58.4	59.3	86.8	87.4	0	0	236
5/24/2022 15:16	5.0219	5.0219	23.80952	23.98046	58.5	59.3	86.8	87.4	0	0	236
5/24/2022 15:17	5.0219	5.0219	23.80952	23.98046	58.4	59.3	86.8	87.4	0	0	236
5/24/2022 15:18	5.393339	5.393339	23.80952	23.95604	58.4	59.3	87.1	87.7	0	0	236
5/24/2022 15:19	5.063019	5.063019	23.78511	23.95604	58.4	59.3	83.3	83.9	0	0	236
5/24/2022 15:20	2.975219	2.975219	23.80952	23.93163	58.5	59.3	46.3	46.6	0	0	236
5/24/2022 15:21	1.817459	1.817459	23.76068	23.93163	58.5	59.3	26.3	26.5	0	0	236
5/24/2022 15:22	1.064619	1.064619	23.80952	23.93163	58.5	59.2	67.1	67.6	0	0	236
5/24/2022 15:23	0.878784	0.878784	23.78511	23.95604	58.4	59.3	37.4	37.7	0	0	236
5/24/2022 15:24	0.7308	0.7308	23.80952	23.9072	58.5	59.2	65.8	66.2	0	0	236
5/24/2022 15:25	0.436656	0.473291	23.85836	23.98046	58.4	59.3	54.2	54.6	0	0	236
5/24/2022 15:26	0.3635	0.400059	23.83395	23.95604	58.4	59.2	30.4	30.7	0	0	236
5/24/2022 15:27	0.217644	0.254051	23.85836	23.98046	58.4	59.3	82.7	83.3	0	0	236
5/24/2022 15:28	0.217644	0.254051	23.88279	23.98046	58.4	59.2	45.8	46.2	0	0	236
5/24/2022 15:29	0.217644	0.217644	23.85836	23.98046	58.4	59.2	64.2	64.7	0	0	236
5/24/2022 15:30	0.144944	0.144944	23.88279	23.98046	58.3	59.2	54.5	54.9	0	0	236
5/24/2022 15:31	0.108651	0.144944	23.85836	23.98046	58.3	59.1	30.3	30.6	0	0	236
5/24/2022 15:32	0.072396	0.108651	23.85836	23.98046	58.3	59.1	64.3	64.8	0	0	236
5/24/2022 15:33	0.072396	0.108651	23.83395	23.98046	58.2	59.1	35.9	36.2	0	0	236
5/24/2022 15:34	0.072396	0.072396	23.83395	23.95604	58.2	59.1	76.8	77.3	0	0	236
5/24/2022 15:35	0.072396	0.072396	23.78511	23.95604	58.3	59.1	42.6	43	0	0	236
5/24/2022 15:36	0.072396	0.072396	23.78511	23.93163	58.2	59.1	82.9	83.5	0	0	236
5/24/2022 15:37	0.036179	0.072396	23.76068	23.93163	58.3	59	50.8	51.2	0	0	236
5/24/2022 15:38	0.036179	0.072396	23.76068	23.93163	58.2	59	32.8	33	0	0	236
5/24/2022 15:39	0.036179	0.072396	23.78511	23.93163	58.3	59	59.9	60.4	0	0	236
5/24/2022 15:40	0.036179	0.072396	23.78511	23.9072	58.2	59.1	33.6	33.9	0	0	236
5/24/2022 15:41	0.036179	0.072396	23.78511	23.9072	58.2	59	71.8	72.3	0	0	236
5/24/2022 15:42	0.036179	0.072396	23.85836	23.9072	58.2	59	40	40.3	0	0	236
5/24/2022 15:43	0.036179	0.072396	23.78511	23.93163	58.2	59	86.4	87.1	0	0	236
5/24/2022 15:44	0	0.072396	23.83395	23.93163	58.2	59	47.6	48	0	0	236
5/24/2022 15:45	0.036179	0.072396	23.85836	23.93163	58.2	59	52.1	52.6	0	0	236

Run 237

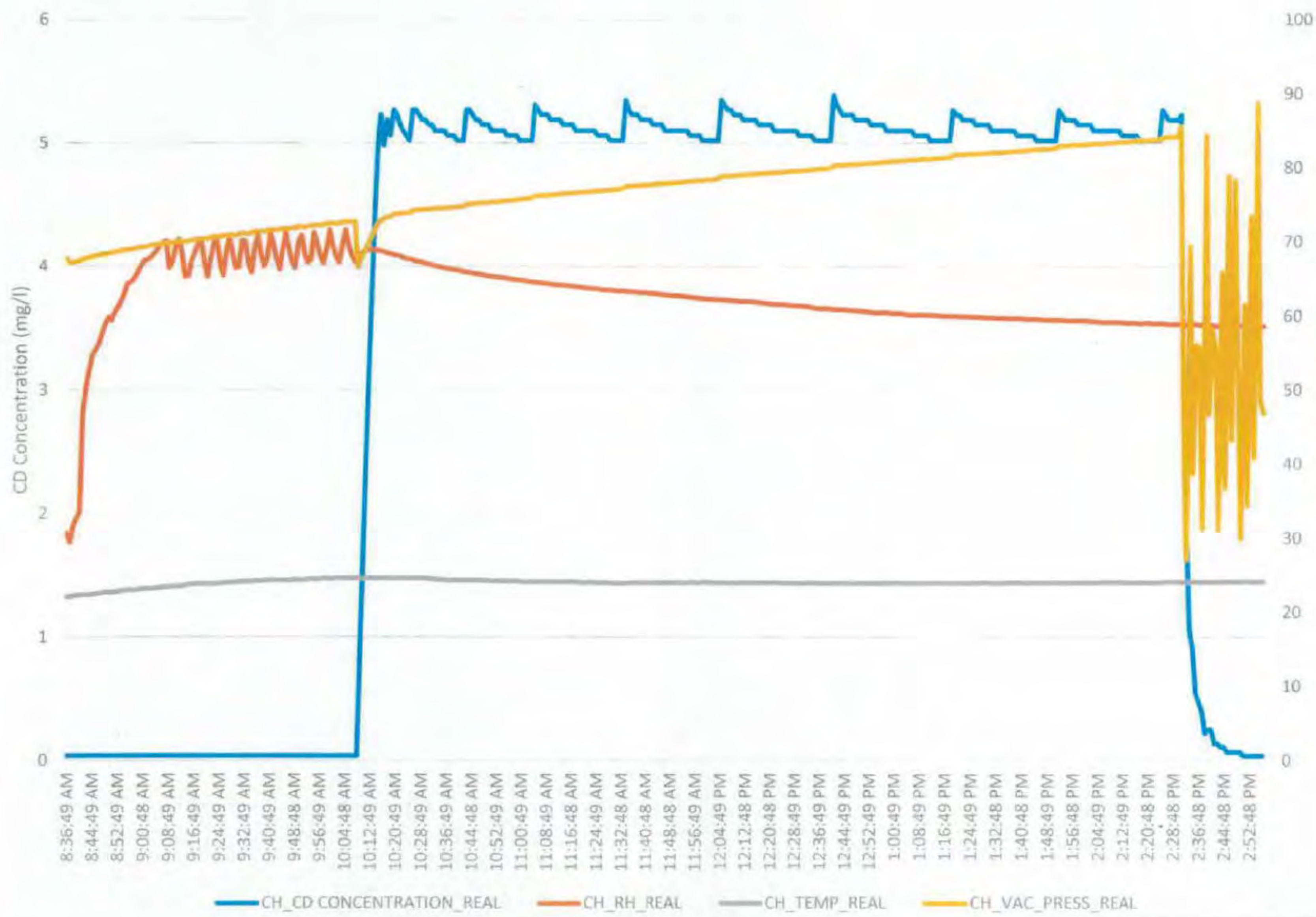
CD Concentration (mg/l)
RH, Temperature (°F), Pressure
0
1
2
3
4
5
6
0
10
20
30
40
50
60
70
80
90
100
8:36:49 AM
8:44:49 AM
8:52:49 AM
9:00:48 AM
9:08:49 AM
9:16:49 AM
9:24:49 AM
9:32:49 AM
9:40:49 AM
9:48:48 AM
9:56:49 AM
10:04:48 AM
10:12:49 AM
10:20:49 AM
10:28:49 AM
10:36:49 AM
10:44:48 AM
10:52:49 AM
11:00:49 AM
11:08:49 AM
11:16:48 AM
11:24:49 AM
11:32:48 AM
11:40:48 AM
11:48:48 AM
11:56:49 AM
12:04:49 PM
12:12:48 PM
12:20:48 PM
12:28:49 PM
12:36:49 PM
12:44:49 PM
12:52:49 PM
1:00:49 PM
1:08:49 PM
1:16:49 PM
1:24:49 PM
1:32:48 PM
1:40:48 PM
1:48:49 PM
1:56:48 PM
2:04:49 PM
2:12:49 PM
2:20:48 PM
2:28:48 PM
2:36:48 PM
2:44:48 PM
2:52:48 PM
CH_CD CONCENTRATION_REAL
CH_RH_REAL
CH_TEMP_REAL
CH_VAC_PRESS_REAL

Date	CH_CD CONCENTRATION_REAL	CH_RH_REAL	CH_TEMP_REAL	CH_VAC_PRESS_REAL	PPM-HRS_REAL
8:36:49 AM	0.039999999	30.70000076	22.10000038	67.69999695	0
8:37:49 AM	0.039999999	29.5	22.10000038	67.09999847	0
8:38:49 AM	0.039999999	31.70000076	22.20000076	67.19999695	0
8:39:49 AM	0.039999999	32.70000076	22.20000076	67.30000305	0
8:40:49 AM	0.039999999	33.40000153	22.29999924	67.40000153	0
8:41:49 AM	0.039999999	46.90000153	22.29999924	67.69999695	0
8:42:49 AM	0.039999999	50.5	22.29999924	67.80000305	0
8:43:49 AM	0.039999999	52.70000076	22.29999924	67.90000153	0
8:44:49 AM	0.039999999	54.70000076	22.39999962	68	0
8:45:49 AM	0.039999999	55.5	22.39999962	68.09999847	0
8:46:49 AM	0.039999999	56.20000076	22.5	68.19999695	0
8:47:49 AM	0.039999999	57.70000076	22.60000038	68.30000305	0
8:48:49 AM	0.039999999	58.90000153	22.70000076	68.40000153	0
8:49:49 AM	0.039999999	59.79999924	22.70000076	68.5	0
8:50:49 AM	0.039999999	59.40000153	22.70000076	68.5	0
8:51:49 AM	0.039999999	60.5	22.70000076	68.59999847	0
8:52:49 AM	0.039999999	61.20000076	22.79999924	68.69999695	0
8:53:49 AM	0.039999999	62	22.89999962	68.80000305	0
8:54:48 AM	0.039999999	63	22.89999962	68.90000153	0
8:55:49 AM	0.039999999	64.30000305	23	68.90000153	0
8:56:49 AM	0.039999999	64.59999847	23	69	0
8:57:49 AM	0.039999999	65	23	69.09999847	0
8:58:49 AM	0.039999999	65.69999695	23	69.19999695	0
8:59:49 AM	0.039999999	66.5	23.10000038	69.30000305	0
9:00:48 AM	0.039999999	67.30000305	23.20000076	69.30000305	0
9:01:49 AM	0.039999999	67.59999847	23.20000076	69.40000153	0
9:02:48 AM	0.039999999	67.80000305	23.20000076	69.5	0
9:03:48 AM	0.039999999	68.30000305	23.20000076	69.59999847	0
9:04:49 AM	0.039999999	68.80000305	23.29999924	69.59999847	0
9:05:48 AM	0.039999999	69.5	23.29999924	69.69999695	0
9:06:49 AM	0.039999999	69.90000153	23.39999962	69.80000305	0
9:07:48 AM	0.039999999	70.09999847	23.39999962	69.80000305	0
9:08:49 AM	0.039999999	66.5	23.39999962	69.80000305	0

9:09:48 AM	0.039999999	67.30000305	23.5	69.90000153	0
9:10:49 AM	0.039999999	69.40000153	23.5	70	0
9:11:49 AM	0.039999999	70.40000153	23.5	70.09999847	0
9:12:48 AM	0.039999999	68.5	23.5	70	0
9:13:49 AM	0.039999999	65.30000305	23.60000038	70	0
9:14:48 AM	0.039999999	65.40000153	23.70000076	70.09999847	0
9:15:49 AM	0.039999999	67.30000305	23.70000076	70.19999695	0
9:16:49 AM	0.039999999	68.59999847	23.79999924	70.30000305	0
9:17:49 AM	0.039999999	69.59999847	23.79999924	70.40000153	0
9:18:48 AM	0.039999999	70.40000153	23.79999924	70.40000153	0
9:19:48 AM	0.039999999	67.40000153	23.79999924	70.40000153	0
9:20:48 AM	0.039999999	65.30000305	23.79999924	70.5	0
9:21:49 AM	0.039999999	67.69999695	23.79999924	70.59999847	0
9:22:49 AM	0.039999999	69.90000153	23.79999924	70.69999695	0
9:23:49 AM	0.039999999	70.30000305	23.79999924	70.69999695	0
9:24:49 AM	0.039999999	67.19999695	23.89999962	70.69999695	0
9:25:49 AM	0.039999999	65.5	23.89999962	70.80000305	0
9:26:49 AM	0.039999999	68.69999695	24	70.90000153	0
9:27:49 AM	0.039999999	70.90000153	24	70.90000153	0
9:28:49 AM	0.039999999	68.5	24	70.90000153	0
9:29:48 AM	0.039999999	66.5	24	70.90000153	0
9:30:48 AM	0.039999999	66.59999847	24.10000038	71.09999847	0
9:31:48 AM	0.039999999	70.19999695	24.10000038	71.19999695	0
9:32:49 AM	0.039999999	70.09999847	24.10000038	71.09999847	0
9:33:48 AM	0.039999999	67.30000305	24.10000038	71.09999847	0
9:34:49 AM	0.039999999	65.90000153	24.10000038	71.19999695	0
9:35:48 AM	0.039999999	69.09999847	24.20000076	71.40000153	0
9:36:49 AM	0.039999999	70.90000153	24.20000076	71.40000153	0
9:37:49 AM	0.039999999	68.5	24.20000076	71.40000153	0
9:38:49 AM	0.039999999	66.69999695	24.20000076	71.40000153	0
9:39:49 AM	0.039999999	67.59999847	24.29999924	71.5	0
9:40:49 AM	0.039999999	71.19999695	24.29999924	71.59999847	0
9:41:49 AM	0.039999999	69.59999847	24.29999924	71.59999847	0
9:42:49 AM	0.039999999	67.59999847	24.29999924	71.59999847	0

9:43:49 AM	0.039999999	66.30000305	24.29999924	71.69999695	0
9:44:49 AM	0.039999999	69.09999847	24.29999924	71.80000305	0
9:45:48 AM	0.039999999	71.30000305	24.29999924	71.80000305	0
9:46:48 AM	0.039999999	69.09999847	24.29999924	71.80000305	0
9:47:48 AM	0.039999999	67.30000305	24.29999924	71.90000153	0
9:48:48 AM	0.039999999	66.5	24.39999962	71.90000153	0
9:49:48 AM	0.039999999	70	24.39999962	72.09999847	0
9:50:48 AM	0.039999999	70.90000153	24.29999924	72	0
9:51:49 AM	0.039999999	68.5	24.39999962	72	0
9:52:49 AM	0.039999999	67.19999695	24.39999962	72.09999847	0
9:53:48 AM	0.039999999	67.59999847	24.5	72.19999695	0
9:54:48 AM	0.039999999	71.30000305	24.5	72.30000305	0
9:55:48 AM	0.039999999	69.90000153	24.39999962	72.30000305	0
9:56:49 AM	0.039999999	68.09999847	24.5	72.30000305	0
9:57:49 AM	0.039999999	67.09999847	24.5	72.30000305	0
9:58:49 AM	0.039999999	69.09999847	24.60000038	72.5	0
9:59:49 AM	0.039999999	71.69999695	24.5	72.5	0
10:00:49 AM	0.039999999	69.5	24.5	72.5	0
10:01:49 AM	0.039999999	68	24.60000038	72.5	0
10:02:48 AM	0.039999999	67.19999695	24.60000038	72.5	0
10:03:48 AM	0.039999999	69.5	24.60000038	72.69999695	0
10:04:48 AM	0.039999999	71.69999695	24.60000038	72.69999695	0
10:05:49 AM	0.039999999	69.5	24.60000038	72.69999695	0
10:06:49 AM	0.039999999	68.19999695	24.60000038	72.69999695	0
10:07:48 AM	0.039999999	67.30000305	24.60000038	72.69999695	0
10:08:48 AM	0.039999999	68.30000305	24.60000038	66.59999847	0
10:09:49 AM	1.029999971	68.5	24.60000038	67.90000153	5.731666565
10:10:49 AM	1.970000029	68.80000305	24.60000038	68.80000305	17.13466644
10:11:49 AM	2.819999933	68.90000153	24.60000038	69.69999695	33.66600037
10:12:49 AM	3.569999933	69	24.60000038	70.59999847	55.20500183
10:13:49 AM	4.289999962	68.90000153	24.60000038	71.5	80.60533142
10:14:49 AM	4.940000057	68.90000153	24.60000038	72.40000153	110.168663
10:15:49 AM	5.230000019	68.80000305	24.60000038	72.90000153	141.964325
10:16:48 AM	4.980000019	68.69999695	24.60000038	73.09999847	172.0103302

10:17:48 AM	5.190000057	68.5	24.60000038	73.40000153	203.5646667
10:18:49 AM	5.059999943	68.40000153	24.60000038	73.40000153	234.093338
10:19:49 AM	5.269999981	68.30000305	24.60000038	73.80000305	265.6476746
10:20:49 AM	5.230000019	68.19999695	24.60000038	73.80000305	297.2019958
10:21:49 AM	5.150000095	68.09999847	24.60000038	73.80000305	328.2736511
10:22:49 AM	5.099999905	67.90000153	24.60000038	73.90000153	359.0436401
10:23:48 AM	5.059999943	67.80000305	24.60000038	73.90000153	389.5722961
10:24:48 AM	5.019999981	67.69999695	24.60000038	73.90000153	419.8596191
10:25:48 AM	5.269999981	67.59999847	24.60000038	74.19999695	451.1726074
10:26:48 AM	5.269999981	67.40000153	24.60000038	74.30000305	482.9682617
10:27:49 AM	5.230000019	67.30000305	24.60000038	74.30000305	514.522583
10:28:49 AM	5.190000057	67.30000305	24.60000038	74.40000153	545.8355713
10:29:49 AM	5.190000057	67.09999847	24.60000038	74.40000153	577.1485596
10:30:49 AM	5.150000095	67	24.5	74.40000153	608.2202148
10:31:48 AM	5.150000095	66.90000153	24.5	74.5	639.2918701
10:32:48 AM	5.099999905	66.80000305	24.5	74.5	670.0618896
10:33:49 AM	5.099999905	66.69999695	24.39999962	74.59999847	700.8319092
10:34:48 AM	5.099999905	66.59999847	24.39999962	74.59999847	731.6019287
10:35:49 AM	5.099999905	66.5	24.39999962	74.59999847	762.3719482
10:36:49 AM	5.059999943	66.5	24.29999924	74.59999847	792.9006348
10:37:49 AM	5.059999943	66.30000305	24.29999924	74.69999695	823.4293213
10:38:49 AM	5.059999943	66.30000305	24.29999924	74.69999695	853.9580078
10:39:49 AM	5.019999981	66.19999695	24.29999924	74.69999695	884.2453613
10:40:49 AM	5.019999981	66.19999695	24.29999924	74.80000305	914.5327148
10:41:48 AM	5.019999981	66	24.29999924	74.80000305	944.8200684
10:42:48 AM	5.269999981	66	24.29999924	75.09999847	975.8917236
10:43:48 AM	5.269999981	65.90000153	24.29999924	75.09999847	1007.446045
10:44:48 AM	5.230000019	65.80000305	24.29999924	75.19999695	1039.000366
10:45:49 AM	5.190000057	65.69999695	24.29999924	75.19999695	1070.313354
10:46:49 AM	5.190000057	65.69999695	24.29999924	75.30000305	1101.626343
10:47:49 AM	5.150000095	65.59999847	24.29999924	75.30000305	1132.697998
10:48:49 AM	5.150000095	65.5	24.29999924	75.30000305	1163.769653
10:49:49 AM	5.150000095	65.40000153	24.29999924	75.30000305	1194.841309
10:50:49 AM	5.099999905	65.40000153	24.20000076	75.40000153	1225.611328

10:51:49 AM	5.099999905	65.30000305	24.20000076	75.40000153	1256.381348
10:52:49 AM	5.099999905	65.30000305	24.20000076	75.40000153	1287.151367
10:53:49 AM	5.099999905	65.19999695	24.20000076	75.5	1317.921387
10:54:49 AM	5.099999905	65.19999695	24.20000076	75.5	1348.691406
10:55:49 AM	5.059999943	65.09999847	24.20000076	75.59999847	1379.220093
10:56:49 AM	5.059999943	65	24.20000076	75.59999847	1409.748779
10:57:48 AM	5.059999943	65	24.20000076	75.59999847	1440.277466
10:58:48 AM	5.059999943	64.90000153	24.20000076	75.69999695	1470.806152
10:59:49 AM	5.019999981	64.90000153	24.20000076	75.69999695	1501.093506
11:00:49 AM	5.019999981	64.80000305	24.10000038	75.80000305	1531.380859
11:01:49 AM	5.019999981	64.80000305	24.10000038	75.80000305	1561.668213
11:02:49 AM	5.019999981	64.69999695	24.10000038	75.80000305	1591.955566
11:03:49 AM	5.019999981	64.59999847	24.10000038	76	1622.24292
11:04:49 AM	5.309999943	64.59999847	24.10000038	76.19999695	1654.279907
11:05:48 AM	5.269999981	64.5	24.10000038	76.30000305	1686.075562
11:06:48 AM	5.230000019	64.5	24.10000038	76.30000305	1717.629883
11:07:49 AM	5.230000019	64.40000153	24.10000038	76.30000305	1749.184204
11:08:49 AM	5.230000019	64.40000153	24.10000038	76.30000305	1780.738525
11:09:49 AM	5.190000057	64.30000305	24.10000038	76.40000153	1812.051514
11:10:48 AM	5.190000057	64.30000305	24.10000038	76.40000153	1843.364502
11:11:48 AM	5.190000057	64.19999695	24.10000038	76.40000153	1874.67749
11:12:49 AM	5.190000057	64.19999695	24.10000038	76.5	1905.990479
11:13:49 AM	5.150000095	64.19999695	24.10000038	76.5	1937.062134
11:14:48 AM	5.150000095	64.09999847	24.10000038	76.59999847	1968.133789
11:15:48 AM	5.150000095	64.09999847	24.10000038	76.59999847	1999.205444
11:16:48 AM	5.150000095	64	24.10000038	76.59999847	2030.2771
11:17:49 AM	5.099999905	64	24.10000038	76.59999847	2061.047119
11:18:49 AM	5.099999905	63.90000153	24.10000038	76.69999695	2091.817139
11:19:49 AM	5.099999905	63.90000153	24	76.69999695	2122.587158
11:20:49 AM	5.099999905	63.79999924	24	76.69999695	2153.357178
11:21:49 AM	5.099999905	63.79999924	24	76.80000305	2184.127197
11:22:49 AM	5.099999905	63.70000076	24.10000038	76.80000305	2214.897217
11:23:49 AM	5.099999905	63.70000076	24	76.90000153	2245.667236
11:24:49 AM	5.059999943	63.70000076	24	76.90000153	2276.195801

11:25:49 AM	5.059999943	63.59999847	24	76.90000153	2306.724365
11:26:48 AM	5.059999943	63.59999847	24	76.90000153	2337.25293
11:27:48 AM	5.059999943	63.5	24	77	2367.781494
11:28:48 AM	5.019999981	63.59999847	24	77	2398.068848
11:29:49 AM	5.019999981	63.5	23.89999962	77.09999847	2428.356201
11:30:49 AM	5.019999981	63.40000153	23.89999962	77.09999847	2458.643555
11:31:48 AM	5.019999981	63.40000153	23.89999962	77.09999847	2488.930908
11:32:48 AM	5.019999981	63.40000153	23.89999962	77.19999695	2519.218262
11:33:49 AM	5.349999905	63.40000153	23.89999962	77.5	2551.496582
11:34:49 AM	5.269999981	63.29999924	23.89999962	77.5	2583.292236
11:35:49 AM	5.230000019	63.29999924	24	77.59999847	2615.087891
11:36:48 AM	5.230000019	63.29999924	24	77.59999847	2646.642334
11:37:48 AM	5.230000019	63.20000076	24	77.59999847	2678.196777
11:38:48 AM	5.190000057	63.20000076	24	77.69999695	2709.509766
11:39:48 AM	5.190000057	63.20000076	24	77.69999695	2740.822754
11:40:48 AM	5.190000057	63.09999847	24	77.69999695	2772.135742
11:41:49 AM	5.190000057	63.09999847	24	77.69999695	2803.44873
11:42:49 AM	5.150000095	63.09999847	24	77.80000305	2834.520508
11:43:49 AM	5.150000095	63	24	77.80000305	2865.592285
11:44:49 AM	5.150000095	63	24	77.90000153	2896.664063
11:45:48 AM	5.150000095	62.90000153	24	77.90000153	2927.73584
11:46:48 AM	5.099999905	62.90000153	24	77.90000153	2958.807617
11:47:48 AM	5.099999905	62.79999924	24	77.90000153	2989.577637
11:48:48 AM	5.099999905	62.79999924	24	78	3020.347656
11:49:49 AM	5.099999905	62.70000076	24	78	3051.117676
11:50:49 AM	5.099999905	62.79999924	24	78	3081.887695
11:51:49 AM	5.099999905	62.70000076	24	78.09999847	3112.657715
11:52:49 AM	5.099999905	62.70000076	24	78.09999847	3143.427734
11:53:48 AM	5.099999905	62.59999847	24	78.19999695	3174.197754
11:54:48 AM	5.059999943	62.59999847	24	78.19999695	3204.726318
11:55:48 AM	5.059999943	62.5	24.10000038	78.19999695	3235.254883
11:56:49 AM	5.059999943	62.5	24	78.30000305	3265.783447
11:57:49 AM	5.059999943	62.40000153	24	78.30000305	3296.312012
11:58:49 AM	5.019999981	62.40000153	24.10000038	78.30000305	3326.840576

11:59:49 AM	5.019999981	62.29999924	24	78.40000153	3357.12793
12:00:48 PM	5.019999981	62.29999924	24.10000038	78.40000153	3387.415283
12:01:48 PM	5.019999981	62.29999924	24	78.40000153	3417.702637
12:02:49 PM	5.019999981	62.29999924	24	78.5	3447.98999
12:03:49 PM	5.019999981	62.20000076	24	78.5	3478.277344
12:04:49 PM	5.349999905	62.20000076	24	78.90000153	3510.072998
12:05:49 PM	5.309999943	62.20000076	24	78.90000153	3542.110107
12:06:49 PM	5.269999981	62.20000076	23.89999962	78.90000153	3573.905762
12:07:49 PM	5.269999981	62.09999847	24	79	3605.701416
12:08:49 PM	5.230000019	62.09999847	24	79	3637.255859
12:09:48 PM	5.230000019	62.09999847	23.89999962	79	3668.810303
12:10:48 PM	5.230000019	62	24	79.09999847	3700.364746
12:11:48 PM	5.190000057	62	24	79.09999847	3731.677734
12:12:48 PM	5.190000057	62	24	79.09999847	3762.990723
12:13:49 PM	5.190000057	62	24	79.09999847	3794.303711
12:14:48 PM	5.190000057	62	24	79.19999695	3825.616699
12:15:49 PM	5.190000057	61.90000153	24	79.19999695	3856.929688
12:16:49 PM	5.150000095	61.90000153	24	79.19999695	3888.001465
12:17:49 PM	5.150000095	61.79999924	24	79.30000305	3919.073242
12:18:48 PM	5.150000095	61.79999924	24	79.30000305	3950.14502
12:19:48 PM	5.150000095	61.70000076	24	79.30000305	3981.216797
12:20:48 PM	5.150000095	61.70000076	24	79.40000153	4012.288574
12:21:48 PM	5.099999905	61.59999847	24	79.40000153	4043.360352
12:22:49 PM	5.099999905	61.59999847	24	79.40000153	4074.130371
12:23:49 PM	5.099999905	61.59999847	24	79.40000153	4104.900391
12:24:48 PM	5.099999905	61.59999847	23.89999962	79.5	4135.67041
12:25:48 PM	5.099999905	61.5	23.89999962	79.5	4166.44043
12:26:49 PM	5.099999905	61.5	23.89999962	79.59999847	4197.210449
12:27:49 PM	5.099999905	61.40000153	23.89999962	79.59999847	4227.980469
12:28:49 PM	5.099999905	61.40000153	23.89999962	79.59999847	4258.750488
12:29:48 PM	5.059999943	61.40000153	23.89999962	79.59999847	4289.279297
12:30:48 PM	5.059999943	61.40000153	23.89999962	79.69999695	4319.808105
12:31:48 PM	5.059999943	61.29999924	23.89999962	79.69999695	4350.336914
12:32:48 PM	5.059999943	61.29999924	24	79.69999695	4380.865723

12:33:49 PM	5.059999943	61.20000076	24	79.80000305	4411.394531
12:34:49 PM	5.019999981	61.09999847	23.89999962	79.80000305	4441.681641
12:35:49 PM	5.019999981	61.09999847	23.89999962	79.80000305	4471.96875
12:36:49 PM	5.019999981	61	23.89999962	79.90000153	4502.255859
12:37:49 PM	5.019999981	61	23.89999962	79.90000153	4532.542969
12:38:48 PM	5.019999981	61.09999847	23.89999962	79.90000153	4562.830078
12:39:48 PM	5.019999981	61	23.89999962	80	4593.117188
12:40:48 PM	5.389999866	60.90000153	23.89999962	80.30000305	4624.913086
12:41:49 PM	5.309999943	60.90000153	23.89999962	80.40000153	4656.950195
12:42:49 PM	5.269999981	60.90000153	23.89999962	80.40000153	4688.746094
12:43:48 PM	5.230000019	60.90000153	23.89999962	80.40000153	4720.300293
12:44:49 PM	5.230000019	60.90000153	23.89999962	80.40000153	4751.854492
12:45:49 PM	5.230000019	60.79999924	23.89999962	80.5	4783.408691
12:46:49 PM	5.230000019	60.79999924	23.89999962	80.5	4814.962891
12:47:48 PM	5.190000057	60.79999924	23.89999962	80.5	4846.275879
12:48:49 PM	5.190000057	60.70000076	23.89999962	80.5	4877.588867
12:49:49 PM	5.190000057	60.70000076	23.89999962	80.59999847	4908.901855
12:50:48 PM	5.190000057	60.70000076	23.89999962	80.59999847	4940.214844
12:51:48 PM	5.190000057	60.59999847	23.89999962	80.59999847	4971.527832
12:52:49 PM	5.150000095	60.59999847	23.89999962	80.69999695	5002.599609
12:53:49 PM	5.150000095	60.5	23.89999962	80.69999695	5033.671387
12:54:48 PM	5.150000095	60.5	23.89999962	80.69999695	5064.743164
12:55:49 PM	5.150000095	60.5	23.89999962	80.80000305	5095.814941
12:56:49 PM	5.150000095	60.5	23.89999962	80.80000305	5126.886719
12:57:48 PM	5.099999905	60.5	23.89999962	80.80000305	5157.656738
12:58:48 PM	5.099999905	60.5	23.89999962	80.90000153	5188.426758
12:59:48 PM	5.099999905	60.40000153	23.89999962	80.90000153	5219.196777
1:00:49 PM	5.099999905	60.40000153	23.89999962	80.90000153	5249.966797
1:01:49 PM	5.099999905	60.40000153	24	81	5280.736816
1:02:49 PM	5.099999905	60.40000153	23.89999962	81	5311.506836
1:03:48 PM	5.099999905	60.29999924	23.89999962	81	5342.276855
1:04:48 PM	5.099999905	60.20000076	23.89999962	81.09999847	5373.046875
1:05:48 PM	5.099999905	60.20000076	23.89999962	81.09999847	5403.816895
1:06:48 PM	5.059999943	60.20000076	23.89999962	81.09999847	5434.345703

1:07:49 PM	5.059999943	60.20000076	23.89999962	81.09999847	5464.874512
1:08:49 PM	5.059999943	60.20000076	23.89999962	81.19999695	5495.40332
1:09:48 PM	5.059999943	60.20000076	23.89999962	81.19999695	5525.932129
1:10:48 PM	5.059999943	60.20000076	23.89999962	81.19999695	5556.460938
1:11:49 PM	5.019999981	60.09999847	23.89999962	81.19999695	5586.748047
1:12:48 PM	5.019999981	60.09999847	23.89999962	81.30000305	5617.035156
1:13:49 PM	5.019999981	60.09999847	24	81.30000305	5647.322266
1:14:49 PM	5.019999981	60.09999847	23.89999962	81.30000305	5677.609375
1:15:49 PM	5.019999981	60	23.89999962	81.40000153	5707.896484
1:16:49 PM	5.019999981	60	23.89999962	81.40000153	5738.183594
1:17:48 PM	5.019999981	60	23.89999962	81.40000153	5768.470703
1:18:48 PM	5.269999981	60	23.89999962	81.69999695	5800.266602
1:19:48 PM	5.230000019	60	23.89999962	81.80000305	5831.820801
1:20:49 PM	5.230000019	60	23.89999962	81.80000305	5863.375
1:21:49 PM	5.190000057	59.90000153	23.89999962	81.80000305	5894.687988
1:22:49 PM	5.190000057	60	23.89999962	81.80000305	5926.000977
1:23:49 PM	5.190000057	59.90000153	23.89999962	81.90000153	5957.313965
1:24:49 PM	5.190000057	59.90000153	23.89999962	81.90000153	5988.626953
1:25:49 PM	5.150000095	59.90000153	24	81.90000153	6019.69873
1:26:49 PM	5.150000095	59.90000153	23.89999962	82	6050.770508
1:27:48 PM	5.150000095	59.90000153	24	82	6081.842285
1:28:49 PM	5.150000095	59.79999924	24	82	6112.914063
1:29:49 PM	5.150000095	59.79999924	24	82	6143.98584
1:30:49 PM	5.099999905	59.79999924	23.89999962	82.09999847	6174.755859
1:31:49 PM	5.099999905	59.79999924	23.89999962	82.09999847	6205.525879
1:32:48 PM	5.099999905	59.70000076	23.89999962	82.09999847	6236.295898
1:33:48 PM	5.099999905	59.70000076	23.89999962	82.09999847	6267.065918
1:34:48 PM	5.099999905	59.79999924	23.89999962	82.19999695	6297.835938
1:35:48 PM	5.099999905	59.70000076	24	82.19999695	6328.605957
1:36:48 PM	5.099999905	59.70000076	24	82.19999695	6359.375977
1:37:48 PM	5.099999905	59.70000076	24	82.30000305	6390.145996
1:38:49 PM	5.059999943	59.70000076	24	82.30000305	6420.674805
1:39:48 PM	5.059999943	59.70000076	24	82.30000305	6451.203613
1:40:48 PM	5.059999943	59.70000076	24	82.30000305	6481.732422

1:41:48 PM	5.059999943	59.59999847	23.89999962	82.40000153	6512.26123
1:42:48 PM	5.059999943	59.59999847	24	82.40000153	6542.790039
1:43:48 PM	5.059999943	59.59999847	24	82.5	6573.318848
1:44:48 PM	5.019999981	59.59999847	24	82.5	6603.605957
1:45:48 PM	5.019999981	59.59999847	24	82.5	6633.893066
1:46:48 PM	5.019999981	59.5	24	82.5	6664.180176
1:47:49 PM	5.019999981	59.5	24	82.59999847	6694.467285
1:48:49 PM	5.019999981	59.5	24	82.59999847	6724.754395
1:49:49 PM	5.019999981	59.5	24	82.59999847	6755.041504
1:50:48 PM	5.019999981	59.5	24	82.69999695	6785.328613
1:51:48 PM	5.269999981	59.5	24	82.90000153	6817.124512
1:52:48 PM	5.230000019	59.5	24	83	6848.678711
1:53:48 PM	5.190000057	59.40000153	24	83	6880.23291
1:54:49 PM	5.190000057	59.40000153	24	83	6911.545898
1:55:49 PM	5.190000057	59.40000153	24	83.09999847	6942.858887
1:56:48 PM	5.190000057	59.40000153	24	83.09999847	6974.171875
1:57:48 PM	5.150000095	59.40000153	24	83.09999847	7005.243652
1:58:49 PM	5.150000095	59.40000153	24	83.09999847	7036.31543
1:59:49 PM	5.150000095	59.40000153	24	83.09999847	7067.387207
2:00:49 PM	5.150000095	59.40000153	24	83.19999695	7098.458984
2:01:49 PM	5.150000095	59.29999924	24	83.19999695	7129.530762
2:02:49 PM	5.099999905	59.29999924	24	83.30000305	7160.300781
2:03:49 PM	5.099999905	59.29999924	24	83.30000305	7191.070801
2:04:49 PM	5.099999905	59.20000076	24	83.30000305	7221.84082
2:05:48 PM	5.099999905	59.20000076	24	83.30000305	7252.61084
2:06:49 PM	5.099999905	59.20000076	24.10000038	83.40000153	7283.380859
2:07:49 PM	5.099999905	59.20000076	24	83.40000153	7314.150879
2:08:49 PM	5.099999905	59.20000076	24.10000038	83.40000153	7344.920898
2:09:48 PM	5.099999905	59.20000076	24	83.40000153	7375.690918
2:10:48 PM	5.099999905	59.20000076	24.10000038	83.5	7406.460938
2:11:48 PM	5.059999943	59.20000076	24.10000038	83.5	7436.989746
2:12:49 PM	5.059999943	59.09999847	24	83.5	7467.518555
2:13:48 PM	5.059999943	59.09999847	24.10000038	83.5	7498.047363
2:14:48 PM	5.059999943	59.09999847	24	83.59999847	7528.576172

2:15:48 PM	5.059999943	59.09999847	24	83.59999847	7559.10498
2:16:48 PM	5.059999943	59.09999847	24	83.69999695	7589.39209
2:17:49 PM	5.019999981	59	24	83.69999695	7619.679199
2:18:49 PM	5.019999981	59	24.10000038	83.69999695	7649.966309
2:19:49 PM	5.019999981	59.09999847	24	83.69999695	7680.253418
2:20:48 PM	5.019999981	59.09999847	24	83.69999695	7710.540527
2:21:48 PM	5.019999981	59	24	83.80000305	7740.827637
2:22:48 PM	5.019999981	59	24	83.80000305	7771.114746
2:23:48 PM	5.019999981	59	24	83.80000305	7801.401855
2:24:49 PM	5.269999981	59	24	84.09999847	7833.197754
2:25:48 PM	5.230000019	59	24.10000038	84.09999847	7864.751953
2:26:48 PM	5.190000057	58.90000153	24.10000038	84.19999695	7896.064941
2:27:48 PM	5.190000057	58.90000153	24.10000038	84.19999695	7927.37793
2:28:48 PM	5.190000057	58.90000153	24.10000038	84.19999695	7958.690918
2:29:49 PM	5.190000057	58.90000153	24.10000038	84.19999695	7990.003906
2:30:49 PM	5.230000019	58.90000153	24.10000038	85.59999847	8021.075684
2:31:48 PM	3.089999914	58.90000153	24.10000038	47.5	8040.442871
2:32:48 PM	1.889999986	58.90000153	24.10000038	27.10000038	8052.328613
2:33:48 PM	1.059999943	58.79999924	24.10000038	69.40000153	8058.542969
2:34:48 PM	0.920000017	58.90000153	24.10000038	38.70000076	8064.274414
2:35:48 PM	0.550000012	58.90000153	24.10000038	56.09999847	8068.015137
2:36:48 PM	0.469999999	58.79999924	24.10000038	55.70000076	8070.850586
2:37:48 PM	0.400000006	58.90000153	24.10000038	31.29999924	8073.26416
2:38:49 PM	0.219999999	58.90000153	24.10000038	84.40000153	8074.772461
2:39:49 PM	0.25	58.79999924	24.10000038	46.70000076	8076.280762
2:40:48 PM	0.25	58.79999924	24.10000038	58.20000076	8077.789063
2:41:48 PM	0.140000001	58.70000076	24.10000038	55.70000076	8078.633789
2:42:48 PM	0.140000001	58.70000076	24.10000038	31.20000076	8079.478516
2:43:49 PM	0.109999999	58.70000076	24.10000038	65.90000153	8080.14209
2:44:48 PM	0.109999999	58.70000076	24.10000038	36.79999924	8080.805664
2:45:48 PM	0.07	58.70000076	24.10000038	79	8081.228027
2:46:49 PM	0.07	58.70000076	24.10000038	43.29999924	8081.650391
2:47:48 PM	0.07	58.70000076	24.10000038	78.40000153	8082.072754
2:48:48 PM	0.07	58.70000076	24.10000038	51.90000153	8082.495117

2:49:49 PM	0.07	58.70000076	24.10000038	30	8082.91748
2:50:48 PM	0.039999999	58.70000076	24.10000038	61.5	8083.158691
2:51:48 PM	0.039999999	58.59999847	24.20000076	34.40000153	8083.399902
2:52:48 PM	0.039999999	58.59999847	24.10000038	73.5	8083.641113
2:53:48 PM	0.039999999	58.59999847	24.10000038	40.90000153	8083.882324
2:54:48 PM	0.039999999	58.5	24.10000038	88.80000305	8084.123535
2:55:49 PM	0.039999999	58.59999847	24.10000038	48.5	8084.364746
2:56:48 PM	0.039999999	58.59999847	24.10000038	46.90000153	8084.605957

Date	CONC._A	CONC._B	TEMP_A	TEMP_B	RH_A	RH_B	VAC_A	VAC_B	DIFF_ALRM	DIFF_ALRM	RUNNUMBER_DOUBL
5/25/2022 8:36	0.036179	0.072396	22.10012	22.05128	30.7	31.1	67.7	68.2	0	0	237
5/25/2022 8:37	0.036179	0.072396	22.14896	22.17338	29.5	30.3	67.1	67.6	0	0	237
5/25/2022 8:38	0.036179	0.072396	22.17338	22.1978	31.7	32.2	67.2	67.7	0	0	237
5/25/2022 8:39	0.036179	0.072396	22.1978	22.22222	32.7	32.9	67.3	67.7	0	0	237
5/25/2022 8:40	0.036179	0.072396	22.29548	22.24664	33.4	33.4	67.4	67.9	0	0	237
5/25/2022 8:41	0.036179	0.072396	22.34432	22.3199	46.9	46.4	67.7	68.2	0	0	237
5/25/2022 8:42	0.036179	0.108651	22.34432	22.34432	50.5	51.2	67.8	68.3	0	0	237
5/25/2022 8:43	0.036179	0.108651	22.34432	22.44201	52.7	53.7	67.9	68.4	0	0	237
5/25/2022 8:44	0.036179	0.108651	22.36874	22.46642	54.7	55.7	68	68.5	0	0	237
5/25/2022 8:45	0.036179	0.108651	22.41758	22.49084	55.5	56.6	68.1	68.6	0	0	237
5/25/2022 8:46	0.036179	0.108651	22.51526	22.49084	56.2	57.3	68.2	68.7	0	0	237
5/25/2022 8:47	0.036179	0.108651	22.61295	22.53968	57.7	58.8	68.3	68.8	0	0	237
5/25/2022 8:48	0.036179	0.108651	22.66179	22.5641	58.9	59.9	68.4	68.9	0	0	237
5/25/2022 8:49	0.036179	0.108651	22.66179	22.6862	59.8	60.9	68.5	69	0	0	237
5/25/2022 8:50	0.036179	0.108651	22.66179	22.71062	59.4	60.3	68.5	69	0	0	237
5/25/2022 8:51	0.036179	0.108651	22.66179	22.78388	60.5	61.5	68.6	69.1	0	0	237
5/25/2022 8:52	0.036179	0.108651	22.78388	22.8083	61.2	62.1	68.7	69.2	0	0	237
5/25/2022 8:53	0.036179	0.108651	22.85714	22.8083	62	62.6	68.8	69.3	0	0	237
5/25/2022 8:54	0.036179	0.108651	22.90598	22.85714	63	63.5	68.9	69.4	0	0	237
5/25/2022 8:55	0.036179	0.108651	22.9304	22.9304	64.3	64.7	69	69.4	0	0	237
5/25/2022 8:56	0.036179	0.108651	22.95482	22.95482	64.6	65	69	69.5	0	0	237
5/25/2022 8:57	0.036179	0.108651	22.95482	23.02808	65	65.4	69.1	69.6	0	0	237
5/25/2022 8:58	0.036179	0.108651	23.05251	23.05251	65.7	66	69.2	69.7	0	0	237
5/25/2022 8:59	0.036179	0.108651	23.07692	23.10135	66.5	66.8	69.3	69.8	0	0	237
5/25/2022 9:00	0.036179	0.108651	23.15018	23.15018	67.3	67.7	69.3	69.8	0	0	237
5/25/2022 9:01	0.036179	0.108651	23.22345	23.19902	67.6	67.9	69.4	69.9	0	0	237
5/25/2022 9:02	0.036179	0.108651	23.22345	23.22345	67.8	68.1	69.5	70	0	0	237
5/25/2022 9:03	0.036179	0.108651	23.22345	23.27229	68.3	68.7	69.6	70.1	0	0	237
5/25/2022 9:04	0.036179	0.108651	23.27229	23.34554	68.8	69.3	69.6	70.1	0	0	237
5/25/2022 9:05	0.036179	0.108651	23.2967	23.34554	69.4	69.9	69.7	70.2	0	0	237
5/25/2022 9:06	0.036179	0.108651	23.36996	23.34554	69.9	70.3	69.8	70.3	0	0	237
5/25/2022 9:07	0.036179	0.108651	23.4188	23.36996	70.1	70.5	69.8	70.3	0	73	237
5/25/2022 9:08	0.036179	0.108651	23.44323	23.4188	66.5	66.6	69.8	70.3	0	0	237

5/25/2022 9:09	0.036179	0.108651	23.49207	23.51648	67.3	67.8	69.9	70.4	0	0	237
5/25/2022 9:10	0.036179	0.108651	23.5409	23.5409	69.4	69.8	70	70.5	0	0	237
5/25/2022 9:11	0.036179	0.108651	23.51648	23.56532	70.4	70.8	70.1	70.6	0	11	237
5/25/2022 9:12	0.036179	0.108651	23.51648	23.56532	68.5	68.7	70	70.5	0	606	237
5/25/2022 9:13	0.036179	0.108651	23.56532	23.61417	65.3	65.5	70	70.5	0	0	237
5/25/2022 9:14	0.036179	0.108651	23.68742	23.66301	65.4	65.8	70.1	70.7	0	0	237
5/25/2022 9:15	0.036179	0.108651	23.68742	23.71185	67.3	67.7	70.2	70.8	0	0	237
5/25/2022 9:16	0.036179	0.108651	23.76068	23.73626	68.6	68.9	70.3	70.8	0	0	237
5/25/2022 9:17	0.036179	0.108651	23.76068	23.73626	69.6	70	70.4	70.9	0	0	237
5/25/2022 9:18	0.036179	0.108651	23.76068	23.76068	70.4	70.7	70.4	70.9	0	194	237
5/25/2022 9:19	0.036179	0.108651	23.78511	23.76068	67.4	67.5	70.4	70.9	0	0	237
5/25/2022 9:20	0.036179	0.108651	23.76068	23.80952	65.3	65.5	70.5	71	0	0	237
5/25/2022 9:21	0.036179	0.108651	23.78511	23.88279	67.7	68.1	70.6	71.1	0	0	237
5/25/2022 9:22	0.036179	0.108651	23.83395	23.9072	69.9	70.2	70.7	71.2	0	0	237
5/25/2022 9:23	0.036179	0.108651	23.80952	23.88279	70.3	70.5	70.7	71.2	0	385	237
5/25/2022 9:24	0.036179	0.108651	23.85836	23.9072	67.2	67.3	70.7	71.2	0	0	237
5/25/2022 9:25	0.036179	0.108651	23.93163	23.9072	65.5	65.7	70.8	71.3	0	0	237
5/25/2022 9:26	0.072396	0.108651	24.00488	23.98046	68.8	69.2	70.9	71.4	0	0	237
5/25/2022 9:27	0.036179	0.108651	24.00488	23.93163	70.9	71.4	70.9	71.4	0	137	237
5/25/2022 9:28	0.036179	0.108651	24.00488	23.98046	68.5	68.7	70.9	71.4	0	736	237
5/25/2022 9:29	0.036179	0.108651	24.00488	23.98046	66.5	66.6	70.9	71.4	0	0	237
5/25/2022 9:30	0.036179	0.108651	24.05373	24.07815	66.5	66.9	71.1	71.6	0	0	237
5/25/2022 9:31	0.036179	0.108651	24.05373	24.07815	70.2	70.5	71.2	71.7	0	0	237
5/25/2022 9:32	0.036179	0.108651	24.05373	24.07815	70.1	70.3	71.1	71.7	0	469	237
5/25/2022 9:33	0.036179	0.108651	24.07815	24.12698	67.3	67.5	71.1	71.7	0	0	237
5/25/2022 9:34	0.036179	0.108651	24.07815	24.15141	65.9	66	71.2	71.8	0	0	237
5/25/2022 9:35	0.036179	0.108651	24.17583	24.20025	69.1	69.4	71.3	71.9	0	0	237
5/25/2022 9:36	0.036179	0.108651	24.12698	24.20025	70.9	71.2	71.4	71.9	0	204	237
5/25/2022 9:37	0.036179	0.072396	24.15141	24.20025	68.5	68.5	71.4	71.9	0	804	237
5/25/2022 9:38	0.036179	0.108651	24.17583	24.24908	66.7	66.8	71.4	71.9	0	0	237
5/25/2022 9:39	0.036179	0.072396	24.29793	24.29793	67.6	67.9	71.5	72.1	0	0	237
5/25/2022 9:40	0.036179	0.108651	24.29793	24.32234	71.2	71.5	71.6	72.1	0	101	237
5/25/2022 9:41	0.036179	0.072396	24.27351	24.27351	69.6	69.7	71.6	72.1	0	702	237
5/25/2022 9:42	0.036179	0.072396	24.27351	24.29793	67.6	67.7	71.6	72.1	0	0	237

5/25/2022 9:43	0.036179	0.072396	24.32234	24.32234	66.3	66.4	71.7	72.2	0	0	237
5/25/2022 9:44	0.036179	0.108651	24.32234	24.37119	69.1	69.4	71.8	72.3	0	0	237
5/25/2022 9:45	0.036179	0.072396	24.32234	24.34676	71.3	71.5	71.8	72.3	0	214	237
5/25/2022 9:46	0.036179	0.108651	24.32234	24.37119	69.1	69.2	71.8	72.3	0	814	237
5/25/2022 9:47	0.036179	0.072396	24.32234	24.37119	67.3	67.5	71.8	72.3	0	0	237
5/25/2022 9:48	0.036179	0.072396	24.39561	24.46886	66.5	66.6	71.9	72.4	0	0	237
5/25/2022 9:49	0.036179	0.072396	24.39561	24.46886	70	70.3	72.1	72.6	0	0	237
5/25/2022 9:50	0.036179	0.072396	24.34676	24.44444	70.9	71	72	72.6	0	417	237
5/25/2022 9:51	0.036179	0.072396	24.37119	24.46886	68.4	68.5	72	72.6	0	1019	237
5/25/2022 9:52	0.036179	0.072396	24.39561	24.46886	67.2	67.2	72.1	72.6	0	0	237
5/25/2022 9:53	0.036179	0.072396	24.49329	24.54212	67.6	67.8	72.2	72.7	0	0	237
5/25/2022 9:54	0.036179	0.108651	24.49329	24.49329	71.2	71.5	72.3	72.8	0	124	237
5/25/2022 9:55	0.036179	0.072396	24.42003	24.51771	69.9	69.9	72.3	72.8	0	724	237
5/25/2022 9:56	0.036179	0.072396	24.46886	24.54212	68.1	68.1	72.3	72.8	0	1326	237
5/25/2022 9:57	0.036179	0.072396	24.46886	24.56654	67.1	67	72.3	72.8	0	0	237
5/25/2022 9:58	0.036179	0.072396	24.56654	24.61539	69.1	69.2	72.5	73	0	0	237
5/25/2022 9:59	0.036179	0.072396	24.54212	24.61539	71.7	71.8	72.5	73	0	285	237
5/25/2022 10:00	0.036179	0.072396	24.54212	24.61539	69.5	69.3	72.5	73	0	888	237
5/25/2022 10:01	0.036179	0.072396	24.56654	24.61539	68	67.9	72.5	73	0	1486	237
5/25/2022 10:02	0.036179	0.072396	24.59097	24.63981	67.2	67.1	72.5	73	0	0	237
5/25/2022 10:03	0.036179	0.072396	24.63981	24.71307	69.5	69.7	72.7	73.3	0	0	237
5/25/2022 10:04	0.036179	0.072396	24.59097	24.66422	71.7	71.7	72.7	73.2	0	355	237
5/25/2022 10:05	0.036179	0.072396	24.59097	24.68864	69.5	69.3	72.7	73.2	0	964	237
5/25/2022 10:06	0.036179	0.072396	24.59097	24.68864	68.2	68	72.7	73.3	0	1560	237
5/25/2022 10:07	0.036179	0.072396	24.61539	24.71307	67.3	67.2	72.7	73.3	0	0	237
5/25/2022 10:08	0.036179	0.072396	24.63981	24.73749	68.3	68.4	66.6	67.1	0	0	237
5/25/2022 10:09	1.027376	1.064619	24.61539	24.78632	68.5	68.5	67.9	68.4	0	0	237
5/25/2022 10:10	1.969851	2.008044	24.63981	24.85959	68.8	68.7	68.8	69.3	0	0	237
5/25/2022 10:11	2.818875	2.857904	24.63981	24.90842	68.9	68.6	69.7	70.2	0	0	237
5/25/2022 10:12	3.566924	3.646464	24.63981	24.90842	69	68.6	70.6	71.1	0	0	237
5/25/2022 10:13	4.288256	4.328691	24.63981	24.90842	68.9	68.5	71.5	72	0	0	237
5/25/2022 10:14	4.939776	5.0219	24.63981	24.90842	68.9	68.4	72.5	73	0	0	237
5/25/2022 10:15	5.227875	5.269185	24.63981	24.884	68.8	68.2	73	73.5	40	0	237
5/25/2022 10:16	4.980819	5.0219	24.63981	24.90842	68.7	68.1	73.1	73.7	0	0	237

5/25/2022 10:17	5.186604	5.269185	24.61539	24.90842	68.5	67.9	73.4	73.9	325	0	237
5/25/2022 10:18	5.063019	5.104176	24.63981	24.85959	68.4	67.7	73.4	74	935	0	237
5/25/2022 10:19	5.269185	5.310531	24.59097	24.85959	68.3	67.6	73.8	74.3	0	0	237
5/25/2022 10:20	5.227875	5.269185	24.61539	24.85959	68.2	67.4	73.8	74.3	498	0	237
5/25/2022 10:21	5.145371	5.186604	24.61539	24.81075	68.1	67.3	73.8	74.4	1095	0	237
5/25/2022 10:22	5.104176	5.145371	24.61539	24.81075	67.9	67.1	73.9	74.4	1695	0	237
5/25/2022 10:23	5.063019	5.104176	24.59097	24.78632	67.8	66.9	73.9	74.4	2292	0	237
5/25/2022 10:24	5.0219	5.063019	24.59097	24.78632	67.7	66.8	73.9	74.5	2891	0	237
5/25/2022 10:25	5.269185	5.310531	24.59097	24.71307	67.6	66.6	74.2	74.8	0	0	237
5/25/2022 10:26	5.269185	5.310531	24.59097	24.73749	67.4	66.5	74.3	74.9	478	0	237
5/25/2022 10:27	5.227875	5.269185	24.61539	24.71307	67.3	66.4	74.3	74.9	1086	0	237
5/25/2022 10:28	5.186604	5.227875	24.56654	24.68864	67.3	66.3	74.4	74.9	1684	0	237
5/25/2022 10:29	5.186604	5.227875	24.56654	24.68864	67.1	66.1	74.4	74.9	2284	0	237
5/25/2022 10:30	5.145371	5.186604	24.54212	24.68864	67	66	74.4	75	2894	0	237
5/25/2022 10:31	5.145371	5.186604	24.49329	24.61539	66.9	65.8	74.5	75	3481	0	237
5/25/2022 10:32	5.104176	5.145371	24.49329	24.61539	66.8	65.8	74.5	75	4079	0	237
5/25/2022 10:33	5.104176	5.145371	24.42003	24.59097	66.7	65.6	74.6	75.1	4681	0	237
5/25/2022 10:34	5.104176	5.145371	24.39561	24.54212	66.6	65.5	74.6	75.1	5280	0	237
5/25/2022 10:35	5.104176	5.145371	24.39561	24.51771	66.5	65.4	74.6	75.1	5887	0	237
5/25/2022 10:36	5.063019	5.104176	24.37119	24.51771	66.5	65.3	74.6	75.2	0	0	237
5/25/2022 10:37	5.063019	5.104176	24.37119	24.46886	66.3	65.3	74.7	75.2	0	0	237
5/25/2022 10:38	5.0219	5.104176	24.34676	24.46886	66.3	65.1	74.7	75.2	0	0	237
5/25/2022 10:39	5.0219	5.063019	24.32234	24.44444	66.2	65.1	74.7	75.3	0	0	237
5/25/2022 10:40	5.0219	5.063019	24.32234	24.42003	66.2	65	74.8	75.3	0	0	237
5/25/2022 10:41	5.0219	5.063019	24.32234	24.42003	66	65	74.8	75.4	0	0	237
5/25/2022 10:42	5.269185	5.310531	24.32234	24.39561	66	64.9	75.1	75.7	0	0	237
5/25/2022 10:43	5.269185	5.269185	24.29793	24.39561	65.9	64.8	75.2	75.7	0	0	237
5/25/2022 10:44	5.227875	5.269185	24.32234	24.37119	65.8	64.7	75.2	75.7	0	0	237
5/25/2022 10:45	5.186604	5.227875	24.29793	24.37119	65.8	64.7	75.2	75.8	0	0	237
5/25/2022 10:46	5.186604	5.227875	24.32234	24.32234	65.7	64.6	75.2	75.8	0	0	237
5/25/2022 10:47	5.145371	5.186604	24.29793	24.29793	65.6	64.6	75.3	75.9	0	0	237
5/25/2022 10:48	5.145371	5.186604	24.27351	24.29793	65.6	64.5	75.3	75.9	0	0	237
5/25/2022 10:49	5.145371	5.186604	24.29793	24.29793	65.5	64.5	75.3	75.9	0	0	237
5/25/2022 10:50	5.104176	5.145371	24.24908	24.24908	65.4	64.4	75.4	76	0	0	237

5/25/2022 10:51	5.104176	5.145371	24.22466	24.27351	65.3	64.3	75.4	76	0	0	237
5/25/2022 10:52	5.104176	5.145371	24.22466	24.24908	65.3	64.3	75.4	76	0	0	237
5/25/2022 10:53	5.104176	5.145371	24.20025	24.22466	65.2	64.2	75.5	76	0	0	237
5/25/2022 10:54	5.104176	5.145371	24.17583	24.22466	65.2	64.2	75.5	76.1	0	0	237
5/25/2022 10:55	5.063019	5.104176	24.17583	24.20025	65.1	64.1	75.6	76.1	0	0	237
5/25/2022 10:56	5.063019	5.104176	24.17583	24.20025	65	64.1	75.6	76.1	0	0	237
5/25/2022 10:57	5.063019	5.104176	24.15141	24.17583	65	64.1	75.6	76.2	0	0	237
5/25/2022 10:58	5.063019	5.104176	24.17583	24.15141	64.9	64.1	75.7	76.2	0	0	237
5/25/2022 10:59	5.0219	5.063019	24.12698	24.15141	64.9	64	75.7	76.3	0	0	237
5/25/2022 11:00	5.0219	5.063019	24.12698	24.15141	64.8	63.9	75.8	76.3	0	0	237
5/25/2022 11:01	5.0219	5.063019	24.12698	24.15141	64.8	63.9	75.8	76.3	0	0	237
5/25/2022 11:02	5.0219	5.063019	24.12698	24.15141	64.7	63.8	75.8	76.3	0	0	237
5/25/2022 11:03	5.0219	5.0219	24.07815	24.15141	64.6	63.8	76	76.5	0	0	237
5/25/2022 11:04	5.310531	5.351916	24.07815	24.15141	64.6	63.8	76.2	76.8	0	0	237
5/25/2022 11:05	5.269185	5.310531	24.07815	24.15141	64.5	63.7	76.2	76.8	0	0	237
5/25/2022 11:06	5.227875	5.269185	24.07815	24.15141	64.5	63.7	76.3	76.8	0	0	237
5/25/2022 11:07	5.227875	5.269185	24.07815	24.15141	64.4	63.6	76.3	76.9	0	0	237
5/25/2022 11:08	5.227875	5.227875	24.07815	24.10256	64.4	63.6	76.3	76.9	0	0	237
5/25/2022 11:09	5.186604	5.227875	24.05373	24.10256	64.3	63.6	76.4	76.9	0	0	237
5/25/2022 11:10	5.186604	5.227875	24.07815	24.10256	64.3	63.4	76.4	77	0	0	237
5/25/2022 11:11	5.186604	5.227875	24.05373	24.15141	64.2	63.4	76.4	77	0	0	237
5/25/2022 11:12	5.186604	5.186604	24.07815	24.10256	64.2	63.4	76.5	77	0	0	237
5/25/2022 11:13	5.145371	5.186604	24.07815	24.07815	64.2	63.3	76.5	77.1	0	0	237
5/25/2022 11:14	5.145371	5.186604	24.07815	24.10256	64.1	63.3	76.5	77.1	0	0	237
5/25/2022 11:15	5.145371	5.186604	24.07815	24.12698	64.1	63.3	76.6	77.1	0	0	237
5/25/2022 11:16	5.145371	5.186604	24.07815	24.10256	64	63.3	76.6	77.1	0	0	237
5/25/2022 11:17	5.104176	5.145371	24.05373	24.07815	64	63.2	76.6	77.2	0	0	237
5/25/2022 11:18	5.104176	5.145371	24.07815	24.05373	63.9	63.1	76.7	77.2	0	0	237
5/25/2022 11:19	5.104176	5.145371	24.05373	24.07815	63.9	63.1	76.7	77.3	0	0	237
5/25/2022 11:20	5.104176	5.145371	24.0293	24.05373	63.8	63.1	76.7	77.3	0	0	237
5/25/2022 11:21	5.104176	5.145371	24.05373	24.05373	63.7	63	76.8	77.3	0	0	237
5/25/2022 11:22	5.104176	5.104176	24.05373	24.0293	63.7	63	76.8	77.3	0	0	237
5/25/2022 11:23	5.104176	5.104176	24.00488	24.05373	63.7	62.9	76.9	77.4	0	0	237
5/25/2022 11:24	5.063019	5.104176	24.00488	24.00488	63.7	62.9	76.9	77.4	0	0	237

5/25/2022 11:25	5.063019	5.104176	24.0293	24.00488	63.6	62.8	76.9	77.5	0	0	237
5/25/2022 11:26	5.063019	5.104176	24.00488	23.98046	63.6	62.8	76.9	77.5	0	0	237
5/25/2022 11:27	5.063019	5.104176	24.00488	23.98046	63.5	62.8	77	77.5	0	0	237
5/25/2022 11:28	5.0219	5.063019	23.98046	23.95604	63.6	62.8	77	77.6	0	0	237
5/25/2022 11:29	5.0219	5.063019	23.93163	23.98046	63.5	62.8	77.1	77.6	0	0	237
5/25/2022 11:30	5.0219	5.063019	23.95604	23.93163	63.4	62.7	77.1	77.6	0	0	237
5/25/2022 11:31	5.0219	5.063019	23.93163	23.9072	63.4	62.7	77.1	77.7	0	0	237
5/25/2022 11:32	5.0219	5.0219	23.93163	23.93163	63.4	62.6	77.2	77.7	0	0	237
5/25/2022 11:33	5.351916	5.393339	23.9072	23.9072	63.4	62.6	77.5	78	0	0	237
5/25/2022 11:34	5.269185	5.310531	23.93163	23.9072	63.3	62.5	77.5	78.1	0	0	237
5/25/2022 11:35	5.227875	5.269185	23.95604	23.93163	63.3	62.6	77.6	78.1	0	0	237
5/25/2022 11:36	5.227875	5.269185	23.93163	23.93163	63.3	62.5	77.6	78.2	0	0	237
5/25/2022 11:37	5.227875	5.227875	23.95604	23.93163	63.2	62.4	77.6	78.2	0	0	237
5/25/2022 11:38	5.186604	5.227875	23.98046	23.9072	63.2	62.4	77.7	78.2	0	0	237
5/25/2022 11:39	5.186604	5.227875	23.95604	23.9072	63.2	62.4	77.7	78.3	0	0	237
5/25/2022 11:40	5.186604	5.227875	24.00488	23.93163	63.1	62.3	77.7	78.3	0	0	237
5/25/2022 11:41	5.186604	5.186604	23.98046	23.95604	63.1	62.3	77.7	78.3	0	0	237
5/25/2022 11:42	5.145371	5.186604	23.98046	23.9072	63.1	62.3	77.8	78.4	0	0	237
5/25/2022 11:43	5.145371	5.186604	23.98046	23.95604	63	62.2	77.8	78.4	0	0	237
5/25/2022 11:44	5.145371	5.186604	24.00488	23.93163	63	61.9	77.8	78.4	0	0	237
5/25/2022 11:45	5.145371	5.186604	24.00488	23.93163	62.9	61.9	77.9	78.5	0	0	237
5/25/2022 11:46	5.145371	5.186604	24.00488	23.98046	62.9	61.9	77.9	78.5	0	0	237
5/25/2022 11:47	5.104176	5.145371	24.00488	23.95604	62.8	61.9	78	78.5	0	0	237
5/25/2022 11:48	5.104176	5.145371	24.00488	23.98046	62.8	61.8	78	78.6	0	0	237
5/25/2022 11:49	5.104176	5.145371	24.00488	23.98046	62.7	61.8	78	78.6	0	0	237
5/25/2022 11:50	5.104176	5.145371	24.00488	23.95604	62.7	61.8	78.1	78.6	0	0	237
5/25/2022 11:51	5.104176	5.145371	24.0293	23.98046	62.7	61.7	78.1	78.6	0	0	237
5/25/2022 11:52	5.104176	5.104176	24.0293	23.98046	62.7	61.8	78.1	78.7	0	0	237
5/25/2022 11:53	5.104176	5.104176	24.0293	24.00488	62.6	61.7	78.2	78.7	0	0	237
5/25/2022 11:54	5.063019	5.104176	24.0293	23.98046	62.6	61.7	78.2	78.8	0	0	237
5/25/2022 11:55	5.063019	5.104176	24.0293	23.98046	62.5	61.7	78.2	78.8	0	0	237
5/25/2022 11:56	5.063019	5.104176	24.0293	24.00488	62.5	61.6	78.3	78.8	0	0	237
5/25/2022 11:57	5.063019	5.104176	24.00488	24.00488	62.5	61.7	78.3	78.8	0	0	237
5/25/2022 11:58	5.063019	5.063019	24.0293	24.00488	62.4	61.6	78.3	78.9	0	0	237

5/25/2022 11:59	5.0219	5.063019	24.0293	23.98046	62.3	61.5	78.4	78.9	0	0	237
5/25/2022 12:00	5.0219	5.063019	24.05373	24.00488	62.3	61.5	78.4	79	0	0	237
5/25/2022 12:01	5.0219	5.063019	24.00488	23.98046	62.3	61.6	78.4	79	0	0	237
5/25/2022 12:02	5.0219	5.063019	24.00488	23.98046	62.2	61.5	78.5	79	0	0	237
5/25/2022 12:03	5.0219	5.0219	24.00488	23.98046	62.2	61.5	78.5	79	0	0	237
5/25/2022 12:04	5.351916	5.393339	24.00488	23.95604	62.2	61.5	78.9	79.4	0	0	237
5/25/2022 12:05	5.310531	5.351916	23.98046	23.95604	62.2	61.5	78.9	79.4	0	0	237
5/25/2022 12:06	5.269185	5.310531	23.93163	23.98046	62.2	61.4	78.9	79.5	0	0	237
5/25/2022 12:07	5.269185	5.269185	23.98046	23.95604	62.1	61.4	79	79.5	0	0	237
5/25/2022 12:08	5.227875	5.269185	23.95604	23.95604	62.1	61.4	79	79.5	0	0	237
5/25/2022 12:09	5.227875	5.269185	23.93163	23.95604	62.1	61.4	79	79.6	0	0	237
5/25/2022 12:10	5.227875	5.227875	23.95604	23.98046	62	61.4	79.1	79.6	0	0	237
5/25/2022 12:11	5.186604	5.227875	23.98046	23.95604	62	61.3	79.1	79.6	0	0	237
5/25/2022 12:12	5.186604	5.227875	23.95604	23.95604	62	61.4	79.1	79.7	0	0	237
5/25/2022 12:13	5.186604	5.227875	24.00488	23.98046	62	61.3	79.1	79.7	0	0	237
5/25/2022 12:14	5.186604	5.227875	23.98046	23.95604	62	61.3	79.2	79.7	0	0	237
5/25/2022 12:15	5.186604	5.186604	24.0293	23.98046	61.9	61.3	79.2	79.8	0	0	237
5/25/2022 12:16	5.145371	5.186604	24.00488	23.98046	61.9	61.3	79.2	79.8	0	0	237
5/25/2022 12:17	5.145371	5.186604	24.00488	23.93163	61.8	61.3	79.3	79.8	0	0	237
5/25/2022 12:18	5.145371	5.186604	24.0293	23.98046	61.8	61.2	79.3	79.9	0	0	237
5/25/2022 12:19	5.145371	5.186604	24.00488	23.98046	61.7	61.2	79.3	79.9	0	0	237
5/25/2022 12:20	5.145371	5.186604	24.00488	23.98046	61.7	61.2	79.4	79.9	0	0	237
5/25/2022 12:21	5.104176	5.145371	24.00488	23.98046	61.6	61.2	79.4	79.9	0	0	237
5/25/2022 12:22	5.104176	5.145371	23.95604	23.93163	61.6	61.2	79.4	80	0	0	237
5/25/2022 12:23	5.104176	5.145371	23.95604	23.95604	61.6	61.2	79.5	80	0	0	237
5/25/2022 12:24	5.104176	5.145371	23.93163	23.93163	61.5	61.1	79.5	80	0	0	237
5/25/2022 12:25	5.104176	5.145371	23.93163	23.93163	61.5	61.1	79.5	80.1	0	0	237
5/25/2022 12:26	5.104176	5.145371	23.9072	23.93163	61.5	61.2	79.6	80.1	0	0	237
5/25/2022 12:27	5.104176	5.104176	23.93163	23.9072	61.4	61.1	79.6	80.2	0	0	237
5/25/2022 12:28	5.104176	5.104176	23.9072	23.95604	61.4	61.1	79.6	80.2	0	0	237
5/25/2022 12:29	5.063019	5.104176	23.9072	23.9072	61.4	61.1	79.7	80.2	0	0	237
5/25/2022 12:30	5.063019	5.104176	23.93163	23.9072	61.3	61	79.7	80.2	0	0	237
5/25/2022 12:31	5.063019	5.104176	23.93163	23.93163	61.3	61.1	79.7	80.3	0	0	237
5/25/2022 12:32	5.063019	5.104176	23.93163	23.95604	61.3	61.1	79.7	80.3	0	0	237

5/25/2022 12:33	5.063019	5.104176	23.95604	23.95604	61.2	61.1	79.8	80.3	0	0	237
5/25/2022 12:34	5.0219	5.063019	23.93163	23.93163	61.1	61	79.8	80.3	0	0	237
5/25/2022 12:35	5.0219	5.063019	23.93163	23.98046	61.1	61	79.8	80.4	0	0	237
5/25/2022 12:36	5.0219	5.063019	23.93163	23.93163	61	61	79.9	80.4	0	0	237
5/25/2022 12:37	5.0219	5.063019	23.9072	23.9072	61	61	79.9	80.5	0	0	237
5/25/2022 12:38	5.0219	5.063019	23.85836	23.9072	61	61	79.9	80.5	0	0	237
5/25/2022 12:39	5.0219	5.0219	23.9072	23.9072	61	60.9	80	80.5	0	0	237
5/25/2022 12:40	5.393339	5.4348	23.88279	23.9072	60.9	60.9	80.3	80.9	0	0	237
5/25/2022 12:41	5.310531	5.351916	23.88279	23.9072	60.9	60.9	80.3	80.9	0	0	237
5/25/2022 12:42	5.269185	5.310531	23.85836	23.93163	60.9	60.9	80.4	81	0	0	237
5/25/2022 12:43	5.227875	5.269185	23.9072	23.93163	60.9	60.9	80.4	81	0	0	237
5/25/2022 12:44	5.227875	5.269185	23.9072	23.93163	60.9	60.9	80.4	81	0	0	237
5/25/2022 12:45	5.227875	5.269185	23.93163	23.9072	60.8	60.9	80.4	81	0	0	237
5/25/2022 12:46	5.227875	5.227875	23.93163	23.9072	60.8	60.8	80.5	81.1	0	0	237
5/25/2022 12:47	5.186604	5.227875	23.93163	23.93163	60.8	60.9	80.5	81.1	0	0	237
5/25/2022 12:48	5.186604	5.227875	23.93163	23.93163	60.7	60.9	80.5	81.2	0	0	237
5/25/2022 12:49	5.186604	5.227875	23.93163	23.9072	60.7	60.8	80.6	81.2	0	0	237
5/25/2022 12:50	5.186604	5.227875	23.9072	23.95604	60.7	60.8	80.6	81.2	0	0	237
5/25/2022 12:51	5.186604	5.186604	23.88279	23.9072	60.6	60.7	80.6	81.2	0	0	237
5/25/2022 12:52	5.145371	5.186604	23.85836	23.95604	60.6	60.7	80.7	81.3	0	0	237
5/25/2022 12:53	5.145371	5.186604	23.85836	23.93163	60.5	60.7	80.7	81.3	0	0	237
5/25/2022 12:54	5.145371	5.186604	23.93163	23.93163	60.5	60.7	80.7	81.3	0	0	237
5/25/2022 12:55	5.145371	5.186604	23.85836	23.9072	60.5	60.7	80.8	81.4	0	0	237
5/25/2022 12:56	5.145371	5.186604	23.88279	23.9072	60.5	60.7	80.8	81.4	0	0	237
5/25/2022 12:57	5.104176	5.186604	23.88279	23.9072	60.5	60.7	80.8	81.4	0	0	237
5/25/2022 12:58	5.145371	5.145371	23.88279	23.9072	60.5	60.7	80.9	81.4	0	0	237
5/25/2022 12:59	5.104176	5.145371	23.88279	23.95604	60.4	60.7	80.9	81.5	0	0	237
5/25/2022 13:00	5.104176	5.145371	23.95604	23.9072	60.4	60.7	80.9	81.5	0	0	237
5/25/2022 13:01	5.104176	5.145371	23.95604	23.93163	60.4	60.6	81	81.5	0	0	237
5/25/2022 13:02	5.104176	5.145371	23.93163	23.9072	60.4	60.6	81	81.6	0	0	237
5/25/2022 13:03	5.104176	5.145371	23.9072	23.95604	60.3	60.6	81	81.6	0	0	237
5/25/2022 13:04	5.104176	5.104176	23.88279	23.93163	60.2	60.6	81.1	81.6	0	0	237
5/25/2022 13:05	5.104176	5.104176	23.88279	23.93163	60.3	60.5	81.1	81.7	0	0	237
5/25/2022 13:06	5.063019	5.104176	23.88279	23.93163	60.2	60.5	81.1	81.7	0	0	237

5/25/2022 13:07	5.063019	5.104176	23.85836	23.93163	60.2	60.5	81.1	81.7	0	0	237
5/25/2022 13:08	5.063019	5.104176	23.85836	23.9072	60.2	60.5	81.2	81.7	0	0	237
5/25/2022 13:09	5.063019	5.104176	23.88279	23.9072	60.2	60.5	81.2	81.8	0	0	237
5/25/2022 13:10	5.063019	5.104176	23.9072	23.93163	60.2	60.5	81.2	81.8	0	0	237
5/25/2022 13:11	5.0219	5.063019	23.9072	23.98046	60.1	60.5	81.3	81.8	0	0	237
5/25/2022 13:12	5.0219	5.063019	23.93163	23.93163	60.1	60.4	81.3	81.8	0	0	237
5/25/2022 13:13	5.0219	5.063019	23.95604	23.93163	60.1	60.4	81.3	81.9	0	0	237
5/25/2022 13:14	5.0219	5.063019	23.93163	23.93163	60.1	60.4	81.4	81.9	0	0	237
5/25/2022 13:15	5.0219	5.063019	23.93163	23.95604	60	60.4	81.4	82	0	0	237
5/25/2022 13:16	5.0219	5.0219	23.93163	23.95604	60	60.3	81.4	82	0	0	237
5/25/2022 13:17	5.0219	5.0219	23.93163	23.93163	60	60.4	81.4	82	0	0	237
5/25/2022 13:18	5.269185	5.310531	23.88279	23.9072	60	60.4	81.7	82.3	0	0	237
5/25/2022 13:19	5.227875	5.269185	23.9072	23.95604	60	60.3	81.8	82.3	0	0	237
5/25/2022 13:20	5.227875	5.227875	23.88279	23.93163	60	60.3	81.8	82.4	0	0	237
5/25/2022 13:21	5.186604	5.227875	23.88279	23.93163	59.9	60.3	81.8	82.4	0	0	237
5/25/2022 13:22	5.186604	5.227875	23.88279	23.95604	60	60.3	81.9	82.4	0	0	237
5/25/2022 13:23	5.186604	5.227875	23.93163	23.93163	59.9	60.3	81.9	82.5	0	0	237
5/25/2022 13:24	5.186604	5.186604	23.93163	23.95604	59.9	60.3	81.9	82.5	0	0	237
5/25/2022 13:25	5.145371	5.186604	23.93163	23.98046	59.9	60.2	81.9	82.5	0	0	237
5/25/2022 13:26	5.145371	5.186604	23.95604	23.98046	59.9	60.2	82	82.5	0	0	237
5/25/2022 13:27	5.145371	5.186604	24.00488	23.98046	59.9	60.2	82	82.6	0	0	237
5/25/2022 13:28	5.145371	5.186604	23.98046	23.98046	59.8	60.2	82	82.6	0	0	237
5/25/2022 13:29	5.104176	5.186604	24.00488	23.98046	59.8	60.2	82.1	82.6	0	0	237
5/25/2022 13:30	5.104176	5.145371	23.93163	23.98046	59.8	60.1	82.1	82.7	0	0	237
5/25/2022 13:31	5.104176	5.145371	23.93163	23.98046	59.7	60.1	82.1	82.7	0	0	237
5/25/2022 13:32	5.104176	5.145371	23.93163	23.98046	59.7	60.1	82.1	82.7	0	0	237
5/25/2022 13:33	5.104176	5.145371	23.93163	23.98046	59.7	60.1	82.1	82.8	0	0	237
5/25/2022 13:34	5.104176	5.145371	23.93163	23.98046	59.7	60.1	82.2	82.8	0	0	237
5/25/2022 13:35	5.104176	5.145371	23.95604	23.98046	59.7	60.1	82.2	82.8	0	0	237
5/25/2022 13:36	5.104176	5.145371	23.98046	23.98046	59.7	60.1	82.2	82.8	0	0	237
5/25/2022 13:37	5.104176	5.104176	23.95604	23.98046	59.7	60	82.3	82.8	0	0	237
5/25/2022 13:38	5.063019	5.104176	23.98046	23.98046	59.7	60.1	82.3	82.9	0	0	237
5/25/2022 13:39	5.063019	5.104176	24.00488	24.00488	59.7	60.1	82.3	82.9	0	0	237
5/25/2022 13:40	5.063019	5.104176	24.00488	23.98046	59.6	60.1	82.3	82.9	0	0	237

5/25/2022 13:41	5.063019	5.104176	23.95604	23.98046	59.6	60	82.4	83	0	0	237
5/25/2022 13:42	5.063019	5.104176	23.95604	23.98046	59.6	60	82.4	83	0	0	237
5/25/2022 13:43	5.063019	5.104176	23.95604	24.00488	59.6	60	82.4	83	0	0	237
5/25/2022 13:44	5.0219	5.063019	23.95604	23.98046	59.6	59.9	82.5	83.1	0	0	237
5/25/2022 13:45	5.0219	5.063019	23.95604	24.00488	59.6	60	82.5	83.1	0	0	237
5/25/2022 13:46	5.0219	5.063019	23.98046	23.98046	59.5	60	82.5	83.1	0	0	237
5/25/2022 13:47	5.0219	5.063019	23.95604	23.98046	59.5	59.9	82.6	83.1	0	0	237
5/25/2022 13:48	5.0219	5.063019	23.95604	23.98046	59.6	59.9	82.6	83.2	0	0	237
5/25/2022 13:49	5.0219	5.063019	23.98046	24.00488	59.5	59.9	82.6	83.2	0	0	237
5/25/2022 13:50	5.0219	5.0219	24.00488	24.0293	59.5	59.9	82.7	83.3	0	0	237
5/25/2022 13:51	5.269185	5.310531	24.0293	24.05373	59.5	59.9	82.9	83.5	0	0	237
5/25/2022 13:52	5.227875	5.269185	24.00488	24.0293	59.5	59.9	83	83.6	0	0	237
5/25/2022 13:53	5.186604	5.227875	24.00488	24.0293	59.4	59.8	83	83.6	0	0	237
5/25/2022 13:54	5.186604	5.227875	24.00488	24.05373	59.4	59.8	83	83.6	0	0	237
5/25/2022 13:55	5.186604	5.227875	23.98046	24.00488	59.4	59.8	83.1	83.6	0	0	237
5/25/2022 13:56	5.186604	5.186604	24.00488	24.0293	59.4	59.8	83.1	83.7	0	0	237
5/25/2022 13:57	5.145371	5.186604	24.00488	24.00488	59.4	59.8	83.1	83.7	0	0	237
5/25/2022 13:58	5.145371	5.186604	24.0293	24.00488	59.4	59.9	83.1	83.8	0	0	237
5/25/2022 13:59	5.145371	5.186604	24.00488	24.00488	59.4	59.8	83.2	83.8	0	0	237
5/25/2022 14:00	5.145371	5.186604	24.0293	24.05373	59.4	59.8	83.2	83.8	0	0	237
5/25/2022 14:01	5.145371	5.186604	24.0293	24.05373	59.3	59.8	83.2	83.8	0	0	237
5/25/2022 14:02	5.104176	5.186604	24.0293	24.05373	59.3	59.8	83.3	83.9	0	0	237
5/25/2022 14:03	5.104176	5.145371	24.0293	24.10256	59.3	59.7	83.3	83.9	0	0	237
5/25/2022 14:04	5.104176	5.145371	24.0293	24.05373	59.2	59.7	83.3	83.9	0	0	237
5/25/2022 14:05	5.104176	5.145371	24.05373	24.10256	59.2	59.7	83.3	83.9	0	0	237
5/25/2022 14:06	5.104176	5.145371	24.0293	24.10256	59.2	59.7	83.4	83.9	0	0	237
5/25/2022 14:07	5.104176	5.145371	24.0293	24.10256	59.2	59.7	83.4	84	0	0	237
5/25/2022 14:08	5.104176	5.145371	24.05373	24.05373	59.2	59.7	83.4	84	0	0	237
5/25/2022 14:09	5.104176	5.145371	24.00488	24.05373	59.2	59.6	83.5	84	0	0	237
5/25/2022 14:10	5.104176	5.104176	24.0293	24.10256	59.2	59.7	83.5	84.1	0	0	237
5/25/2022 14:11	5.063019	5.104176	24.0293	24.12698	59.2	59.6	83.5	84.1	0	0	237
5/25/2022 14:12	5.063019	5.104176	24.0293	24.10256	59.2	59.7	83.5	84.1	0	0	237
5/25/2022 14:13	5.063019	5.104176	24.05373	24.12698	59.1	59.7	83.6	84.1	0	0	237
5/25/2022 14:14	5.063019	5.104176	24.0293	24.12698	59.1	59.7	83.6	84.2	0	0	237

5/25/2022 14:15	5.063019	5.104176	24.0293	24.15141	59.1	59.7	83.6	84.2	0	0	237
5/25/2022 14:16	5.063019	5.104176	24.05373	24.12698	59.1	59.6	83.6	84.2	0	0	237
5/25/2022 14:17	5.0219	5.063019	24.05373	24.15141	59	59.6	83.7	84.3	0	0	237
5/25/2022 14:18	5.0219	5.063019	24.05373	24.12698	59	59.6	83.7	84.3	0	0	237
5/25/2022 14:19	5.0219	5.063019	24.0293	24.12698	59	59.6	83.7	84.3	0	0	237
5/25/2022 14:20	5.0219	5.063019	24.05373	24.12698	59.1	59.6	83.7	84.3	0	0	237
5/25/2022 14:21	5.0219	5.063019	24.0293	24.15141	59	59.6	83.8	84.4	0	0	237
5/25/2022 14:22	5.0219	5.063019	24.0293	24.15141	59	59.5	83.8	84.4	0	0	237
5/25/2022 14:23	5.0219	5.0219	24.05373	24.15141	59	59.5	83.8	84.4	0	0	237
5/25/2022 14:24	5.269185	5.310531	24.0293	24.15141	59	59.6	84.1	84.7	0	0	237
5/25/2022 14:25	5.227875	5.269185	24.05373	24.15141	59	59.6	84.1	84.7	0	0	237
5/25/2022 14:26	5.186604	5.227875	24.07815	24.17583	58.9	59.5	84.1	84.7	0	0	237
5/25/2022 14:27	5.186604	5.227875	24.0293	24.15141	58.9	59.5	84.2	84.8	0	0	237
5/25/2022 14:28	5.186604	5.227875	24.05373	24.17583	58.9	59.5	84.2	84.8	0	0	237
5/25/2022 14:29	5.186604	5.186604	24.07815	24.17583	58.9	59.4	84.2	84.8	0	0	237
5/25/2022 14:30	5.227875	5.269185	24.05373	24.17583	58.9	59.5	85.7	86.3	0	0	237
5/25/2022 14:31	3.092876	3.132171	24.07815	24.17583	58.9	59.4	47.6	48	0	0	237
5/25/2022 14:32	1.893579	1.931696	24.07815	24.15141	58.9	59.4	27.1	27.3	0	0	237
5/25/2022 14:33	1.064619	1.1019	24.0293	24.15141	58.8	59.4	69.5	69.9	0	0	237
5/25/2022 14:34	0.915875	0.953004	24.07815	24.15141	58.9	59.4	38.7	39	0	0	237
5/25/2022 14:35	0.546675	0.583424	24.10256	24.17583	58.8	59.4	55.8	56.3	0	0	237
5/25/2022 14:36	0.473291	0.509964	24.07815	24.17583	58.9	59.5	55.8	56.2	0	0	237
5/25/2022 14:37	0.400059	0.436656	24.07815	24.20025	58.9	59.4	31.3	31.6	0	0	237
5/25/2022 14:38	0.217644	0.254051	24.10256	24.17583	58.9	59.4	84.5	85.1	0	0	237
5/25/2022 14:39	0.254051	0.290496	24.07815	24.20025	58.8	59.4	46.7	47.1	0	0	237
5/25/2022 14:40	0.254051	0.290496	24.10256	24.20025	58.7	59.4	57.9	58.4	0	0	237
5/25/2022 14:41	0.144944	0.217644	24.07815	24.17583	58.8	59.3	55.7	56.1	0	0	237
5/25/2022 14:42	0.144944	0.181275	24.07815	24.20025	58.7	59.3	31.2	31.5	0	0	237
5/25/2022 14:43	0.108651	0.144944	24.07815	24.20025	58.7	59.3	65.9	66.4	0	0	237
5/25/2022 14:44	0.072396	0.144944	24.07815	24.17583	58.7	59.4	36.8	37.2	0	0	237
5/25/2022 14:45	0.072396	0.108651	24.07815	24.15141	58.7	59.4	79	79.6	0	0	237
5/25/2022 14:46	0.072396	0.108651	24.07815	24.20025	58.7	59.3	43.3	43.7	0	0	237
5/25/2022 14:47	0.072396	0.108651	24.07815	24.20025	58.7	59.4	78.2	78.7	0	0	237
5/25/2022 14:48	0.072396	0.108651	24.07815	24.17583	58.6	59.4	51.9	52.4	0	0	237

5/25/2022 14:49	0.072396	0.108651	24.10256	24.20025	58.7	59.3	29.9	30.1	0	0	237
5/25/2022 14:50	0.036179	0.108651	24.10256	24.17583	58.7	59.3	61.6	62.1	0	0	237
5/25/2022 14:51	0.036179	0.108651	24.15141	24.22466	58.6	59.3	34.4	34.8	0	0	237
5/25/2022 14:52	0.036179	0.108651	24.15141	24.22466	58.6	59.4	73.6	74.1	0	0	237
5/25/2022 14:53	0.036179	0.108651	24.15141	24.22466	58.6	59.3	40.9	41.2	0	0	237
5/25/2022 14:54	0.036179	0.108651	24.15141	24.22466	58.5	59.3	88.8	89.5	0	0	237
5/25/2022 14:55	0.036179	0.108651	24.07815	24.22466	58.6	59.2	48.5	48.9	0	0	237
5/25/2022 14:56	0.036179	0.108651	24.07815	24.22466	58.6	59.3	46.6	47	0	0	237
