# Supplementary material for: Chlorine dioxide gas sterilization of cold-chain or temperature-sensitive devices
Source: PLoS One. 2026 Aug 27;21(8):e0321345. doi: 10.1371/journal.pone.0321345 (PMC13521399; doi:10.1371/journal.pone.0321345)
Supplement: Supplementary file 2 [file pone.0321345.s002.pdf]

BARON
ANALYTICAL LABORATORIES

June 23, 2022

[redacted]
ClorDiSys Solutions, Inc.
50 Tannery Road Suite 1
Branchburg, NJ 08876

Re: [redacted] M710 Chlorine Dioxide Sterilization
PO# [redacted]
BC# 176458-A

Equipment and Materials:
- Type 1 HPLC Grade Distilled Water
- Dionex Ion Chromatography system
- Dionex IonPac Analytical Column 2x200mm

Sample Preparation:
One hundred forty (140) syringe components were assembled and filled with Type 1 HPLC Grade Distilled Water to the fill line of 170µL. Seventy (70) filled syringes (176458-1) were used as blanks or controls and the remaining 70 (176468-3) were exposed to Chlorine Dioxide Sterilization on 5/25/22.

Additionally, 60 syringe components were assembled. Thirty (30) assembled and unfilled syringes (176458-2) were used as blanks or controls, and the remaining 30 (176458-4) were exposed to Chlorine Dioxide Sterilization on 5/25/22.

Test Method:
Samples 176458-1 and 176458-3 were split into three different composite groups, each made up of 23 syringes. The contents of the syringes were composited into three clean 4mL glass vials. The vials were then run in duplicate for the specified three analytes. Each run is tabulated in the table below with an average value reported for all three composites.

Samples 176458-2 and 176458-4 were split into three different composite groups, each group was made up of 10 syringes. Each composite of syringes was then extracted in exactly 250mL of deionized water for 2 hours at room temperature. The solution from each composite was then run in duplicate for the specified three analytes. Each run is tabulated below with an average value reported for all three of the composites.

Please contact Baron Analytical Laboratories if we can be of further assistance.

[redacted]
Laboratory Director

KO/dkh
Reviewed [redacted]

This report is submitted with the understanding that it is not to be reproduced for advertising or other purposes over our signature without our express written permission. We do not accept any liability concerning the use of these results.
Not responsible for samples left over 30 days after receipt of report.

CT Public Health Laboratory Number 0440 EPA Number CT015

181 Research Dr #1, Milford, CT 06460 | 203.874.5678

Results:

		Chlorite (ClO_2^-)			Chloride (Cl^-)			Chlorate (ClO_3^-)		
	Sample	Measurement 1 5-26-2022	Measurement 2 5-27-2022	Average (mg/L)	Measurement 1 5-26-2022	Measurement 2 5-27-2022	Average (mg/L)	Measurement 1 5-26-2022	Measurement 2 5-27-2022	Average (mg/L)
Blank 176458-1	Composite 1	ND<0.14	ND<0.14	ND<0.14	ND<0.16	ND<0.16	ND<0.16	ND<0.07	ND<0.07	ND<0.07
	Composite 2	ND<0.14	ND<0.14	ND<0.14	ND<0.16	ND<0.16	ND<0.16	ND<0.07	ND<0.07	ND<0.07
	Composite 3	ND<0.14	ND<0.14	ND<0.14	0.35	ND<0.16	0.26	0.35	ND<0.07	0.21
	Average			ND<0.14			0.19			0.12
Sterilized 176458-3	Composite 1	ND<0.14	ND<0.14	ND<0.14	ND<0.16	ND<0.16	ND<0.16	0.40	ND<0.07	0.24
	Composite 2	ND<0.14	ND<0.14	ND<0.14	ND<0.16	0.71	0.44	ND<0.07	ND<0.07	ND<0.07
	Composite 3	ND<0.14	ND<0.14	ND<0.14	ND<0.16	ND<0.16	ND<0.16	0.39	ND<0.07	0.23
	Average			ND<0.14			0.25			0.18
			mg/L	ND<0.14		mg/L	0.06		mg/L	0.06
			ug/ syringe	NDV<24		ug/ syringe	10		ug/ syringe	10

BARON
ANALYTICAL LABORATORIES

BC#176458-A

		Chlorite (ClO_2^-)			Chloride (Cl^-)			Chlorate (ClO_3^-)		
	Sample	Measurement 1 5-26-2022	Measurement 2 5-27-2022	Average (mg/L)	Measurement 1 5-26-2022	Measurement 2 5-27-2022	Average (mg/L)	Measurement 1 5-26-2022	Measurement 2 5-27-2022	Average (mg/L)
Blank 176458-2	Composite 1	ND<0.14	ND<0.14	ND<0.14	0.21	ND<0.16	0.19	ND<0.07	ND<0.07	ND<0.07
	Composite 2	ND<0.14	ND<0.14	ND<0.14	ND<0.16	ND<0.16	ND<0.16	ND<0.07	0.27	0.17
	Composite 3	ND<0.14	ND<0.14	ND<0.14	ND<0.16	ND<0.16	ND<0.16	ND<0.07	ND<0.07	ND<0.07
	Average			ND<0.14			0.17			0.12
Sterilized 176458-4	Composite 1	ND<0.14	ND<0.14	ND<0.14	ND<0.16	ND<0.16	ND<0.16	ND<0.07	ND<0.07	ND<0.07
	Composite 2	ND<0.14	ND<0.14	ND<0.14	ND<0.16	ND<0.16	ND<0.16	ND<0.07	ND<0.07	ND<0.07
	Composite 3	ND<0.14	ND<0.14	ND<0.14	ND<0.16	ND<0.16	ND<0.16	ND<0.07	ND<0.07	ND<0.07
	Average			ND<0.14			ND<0.16			ND<0.07
			mg/L	ND<0.14		mg/L	-0.01		mg/L	-0.05
			ug/ syringe	NDV<24		ug/ syringe	-2		ug/ syringe	-9
