# Supplementary material for: Chlorine dioxide gas sterilization of cold-chain or temperature-sensitive devices
Source: PLoS One. 2026 Aug 27;21(8):e0321345. doi: 10.1371/journal.pone.0321345 (PMC13521399; doi:10.1371/journal.pone.0321345)
Supplement: Supplementary file 3 [file pone.0321345.s004.pdf]

Thank you for your article and effort in the field of sterilization of temperature-sensitive products using chlorine dioxide gas. Your study in this area is very valuable and shows the areas of alternative sterilization with EtO.

However, a few points are suggested to improve and strengthen the article:

1. The Materials and Methods section would benefit from a more detailed and comprehensive description of the sterilization protocols and the residual and ingress testing procedures. This will improve reproducibility and clarity for readers.
2. Please include a dedicated section that clearly explains the statistical methods and data analysis approaches used in the study.
3. Results should be presented with more quantitative detail in the text, helping readers better understand the findings and their significance.
4. The Introduction would be stronger with a clearer statement of the study's objectives and novelty to help frame the work's contribution.
5. The Abstract should be revised to incorporate key quantitative results and major conclusions succinctly.
6. Consider revising the overall structure and organization of the manuscript to enhance logical flow and readability.
7. Language and style could be polished in places to improve clarity and reduce ambiguity.
8. Clearly discuss the limitations of the current study and suggest directions for future research.

Addressing these points will substantially enhance the quality and impact of your manuscript. I look forward to reviewing a revised version.

Best regards
