# Supplementary material for: Chlorine dioxide gas sterilization of cold-chain or temperature-sensitive devices
Source: PLoS One. 2026 Aug 27;21(8):e0321345. doi: 10.1371/journal.pone.0321345 (PMC13521399; doi:10.1371/journal.pone.0321345)
Supplement: Supplementary file 4 [file pone.0321345.s003.docx]

**Response Letter to April 7 Revision Request**

Contents

[Dear PLOS One Reviewers,	1](#_Toc198808722)

[Journal Requirements & Responses	2](#_Toc198808723)

[Reviewers’ Comments and Responses	3](#_Toc198808724)

[1. Is the manuscript technically sound, and do the data support the conclusions?	3](#_Toc198808725)

[2. Has the statistical analysis been performed appropriately and rigorously?	4](#_Toc198808726)

[3. Have the authors made all data underlying the findings in their manuscript fully available?	4](#_Toc198808727)

[4. Is the manuscript presented in an intelligible fashion and written in standard English?	4](#_Toc198808728)

[5. Review Comments to the Author	4](#_Toc198808729)

[Reviewer #1	5](#_Toc198808730)

[Reviewer #2	5](#_Toc198808731)

[6. PLOS authors have the option to publish the peer review history of their article (what does this mean?).	6](#_Toc198808732)

# Dear PLOS One Reviewers,

Thank you for the opportunity to submit a revised version of this paper. I have pulled your requirements and responses into this document so that we can easily respond.

We have responded to each using burgundy Arial 12pt font to help differentiate our responses from your requests.

Please let us know what other questions you have.

Sincerely,

Kirk Livingston, Emily Lorcheim, Paul Lorcheim

# Journal Requirements & Responses

1. Please ensure that your manuscript meets PLOS ONE's style requirements, including those for file naming. The PLOS ONE style templates can be found at <https://journals.plos.org/plosone/s/file?id=wjVg/PLOSOne_formatting_sample_main_body.pdf> and <https://journals.plos.org/plosone/s/file?id=ba62/PLOSOne_formatting_sample_title_authors_affiliations.pdf>
2. We note that the grant information you provided in the ‘Funding Information’ and ‘Financial Disclosure’ sections do not match. When you resubmit, please ensure that you provide the correct grant numbers for the awards you received for your study in the ‘Funding Information’ section.✓
   a. Add this Funding Information:
      1. This paper was funded by ClorDiSys Solutions, LLC, <https://clordisys.com/>
   b. Add this Financial Disclosure:
      i. Ms. Emily Lorcheim is an employee of ClorDiSys Solutions, LLC.
         1. She played a role in study design, data collection and analysis, the decision to publish, and the preparation of the manuscript.
         2. <https://clordisys.com/>
      ii. Mr. Paul Lorcheim is an employee of ClorDiSys Solutions, LLC.
          1. He played a role in study design, data collection and analysis, the decision to publish, and the preparation of the manuscript.
          2. <https://clordisys.com/>
      iii. Mr. Kirk Livingston is an independent science writer and an employee of Livingston Communication, Inc. ClorDiSys Solutions, LLC. funded his role in preparing this manuscript.
           1. He played a role in the preparation of the manuscript.
           2. <https://livingstoncontent.com/>
      iv. Because the primary authors are employees of ClorDiSys Solutions, LLC, the funders played a role in the study design, data collection and analysis, the decision to publish and the preparation of the manuscript.
3. Thank you for stating the following financial disclosure: “ClorDisys Solutions, Inc.” Please state what role the funders took in the study. If the funders had no role, please state: "The funders had no role in study design, data collection and analysis, decision to publish, or preparation of the manuscript." If this statement is not correct you must amend it as needed. Please include this amended Role of Funder statement in your cover letter; we will change the online submission form on your behalf.✓
4. We note that your Data Availability Statement is currently as follows: All relevant data are within the manuscript and its Supporting Information files. Please confirm at this time whether or not your submission contains all raw data required to replicate the results of your study. Authors must share the “minimal data set” for their submission. PLOS defines the minimal data set to consist of the data required to replicate all study findings reported in the article, as well as related metadata and methods (<https://journals.plos.org/plosone/s/data-availability#locminimal-data-set-definition>). For example, authors should submit the following data:

- The values behind the means, standard deviations and other measures reported;
- The values used to build graphs;
- The points extracted from images for analysis.

Authors do not need to submit their entire data set if only a portion of the data was used in the reported study. If your submission does not contain these data, please either upload them as Supporting Information files or deposit them to a stable, public repository and provide us with the relevant URLs, DOIs, or accession numbers. For a list of recommended repositories, please see <https://journals.plos.org/plosone/s/recommended-repositories>. If there are ethical or legal restrictions on sharing a de-identified data set, please explain them in detail (e.g., data contain potentially sensitive information, data are owned by a third-party organization, etc.) and who has imposed them (e.g., an ethics committee). Please also provide contact information for a data access committee, ethics committee, or other institutional body to which data requests may be sent. If data are owned by a third party, please indicate how others may request data access.

5. Please amend your authorship list in your manuscript file to include author ~~Dr.~~ (Mr.) Kirk Livingston.✓
6. Please ensure that you refer to Figure 1 in your text as, if accepted, production will need this reference to link the reader to the figure.

# Reviewers’ Comments and Responses

## 1. Is the manuscript technically sound, and do the data support the conclusions?

The manuscript must describe a technically sound piece of scientific research with data that supports the conclusions. Experiments must have been conducted rigorously, with appropriate controls, replication, and sample sizes. The conclusions must be drawn appropriately based on the data presented.

- Reviewer #1: Partly
- Reviewer #2: Yes

## 2. Has the statistical analysis been performed appropriately and rigorously?

- Reviewer #1: No
- Reviewer #2: I Don't Know

## 3. Have the authors made all data underlying the findings in their manuscript fully available?

The PLOS Data policy requires authors to make all data underlying the findings described in their manuscript fully available without restriction, with rare exception (please refer to the Data Availability Statement in the manuscript PDF file). The data should be provided as part of the manuscript or its supporting information, or deposited to a public repository. For example, in addition to summary statistics, the data points behind means, medians and variance measures should be available. If there are restrictions on publicly sharing data—e.g. participant privacy or use of data from a third party—those must be specified.

- Reviewer #1: No
- Reviewer #2: Yes

## 4. Is the manuscript presented in an intelligible fashion and written in standard English?

PLOS ONE does not copyedit accepted manuscripts, so the language in submitted articles must be clear, correct, and unambiguous. Any typographical or grammatical errors should be corrected at revision, so please note any specific errors here.

- Reviewer #1: No
- Reviewer #2: Yes

## 5. Review Comments to the Author

Please use the space provided to explain your answers to the questions above. You may also include additional comments for the author, including concerns about dual publication, research ethics, or publication ethics. (Please upload your review as an attachment if it exceeds 20,000 characters)

### Reviewer #1

The materials and methods section are poorly written with no coherent information explaining how the experiments were done. Although you stated all the materials used, the exact protocols or experiment processes are not elucidated in the write-up.

##### Author Responses

- Added new information about
  - Sterilization process
  - Ingress samples and lab procedure
  - Residual sample and lab procedure.
- Included the sterilization protocol
  - Added in sterilization protocol
- Included lab protocol
  - Lab Protocol included for residual testing
  - Lab Protocol/experiment process for ingress testing included

Also, improve the discussion in totality by specifically stating how data analysis was done, and which statistical tools were used.

##### Author Response

- Have responded with a section entitled “Data Analysis and Statistical Methods”

Lastly, your data/results are not well explained and there is no information on how experiment is scientifically relevant.

##### Author Responses

- See additional sections included, noted above.

### Reviewer #2

Prepare abstract as more informative. In the abstract, include quantitative results (e.g., specific residual levels of chlorite, chlorate, and chloride) to make the findings more impactful.✓ Briefly mention the implications of the study (e.g., potential as an alternative to EtO sterilization).✓

1. Provide a clearer statement of the study's objectives and hypotheses at the end of introduction. Include a brief mention of the novelty of the study (e.g., few studies have explored ClO₂ residuals and ingress in cold-chain sterilization)✓. Consider reordering subsections to improve flow.✓

For example:

- Materials✓
- Chlorine dioxide gas✓
- Sterilization process✓
- Validation process✓
- Residual and ingress testing✓

2. Include Figures 1–3 in results section to visually support the validation process.✓
3. Add a brief narrative to explain the significance of the results (e.g., why residual levels are acceptable, how they compare to EtO).✓
4. Highlight key quantitative findings (e.g., chlorite levels slightly above the EPA goal but below the maximum acceptable level).✓
5. Consider integrating some of the detailed results into the main text (e.g., key data points from Tables 5 and 6) to support the narrative.
6. Use subheadings more effectively to guide the reader through complex sections (e.g., within the Discussion).✓
7. Include additional visual aids (e.g., graphs, flowcharts) to illustrate key concepts (e.g., the sterilization process, comparison of ClO₂ and EtO).✓
8. Use concise, active language to improve readability (e.g., "We demonstrated" instead of "It was demonstrated").✓
9. Avoid redundancy (e.g., repetitive explanations of ClO₂ properties).

##### Author responses

- We added a subhead and sentence to the Introduction (per #1 above).
- We added quantitative results into the abstract to make it more meaningful (per #1 above).
- We added more to the implications of the study (per #1 above).
- We reordered the subsections to improve flow (per #1 above).
- We left the figures in the validation section because that is where they were used. (per #2 above).
- We added a subhead, regulatory information and a contextual sentence per #3 above.
- We reiterated that ingress testing was below both the contaminate goal and maximum by including those numbers in the narrative, per #4 above.
- We added subheads for clarity, per #6 above.
- We added a diagram per #7 above.

## 6. PLOS authors have the option to publish the peer review history of their article (what does this mean?).

If published, this will include your full peer review and any attached files. If you choose “no”, your identity will remain anonymous but your review may still be made public. Do you want your identity to be public for this peer review? For information about this choice, including consent withdrawal, please see our Privacy Policy.

- Reviewer #1: Yes
- Reviewer #2: No

[NOTE: If reviewer comments were submitted as an attachment file, they will be attached to this email and accessible via the submission site. Please log into your account, locate the manuscript record, and check for the action link "View Attachments". If this link does not appear, there are no attachment files.]

While revising your submission, please upload your figure files to the Preflight Analysis and Conversion Engine (PACE) digital diagnostic tool, https://pacev2.apexcovantage.com/. PACE helps ensure that figures meet PLOS requirements. To use PACE, you must first register as a user. Registration is free. Then, login and navigate to the UPLOAD tab, where you will find detailed instructions on how to use the tool. If you encounter any issues or have any questions when using PACE, please email PLOS at figures@plos.org. Please note that Supporting Information files do not need this step.
