# Supplementary material for: Chlorine dioxide gas sterilization of cold-chain or temperature-sensitive devices
Source: PLoS One. 2026 Aug 27;21(8):e0321345. doi: 10.1371/journal.pone.0321345 (PMC13521399; doi:10.1371/journal.pone.0321345)
Supplement: Supplementary file 5 [file pone.0321345.s005.docx]

**Missing Information for November 5, 2025 Revision**

Dear PLoS One Reviewers,

Sorry for the delay in responding. Trisha Mae T. Perez helped me understand what was still missing from the revision I submitted on December 18, 2025:

Dear Dr. Livingston,

Thank you for letting us know your concerns. After checking all the Technical Check Failure, you still need to amend the following. 

1. Please upload a Response to Reviewers letter which should include a point by point response to each of the points made by the Editor and / or Reviewers. (This should be uploaded as a ‘Response to Reviewers’ file type.) Please follow this link for more information: <http://blogs.PLOS.org/everyone/2011/05/10/how-to-submit-your-revised-manuscript/>

2. It is important that you include a cover letter with your manuscript. Please ensure that this letter is addressed specifically to PLoS ONE. Please also include
* why this manuscript is suitable for publication in PLoS ONE.
* how does your paper provide a worthwhile addition to the scientific literature?
* how does your paper relate to previously published work?
* which types of scientists do you believe will be most interested in your study?

Kindly re-submit paper on Editorial Manager once done with the Technical Check Failure.

Kind regards,

Trisha Mae T. Perez
Straive Editorial Assistant

PLOS ONE | [plosone@plos.org](mailto:plosone@plos.org)
Empowering researchers to transform science

Case Number: 09503464
thread::l-yp1d_Xf_qgWBgTs8Jyrho::

I have responded in this cover letter to each of the points below.

## Why is this manuscript suitable for PloS ONE?

This manuscript is suitable for PLoS ONE because it offers a novel perspective on a critical aspect of sterilization through the lens of an alternative sterilization modality. Low-temperature sterilization modalities will become increasingly important as heat- and moisture-sensitive biologics and combination products proliferate and regulatory and environmental pressures accelerate the industry’s transition away from EtO.

## How does this manuscript provide a worthwhile addition to the scientific literature?

This manuscript touches on an area of research that has largely been neglected. There is very little published scientific literature on ClO2 residual and ingress testing. ClO2 is a relatively new modality for terminal sterilization, and acceptable levels of residual contamination on medical devices after sterilization have not been formally defined. This manuscript presents a useful stepping stone for further research.

## How does this manuscript relate to previously published work?

As mentioned above, there is not a lot of previously published work on the topic of ClO2 residual and ingress testing. A similar paper on residuals from chlorine dioxide was published in 2025:

Lorcheim P, Lorcheim E, Van Ee T. Chlorine Dioxide Medical Device Sterilization: Residual Toxicity Testing. *Journal of Medical and Biological Engineering*. 2025;45(6):826-832. doi:https://doi.org/10.1007/s40846-025-00995-8

## What types of scientists do you believe will be most interested in this study?

Microbiologists and sterility validation scientists within companies that employ terminal sterilization will be among the most interested. Many medical device companies are seeking alternative terminal sterilization modalities to reduce reliance on ethylene oxide (EO), which is subject to increasing restrictions.

Thanks,

Kirk Livingston

# Response to Reviewers

## Most Recent Review Comments

Thank you for your article and effort in the field of sterilization of temperature-sensitive

products using chlorine dioxide gas. Your study in this area is very valuable and shows the areas of alternative sterilization with EtO. However, a few points are suggested to improve and strengthen the article:

1. The Materials and Methods section would benefit from a more detailed and

comprehensive description of the sterilization protocols and the residual and ingress testing procedures. This will improve reproducibility and clarity for readers.

2. Please include a dedicated section that clearly explains the statistical methods and data analysis approaches used in the study.
3. Results should be presented with more quantitative detail in the text, helping readers better understand the findings and their significance.
4. The Introduction would be stronger with a clearer statement of the study’s objectives and novelty to help frame the work’s contribution.
5. The Abstract should be revised to incorporate key quantitative results and major conclusions succinctly.
6. Consider revising the overall structure and organization of the manuscript to enhance logical flow and readability.
7. Language and style could be polished in places to improve clarity and reduce ambiguity.
8. Clearly discuss the limitations of the current study and suggest directions for future research.

Addressing these points will substantially enhance the quality and impact of your manuscript. I look forward to reviewing a revised version.

Best regards

## Point by Point Response to Most Review Comments

### Question 1

“1. The Materials and Methods section would benefit from a more detailed and

comprehensive description of the sterilization protocols and the residual and ingress

testing procedures. This will improve reproducibility and clarity for readers.”

We added more detail about the sterilization protocols and clarified how residual and ingress testing were conducted. We also gather the residual and ingress testing together into a single location. In addition, we added extensive information about the validation process, including a definition of the overkill method.

### Question 2

“2. Please include a dedicated section that clearly explains the statistical methods and data analysis approaches used in the study.”

We explained in the Data Analysis section that the study was not powered for formal statistical analysis and instead uses simple averages. Given the limited sample size, we did not perform inferential statistics. Nonetheless, the descriptive averages suggest important trends that merit attention.

### Question 3

“3. Results should be presented with more quantitative detail in the text, helping readers

better understand the findings and their significance.”

The Results section presents a straightforward comparison of average contaminant levels to maximum contaminant level goals and acceptable limits. Quantitative results are supplied in the text with tables that reinforce the conclusions. The section appears to have enough detail for readers to understand the findings and gauge their significance.

### Question 4

“4. The Introduction would be stronger with a clearer statement of the study’s objectives

and novelty to help frame the work’s contribution.”

We’ve improved the flow of the introduction by removing subheads and adding an explicit statement of the study’s objective. We also highlighted the study's novelty.

### Question 5

“5. The Abstract should be revised to incorporate key quantitative results and major

conclusions succinctly.”

We feel this has been addressed.

### Question 6

“6. Consider revising the overall structure and organization of the manuscript to enhance

logical flow and readability.”

We made several changes, including removing subheads, reorganizing the location of information, and using more formal language.

### Question 7

“7. Language and style could be polished in places to improve clarity and reduce ambiguity.”

We revised the document for a more formal tone throughout. We’ve also clarified ambiguities.

### Question 8

“8. Clearly discuss the limitations of the current study and suggest directions for future

research.”

We have noted the limitation of our sample size, in particular. We have suggested that subsequent studies would benefit from larger sample sizes.
