# Supplementary material for: Chlorine dioxide gas sterilization of cold-chain or temperature-sensitive devices
Source: PLoS One. 2026 Aug 27;21(8):e0321345. doi: 10.1371/journal.pone.0321345 (PMC13521399; doi:10.1371/journal.pone.0321345)
Supplement: Peer review history

# Peer review: Decision Letter 0

**DOI:** 10.1371/journal.pone.0321345.r001

**Authors:** Abbas Farmany

7 Apr 2025

Dear Dr. Livingston,

plosone@plos.org. When you're ready to submit your revision, log on to https://www.editorialmanager.com/pone/ and select the 'Submissions Needing Revision' folder to locate your manuscript file.

- A rebuttal letter that responds to each point raised by the academic editor and reviewer(s). You should upload this letter as a separate file labeled 'Response to Reviewers'.
- A marked-up copy of your manuscript that highlights changes made to the original version. You should upload this as a separate file labeled 'Revised Manuscript with Track Changes'.
- An unmarked version of your revised paper without tracked changes. You should upload this as a separate file labeled 'Manuscript'.

If you would like to make changes to your financial disclosure, please include your updated statement in your cover letter. Guidelines for resubmitting your figure files are available below the reviewer comments at the end of this letter.

If applicable, we recommend that you deposit your laboratory protocols in protocols.io to enhance the reproducibility of your results. Protocols.io assigns your protocol its own identifier (DOI) so that it can be cited independently in the future. For instructions see: https://journals.plos.org/plosone/s/submission-guidelines#loc-laboratory-protocols. Additionally, PLOS ONE offers an option for publishing peer-reviewed Lab Protocol articles, which describe protocols hosted on protocols.io. Read more information on sharing protocols at https://plos.org/protocols?utm_medium=editorial-email&utm_source=authorletters&utm_campaign=protocols.

We look forward to receiving your revised manuscript.

Kind regards,

Abbas Farmany

Academic Editor

PLOS ONE

Journal Requirements:

When submitting your revision, we need you to address these additional requirements.

1. Please ensure that your manuscript meets PLOS ONE's style requirements, including those for file naming. The PLOS ONE style templates can be found at

https://journals.plos.org/plosone/s/file?id=wjVg/PLOSOne_formatting_sample_main_body.pdf and

https://journals.plos.org/plosone/s/file?id=ba62/PLOSOne_formatting_sample_title_authors_affiliations.pdf

2. We note that the grant information you provided in the ‘Funding Information’ and ‘Financial Disclosure’ sections do not match.

When you resubmit, please ensure that you provide the correct grant numbers for the awards you received for your study in the ‘Funding Information’ section.

3. Thank you for stating the following financial disclosure:

“ClorDisys Solutions, Inc.”

Please state what role the funders took in the study. If the funders had no role, please state: "The funders had no role in study design, data collection and analysis, decision to publish, or preparation of the manuscript."

If this statement is not correct you must amend it as needed.

Please include this amended Role of Funder statement in your cover letter; we will change the online submission form on your behalf.

4. We note that your Data Availability Statement is currently as follows: All relevant data are within the manuscript and its Supporting Information files.

Please confirm at this time whether or not your submission contains all raw data required to replicate the results of your study. Authors must share the “minimal data set” for their submission. PLOS defines the minimal data set to consist of the data required to replicate all study findings reported in the article, as well as related metadata and methods (https://journals.plos.org/plosone/s/data-availability#loc-minimal-data-set-definition).

For example, authors should submit the following data:

- The values behind the means, standard deviations and other measures reported;

- The values used to build graphs;

- The points extracted from images for analysis.

Authors do not need to submit their entire data set if only a portion of the data was used in the reported study.

If your submission does not contain these data, please either upload them as Supporting Information files or deposit them to a stable, public repository and provide us with the relevant URLs, DOIs, or accession numbers. For a list of recommended repositories, please see https://journals.plos.org/plosone/s/recommended-repositories.

If there are ethical or legal restrictions on sharing a de-identified data set, please explain them in detail (e.g., data contain potentially sensitive information, data are owned by a third-party organization, etc.) and who has imposed them (e.g., an ethics committee). Please also provide contact information for a data access committee, ethics committee, or other institutional body to which data requests may be sent. If data are owned by a third party, please indicate how others may request data access.

5. Please amend your authorship list in your manuscript file to include author Dr. Kirk Livingston.

6. Please ensure that you refer to Figure 1 in your text as, if accepted, production will need this reference to link the reader to the figure.

[Note: HTML markup is below. Please do not edit.]

Reviewers' comments:

Reviewer's Responses to Questions

**Comments to the Author**

1. Is the manuscript technically sound, and do the data support the conclusions?

Reviewer #1: Partly

Reviewer #2: Yes

**********

2. Has the statistical analysis been performed appropriately and rigorously?

Reviewer #1: No

Reviewer #2: I Don't Know

**********

3. Have the authors made all data underlying the findings in their manuscript fully available?

The PLOS Data policy

Reviewer #1: No

Reviewer #2: Yes

**********

4. Is the manuscript presented in an intelligible fashion and written in standard English?

Reviewer #1: No

Reviewer #2: Yes

**********

Reviewer #1: The materials and methods section are poorly written with no coherent information explaining how the experiments were done. Although you stated all the materials used, the exact protocols or experiment processes are not elucidated in the write-up. Also, improve the discussion in totality by specifically stating how data analysis was done, and which statistical tools were used. Lastly, your data/results are not well explained and there is no information on how experiment is scientifically relevant.

Reviewer #2: Prepare abstract as more informative. In the abstract, include quantitative results (e.g., specific residual levels of chlorite, chlorate, and chloride) to make the findings more impactful. Briefly mention the implications of the study (e.g., potential as an alternative to EtO sterilization).

Provide a clearer statement of the study's objectives and hypotheses at the end of introduction.

Include a brief mention of the novelty of the study (e.g., few studies have explored ClO_2_ residuals and ingress in cold-chain sterilization).

Consider reordering subsections to improve flow.

For example:

Materials

Chlorine dioxide gas

Sterilization process

Validation process

Residual and ingress testing

Include Figures 1–3 in results section to visually support the validation process.

Add a brief narrative to explain the significance of the results (e.g., why residual levels are acceptable, how they compare to EtO).

Highlight key quantitative findings (e.g., chlorite levels slightly above the EPA goal but below the maximum acceptable level).

Consider integrating some of the detailed results into the main text (e.g., key data points from Tables 5 and 6) to support the narrative.

Use subheadings more effectively to guide the reader through complex sections (e.g., within the Discussion).

Include additional visual aids (e.g., graphs, flowcharts) to illustrate key concepts (e.g., the sterilization process, comparison of ClO_2_ and EtO).

Use concise, active language to improve readability (e.g., "We demonstrated" instead of "It was demonstrated").

Avoid redundancy (e.g., repetitive explanations of ClO_2_ properties).

**********

what does this mean?). If published, this will include your full peer review and any attached files.

If you choose “no”, your identity will remain anonymous but your review may still be made public.

**Do you want your identity to be public for this peer review?**  For information about this choice, including consent withdrawal, please see our Privacy Policy

Reviewer #1: **Yes**

Reviewer #2: No

**********

[NOTE: If reviewer comments were submitted as an attachment file, they will be attached to this email and accessible via the submission site. Please log into your account, locate the manuscript record, and check for the action link "View Attachments". If this link does not appear, there are no attachment files.]

While revising your submission, please upload your figure files to the Preflight Analysis and Conversion Engine (PACE) digital diagnostic tool, https://pacev2.apexcovantage.com/. PACE helps ensure that figures meet PLOS requirements. To use PACE, you must first register as a user. Registration is free. Then, login and navigate to the UPLOAD tab, where you will find detailed instructions on how to use the tool. If you encounter any issues or have any questions when using PACE, please email PLOS at figures@plos.org. Please note that Supporting Information files do not need this step.

# Author comment: Author response to Decision Letter 1

**DOI:** 10.1371/journal.pone.0321345.r002

5 Sep 2025

I have included many comments on the "Response to Reviewers" document. Thank you for your suggestions.

Attachment Submitted filename: ResponseToReviewers-PLOSOne-Revision1-20250522.docx

# Peer review: Decision Letter 1

**DOI:** 10.1371/journal.pone.0321345.r003

**Authors:** Abbas Farmany

5 Nov 2025

Dear Dr. Livingston,

Please submit your revised manuscript by Dec 20 2025 11:59PM. If you will need more time than this to complete your revisions, please reply to this message or contact the journal office at plosone@plos.org. When you're ready to submit your revision, log on to https://www.editorialmanager.com/pone/ and select the 'Submissions Needing Revision' folder to locate your manuscript file.

Please include the following items when submitting your revised manuscript:

- A rebuttal letter that responds to each point raised by the academic editor and reviewer(s). You should upload this letter as a separate file labeled 'Response to Reviewers'.
- A marked-up copy of your manuscript that highlights changes made to the original version. You should upload this as a separate file labeled 'Revised Manuscript with Track Changes'.
- An unmarked version of your revised paper without tracked changes. You should upload this as a separate file labeled 'Manuscript'.

If applicable, we recommend that you deposit your laboratory protocols in protocols.io to enhance the reproducibility of your results. Protocols.io assigns your protocol its own identifier (DOI) so that it can be cited independently in the future. For instructions see: https://journals.plos.org/plosone/s/submission-guidelines#loc-laboratory-protocols. Additionally, PLOS ONE offers an option for publishing peer-reviewed Lab Protocol articles, which describe protocols hosted on protocols.io. Read more information on sharing protocols at https://plos.org/protocols?utm_medium=editorial-email&utm_source=authorletters&utm_campaign=protocols.

We look forward to receiving your revised manuscript.

Kind regards,

Abbas Farmany

Academic Editor

PLOS ONE

**Journal Requirements:**

1. If the reviewer comments include a recommendation to cite specific previously published works, please review and evaluate these publications to determine whether they are relevant and should be cited. There is no requirement to cite these works unless the editor has indicated otherwise.

2. Please review your reference list to ensure that it is complete and correct. If you have cited papers that have been retracted, please include the rationale for doing so in the manuscript text, or remove these references and replace them with relevant current references. Any changes to the reference list should be mentioned in the rebuttal letter that accompanies your revised manuscript. If you need to cite a retracted article, indicate the article’s retracted status in the References list and also include a citation and full reference for the retraction notice.

**Additional Editor Comments:**

The authors conducted a well-designed and relevant study that addresses a critical gap in the sterilization of temperature-sensitive combination products. The data presented makes a compelling case for Chlorine Dioxide (ClO_2_) as a viable alternative to ethylene oxide, and the focus on both efficacy and safety (residuals/ingress) is particularly valuable for the industry. However, some points needs to be addressed prior its publication:

- The methods for residual and ingress testing are well-described, but some details are scattered between the "Preparation Summary" and "Lab Procedure" etc... sections. Consolidating and clarifying these would aid reproducibility.

- The "telegraphic" or elliptical style used in the "Lab Procedure" etc... subsections, while common in internal protocols, is non-standard for a published manuscript and can be jarring for the reader. Rewrite these sections using the full passive voice for a more formal and consistent academic tone.

- The text frequently references internal operating procedures (e.g., "OP-059," "OP-031"). While this is good practice internally, for an external audience, it is more important to describe the key parameters directly in the manuscript. Ensure that all critical cycle parameters (e.g., vacuum levels, exposure setpoint, PPM-hours) are explicitly stated in the main text, as is already done in point 8 of the "Lab Procedure—Ingress Testing." The reference to internal procedures can remain, but the core information must be in the manuscript itself.

- In the **Discussion** , the sentence "ClO_2_ also eases environmental and health concerns compared to EtO..." could be supported by a specific reference to EtO's classification as a carcinogen. Add a citation here, for example, to the ATSDR toxicological profile for ethylene oxide that is already in the reference list (Ref 19).

- Delete the content section from the text.

- No any subsection is needed in the Introduction section.

- Re-prepare the paper according to Journal format.

[Note: HTML markup is below. Please do not edit.]

Reviewers' comments:

Reviewer's Responses to Questions

**Comments to the Author**

Reviewer #2: All comments have been addressed

Reviewer #3: (No Response)

**********

2. Is the manuscript technically sound, and do the data support the conclusions?

Reviewer #2: (No Response)

Reviewer #3: Partly

**********

3. Has the statistical analysis been performed appropriately and rigorously?

Reviewer #2: (No Response)

Reviewer #3: No

**********

4. Have the authors made all data underlying the findings in their manuscript fully available?

The PLOS Data policy

Reviewer #2: (No Response)

Reviewer #3: No

**********

5. Is the manuscript presented in an intelligible fashion and written in standard English?

Reviewer #2: (No Response)

Reviewer #3: No

**********

**Reviewer #2:**  (No Response)

**Reviewer #3:** Dear Authors,

Thank you for submitting your manuscript entitled "Chlorine dioxide gas sterilization of cold-chain or temperature-sensitive devices". This study addresses an important topic in the field of chlorine dioxide gas sterilization of cold-chain or temperature-sensitive devices. The manuscript has several strengths, including the relevance of the research question and the experimental approach.

However, after a careful review, I have identified several areas that require significant revisions before this manuscript can be considered for publication:

1.Materials and Methods: The methods section lacks sufficient detail in parts. Please provide a more comprehensive description of the sterilization protocols, residual and ingress testing procedures, and the validation methodology used.

2.Statistical Analysis: The statistical approaches used in the study are not adequately described or justified. Please clarify the methods, ensure appropriate use of statistical tests, and provide supporting details such as sample size rationale.

3.Results Presentation: The results section should include more detailed quantitative data with clearer explanation of findings. Tables and figures need to be improved to enhance the clarity and impact of the data.

4.Language and Clarity: While the manuscript is generally comprehensible, the English requires editing for clarity, grammar, and style. Consider professional language editing to improve readability.

5.Data Availability: The manuscript currently does not clearly indicate full availability of the underlying data as required by PLOS ONE policies. Please provide access to all relevant data or justify any restrictions.

6.Discussion and Conclusion: The discussion section would benefit from a more critical interpretation of the results and clearer linking to the conclusions. Limitations should be explicitly acknowledged.

Overall, the manuscript has potential but requires major revision.

Thank you for your contributions to the field. I look forward to reviewing the revised manuscript.

**********

what does this mean?). If published, this will include your full peer review and any attached files.

If you choose “no”, your identity will remain anonymous but your review may still be made public.

**Do you want your identity to be public for this peer review?**  For information about this choice, including consent withdrawal, please see our Privacy Policy

Reviewer #2: No

Reviewer #3: No

**********

[NOTE: If reviewer comments were submitted as an attachment file, they will be attached to this email and accessible via the submission site. Please log into your account, locate the manuscript record, and check for the action link "View Attachments". If this link does not appear, there are no attachment files.]

To ensure your figures meet our technical requirements, please review our figure guidelines: https://journals.plos.org/plosone/s/figures

You may also use PLOS’s free figure tool, NAAS, to help you prepare publication quality figures: https://journals.plos.org/plosone/s/figures#loc-tools-for-figure-preparation.

NAAS will assess whether your figures meet our technical requirements by comparing each figure against our figure specifications.

Attachment Submitted filename: reviewer.pdf

# Author comment: Author response to Decision Letter 2

**DOI:** 10.1371/journal.pone.0321345.r004

17 Mar 2026

Dear PLOS One Reviewers,

Thank you for the opportunity to submit a revised version of this paper. In this version, we have:

• Gathered together the residual and ingress testing information into a single location.

• Swapped out the telegraphic style for a passive voice.

• Clarified procedures that had previously been cited but not explicated.

• Added clarifying citations.

Thank you,

Kirk Livingston

Attachment Submitted filename: MissingInformationToReviewers-PLOSOne-Revision2.1-20260311.docx

# Peer review: Decision Letter 2

**DOI:** 10.1371/journal.pone.0321345.r005

**Authors:** Abbas Farmany

24 Jun 2026

Chlorine dioxide gas sterilization of cold-chain or temperature-sensitive devices

PONE-D-25-11420R2

Dear Dr. Livingston,

We’re pleased to inform you that your manuscript has been judged scientifically suitable for publication and will be formally accepted for publication once it meets all outstanding technical requirements.

Within one week, you’ll receive an e-mail detailing the required amendments. When these have been addressed, you’ll receive a formal acceptance letter and your manuscript will be scheduled for publication.

An invoice will be generated when your article is formally accepted. Please note, if your institution has a publishing partnership with PLOS and your article meets the relevant criteria, all or part of your publication costs will be covered. Please make sure your user information is up-to-date by logging into Editorial Manager at Editorial Manager® and clicking the ‘Update My Information' link at the top of the page. For questions related to billing, please contact billing support.

If your institution or institutions have a press office, please notify them about your upcoming paper to help maximize its impact. If they’ll be preparing press materials, please inform our press team as soon as possible -- no later than 48 hours after receiving the formal acceptance. Your manuscript will remain under strict press embargo until 2 pm Eastern Time on the date of publication. For more information, please contact onepress@plos.org.

Kind regards,

Abbas Farmany

Academic Editor

PLOS One

Additional Editor Comments (optional):

Reviewers' comments:

Reviewer's Responses to Questions

**Comments to the Author**

Reviewer #2: All comments have been addressed

Reviewer #3: All comments have been addressed

**********

2. Is the manuscript technically sound, and do the data support the conclusions?

Reviewer #2: Yes

Reviewer #3: Yes

**********

3. Has the statistical analysis been performed appropriately and rigorously?

Reviewer #2: I Don't Know

Reviewer #3: Yes

**********

4. Have the authors made all data underlying the findings in their manuscript fully available?

The PLOS Data policy

Reviewer #2: Yes

Reviewer #3: Yes

**********

5. Is the manuscript presented in an intelligible fashion and written in standard English?

Reviewer #2: Yes

Reviewer #3: Yes

**********

Reviewer #2: All comments have been addressed. I haven't found any concerns about dual publication, research ethics, or publication ethics.

Reviewer #3: This is a straightforward and well-conducted study. The authors have addressed the previous concerns, and the manuscript is now clear and easy to follow. The methods are appropriate for the research question, the controls are adequate, and the results speak for themselves. I appreciate that the authors have been honest about the descriptive nature of the analysis and the study limitations.

I have no concerns about research ethics or publication ethics. The work appears original.

**********

what does this mean?). If published, this will include your full peer review and any attached files.

If you choose “no”, your identity will remain anonymous but your review may still be made public.

**Do you want your identity to be public for this peer review?**  For information about this choice, including consent withdrawal, please see our Privacy Policy

Reviewer #2: No

Reviewer #3: No

**********

# Editor report: Acceptance letter

**DOI:** 10.1371/journal.pone.0321345.r006

**Authors:** Abbas Farmany

PONE-D-25-11420R2

PLOS One

Dear Dr. Livingston,

I'm pleased to inform you that your manuscript has been deemed suitable for publication in PLOS One. Congratulations! Your manuscript is now being handed over to our production team.

At this stage, our production department will prepare your paper for publication. This includes ensuring the following:

* All references, tables, and figures are properly cited

* All relevant supporting information is included in the manuscript submission,

* There are no issues that prevent the paper from being properly typeset

You will receive further instructions from the production team, including instructions on how to review your proof when it is ready. Please keep in mind that we are working through a large volume of accepted articles, so please give us a few days to review your paper and let you know the next and final steps.

Lastly, if your institution or institutions have a press office, please let them know about your upcoming paper now to help maximize its impact. If they'll be preparing press materials, please inform our press team within the next 48 hours. Your manuscript will remain under strict press embargo until 2 pm Eastern Time on the date of publication. For more information, please contact onepress@plos.org.

You will receive an invoice from PLOS for your publication fee after your manuscript has reached the completed accept phase. If you receive an email requesting payment before acceptance or for any other service, this may be a phishing scheme. Learn how to identify phishing emails and protect your accounts at https://explore.plos.org/phishing.

If we can help with anything else, please email us at customercare@plos.org.

Thank you for submitting your work to PLOS One and supporting open access.

Kind regards,

PLOS ONE Editorial Office Staff

on behalf of

Dr. Abbas Farmany

Academic Editor

PLOS One
